# The effect of protein supplementation on body muscle mass and fat mass in post-bariatric surgery: a randomized controlled trial (RCT) study protocol

**DOI:** 10.1186/s13690-017-0252-2

**Published:** 2018-01-22

**Authors:** Sahar D. Al-Shamari, Mohamed Aly ElSherif, Wahiba Hamid, Fahad Hanna

**Affiliations:** 1 Hamad Medical Corporation, Doha, Dawha, Qatar; 2 Department of Public Health, College of Health Sciences, Qatar University, P.O. BOX: 2713, Doha, Qatar

**Keywords:** Obesity, Weight loss, Muscle mass, Bariatric surgery, RCT

## Abstract

**Background:**

Severe weight loss through means of bariatric surgery has been associated with loss of muscle mass due to lack of absorption of protein. The aim of this RCT is to investigate the effectiveness of protein supplementation in reducing the risk of developing protein malnutrition and muscle wasting in post-bariatric surgery patients in Qatar.

**Methods and analysis:**

The study was based at the Department of bariatric and metabolic surgery, Doha metropolitan and regional areas. It is envisaged that approximately 160 post-bariatric surgery patients will be randomized and followed up for 6 months. These will be males and females obese (BMI >35) Qatari patients between the aged 18–60 years. Subjects with renal or liver disease and those with past history of bariatric surgery will be excluded. By the completion of the trial, patients who took less than 80% of the supplement will be further excluded from the final analysis. Protein supplement (Cubitan,Protein, Nutricia, Netherlands) that contain daily intake of 20 g of protein to be taken orally 3 times a day throughout the study period. The placebo group will receive identical ampule containing zero-protein with exact instructions as per the intervention group. Body weight, muscle and fat mass, total protein, albumin, vit B12, Magnesium and Zinc will be measured at baseline and every follow up/study visit. Study variables will be compared between the 2 groups at different stages of the trial, including baseline, using Sample T-test (paired and unpaired) and the significance level will be confirmed with the 95% confidence interval with alpha error set to 0.05.

**Ethics and dissemination:**

Protein supplementation for post-bariatric patients is not yet a standard procedure at Hamad Medical Corporation in Qatar and requires an RCT to establish evidence-based clinical practice guidelines. This study was approved by the Hamad Medical Corporation IRB and MRC committees (approval no. 16433/16).

**Trial registration:**

ClinicalTrials.gov NCT03147456 (registration date: 18 April 2017).

**Strengths and limitations of this study:**

One major strength of our study is that our population is a distinctive population (Qatari Obese patients) where results from international studies may not apply to the local and unique context. A local study like ours will provide healthcare providers in Qatar an opportunity to ensure good clinical practice and healthy and sustainable weight loss following bariatric surgery.

The well-designed double-blinded RCT will almost certainly provide us with the evidence-based clinical practice guideline that we seek as health professionals.

One limitation of our study is the slight discrepancy in caloric content of the intervention and the placebo (250 cal and 100 cal, respectively). However, it is the intervention that has the higher caloric content, in which case it may not influence the results in the direction of our hypothesis that protein supplementation leads to lower fat mass and higher muscle mass.

Another limitation is that the use of the intervention and the placebo are not objectively measured. However, all efforts will be made to ensure compliance and reporting of consumption of products.

A third limitation could be loss to follow up. Participants may cease to participate, particularly, once they have lost “sufficient’ weight and gained the fitness to consume any type of foods they desire. This is common in late stages of post-bariatric surgery (beyond 3 months). We feel that this may be a challenge, particularly in reference to our specific population. However, such findings albeit negative, should serve in improving the clinical practice delivered by healthcare providers.

**Electronic supplementary material:**

The online version of this article (10.1186/s13690-017-0252-2) contains supplementary material, which is available to authorized users.

## Background

Obesity is a chronic medical condition characterized by an accumulation of excess fat in the body that may lead to negative health consequences. It is the major contributor to some of the most prevalent chronic conditions, such as type 2 diabetes (T2D), heart and kidney diseases, metabolic syndrome, sleep apnea, and depression (National Institutes of Health, [5]; Ogden, 2007). Obesity is the fifth leading risk factor for deaths globally, making it an extremely important public health issue [6]. In 2014, a World Health Organization (WHO) report showed that more than 1.9 billion adults aged 18 years and older were overweight with over 600 million being obese (see Table 1 for obesity classification). In addition, 44% of the diabetes burden, 23% of heart disease burden, and 7–41% of certain cancer burdens are associated with overweight and obesity [10]. Around 2.8 million people die each year as a result of being overweight or obese. The majority of these deaths is premature and preventable and may apply an economic burden on the health systems [7].

**Table 1:** World Health Organization Body Mass Index (BMI) classification for adults

	Principal cut-off points	Additional cut-off points
Underweight	<18.50	<18.50
Severe thinness	<16.00	<16.00
Moderate thinness	16.00–16.99	16.00–16.99
Mild thinness	17.00–18.49	17.00–18.49
Normal range	18.50–24.99	18.50–22.99
		23.00–24.99
Overweight	≥25.00	≥25.00
Pre-obese	25.00–29.99	25.00–27.49
		27.50–29.99
Obese	≥30.00	≥30.00
Obese class I	30.00–34.99	30.00–32.49
		32.50–34.99
Obese class II	35.00–39.99	35.00–37.49
		37.50–39.99
Obese class III	≥40.00	≥40.00

Source: Adapted from WHO, 1995, WHO, 2000 and WHO 2004

Attempts to reverse obesity through weight loss have been the focus of the attention of many, including researchers, scientists and clinicians. This has been chiefly achieved via dietary and lifestyle modification, however, when these have not been successful, surgical options have been adopted for morbidly obese patients (ASMBS). Bariatric surgery (BS) has been shown to be the most effective type of approach to achieve and sustain significant weight loss in morbidly obese people [9]. BS is defined as a group of surgical procedures performed to facilitate substantial weight loss by reducing the size of the stomach and/or limiting absorption in the small intestine (American Society for Metabolic and Bariatric Surgery [1]. It is considered a highly efficacious treatment for extreme obesity. However, bariatric surgery patients frequently report difficulty initiating and maintaining healthy behavioral changes following surgery [4]. Most Post-bariatric surgery patients are strictly placed on liquid diet during the early post-operative phase and are practically unable to consume large quantities of food in one sitting or taking solid protein within the first months, which put them at a higher risk of developing protein malnutrition [8].

Post-surgery multivitamin and high protein supplementation is important to avoid any nutrient deficiency [3]. High protein supplements are mainly rich in protein and supplemented with vitamins and minerals. These supplements in general are inexpensive, widely distributed, and commonly used by people or patients who need nutritional supplementation while recovering from an illness or post-surgery especially post-bariatric surgery [3].

Protein supplementation is an effective approach to ensuring that post-bariatric surgery patients maintain muscle mass and healthy levels of the above-mentioned nutrients and body compositions [2]. In the absence of this, the weight loss achieved by the surgery may present a systematic issue leading to higher fat percentage and lack of these important elements that are essential for the function of the human physiology. The imbalance of such essential body compositions and nutrient can also lead to pathological and irreversible conditions. The objective of the present study is to examine the effectiveness of protein supplementation in reducing the risk of developing protein malnutrition that lead to low muscle mass and low protein level, in post-bariatric surgery patients.

### Study objectives

#### Primary objective

To estimate the difference in change of muscle and fat mass between participants who received protein supplement and those who received the placebo.

#### Secondary objectives

- The percentage of weight reduction in the intervention in comparison with control group
- The difference in protein level (albumin/total protein) in post-bariatric patients receiving intervention compared with control group
- The blood level changes in Vit B12, Magnesium and Zinc in the intervention group in comparison with the control group
- The differences in the above changes in relation to types of bariatric surgery

#### Study hypothesis

Protein supplementation in Qatari post-bariatric patients may improve or prevent the imbalance in the body’s essential proteins and nutrients and avoid muscle wasting and other undesirable health conditions.

## Methods and design

This study is a double-blinded randomized control trial where both investigators and participants will be blinded to the treatment (intervention and placebo products are identical and unlabeled). The researchers will recruit participants early 2017. The randomization will be done on a weekly basis within blocks of 8 or 10 patients. This is roughly the number of patients available per week. A simple randomization process will be employed to assign patients to either groups- intervention & placebo. Using a computer generated random list, the treatment and the placebo will be allocated in a random manner to numbers that label individual patients. For the blinding, a dietician will assign the treatment and the control to patients as per the randomization results.

Both the intervention and control groups will receive standard dietary instructions in post-bariatric surgery period including hospital stay (1–2 days) and every month thereafter. The intervention group will receive protein supplement and the placebo group zero-protein supplement everyday over the study period (6 months). The placebo & the protein supplement slightly differ in the caloric content (the difference is 150 kcal/bottle). Although this difference is not clinically significant, particularly for the early post-operative phase, this issue will be addressed as a limitation for this study.

Investigators and researchers, including providers of care (rehab staff) in this study will be blinded to the intervention, i.e. they will not be aware of who is on protein supplement and who is on placebo. Patients will also be blinded to the intervention, i.e. they will not be aware whether they are taking protein or zero protein supplement. This dietitian is also responsible for filling the intervention & control ready-to-feed shakes into identical bottles labeled either A or B. The dietician will not have any other responsibility such as data entry or follow up of participants, as this will be done by the PI and research team. Un-blinding will be unlikely within the settings of our trial, however, any suspicion of un-blinding will be dealt with appropriately and may result into the cessation of participation in the trial.

### Study participants

The recruitment of participants for this RCT is based at Hamad General Hospital. Patients who will undergo bariatric surgery and fulfill our inclusion and exclusion criteria will be randomized to either the intervention or the control arm of the study. The catchment area for Hamad General Hospital can include the entire Doha district and the surrounding metropolitan areas. However, admission to the hospital may also include other regional areas, thereby making the sample of our population representative of the entire Qatari population.

### Inclusion criteria

Study participants had the following criteria in order to participate in the trial:

1. Qatari males or females.
2. Aged between 18 and 60 years
3. Based at the bariatric surgery list of Hamad Medical Corporation (HMC) with their follow up scheduled be at HMC (see appendices).
4. Patients who consent to participate in the 6 months trial

### Exclusion criteria

Patients will be excluded from participating in the trial if they have the following criteria:

1. Any Renal or liver disease because that may affect protein or albumin level in body.
2. Past history of bariatric surgery (Gastric Band & Gastric bypass)
3. Patients will be further excluded after starting the trial if they did not take at least 80% (minimum 24 bottles per month) of their allocated intervention or placebo products throughout the trial.

### Sample size

The study population are bariatric patients admitted to bariatric department for surgery based on their eligibility criteria (BMI ≥ 35). All patients will be enrolled in the study prior to admission and will be given the intervention or the placebo on the first day post-surgery, depending on their randomization. A total of 160 obese Qatari adults from Bariatric and Metabolic Center in Hamad General Hospital (HGH), who fulfill HGH entry criteria for bariatric surgery, are expected to be enrolled in this experimental study.

Using the primary outcome estimates available in the published study (Schollenberger, A E et al.; 2016), body fat mass (BFM) and lean body mass (LBM) (kg) in the intervention group at baseline, 3 and 6 months (BFM baseline 78.2 ± 19.1, 3 and 6 months 59.9 ± 15) and (LBM baseline 65.2 ± 14.2, 3 and 6 months 58.2 ± 10.6) compared with Placebo group (BFM baseline 68.2 ± 12.0, 3 and 6 months 53.9 ± 11.5) and (LBM baseline 68.9 ± 13.4, 3 and 6 months 62.2 ± 11.3). Considering a 90% power and a type I error rate of 5%, the required sample size would be 130 participants (65 in each group), however, to compensate for possible dropouts, non-compliance and loss to follow-up an additional 15% to 20% will be added to the above sample size (a total of 160 participants i.e., 80 in each group). The only difference between the above study population and the population of our RCT is that the German study population were more obese (BMI between 49 and 52). Both studies looked at the effect of protein supplementation on muscle and fat mass in post-bariatric surgery patients.

### Intervention and control

#### Intervention

1. All participants will receive nutritional counseling by a bariatric dietitian as a routine standard of care, which includes strictly fluid diet for the first months. (Additional file 1).
2. Before discharge from the hospital, patients will be provided with the supplement and will be advised by the dietitian regarding its use (one can per day, over 3–5 intervals, i.e. the bottle will be used over the entire course of the day through a number between 3 and 5 shots/doses).
3. Each supplement (bottle) contains 20 g of protein, 250Kacl plus different micronutrient and macronutrient, per 200 ml can (Cubitan,Protein, Nutricia, Netherlands) (Additional file 2).

#### Control (placebo)

1. All participants will receive nutritional counseling by a bariatric dietitian as a routine standard of care, which includes strictly fluid diet for the first months. (Additional file 2).
2. Before discharge from the hospital, patients will be provided with the supplement (placebo) and will be advised by the dietitian regarding its use (one can per day over 3–5 intervals).
3. Following hospital discharge, Control patients will receive supplement that contains 0 g protein, fat free, 100 kcal and enriched with electrolytes per 200 ml can (preOp, Nutricia, Netherlands) (Additional file 2).

*Note: Both groups will be instructed to take one supplement pack through the day in equally divided portions, and, they should store it in room temperature and drink it without heating.

#### Materials

The protein supplement, a familiar treatment option at HMC, consists of whey protein isolate with the following characteristics:

- Pure Whey Protein Isolate.
- Whey protein isolate is the most pure and concentrated form of whey protein available.
- Whey protein isolate contains higher protein content (90–95%) and less lactose (<0.5%). It is a complete protein with all the indispensable essential amino acids and is an exceptional source of branch chain amino acids (BCAAs), especially leucine, necessary for tissue growth.
- Whey protein is a soluble, easy to digest protein and is efficiently absorbed into the body (Marshall, 2004).
- Aside from amino acids, other biological components of whey protein include lactoferin (concentration is commonly 0.35–2.0% of total proteins), immunoglobulins (10–15% of total whey proteins), lactoperoxidase (accounts for 0.25–0.5% of total protein in whey), glycomacropeptide (with 10–15%), and bovine serum albumin (10–15% of total whey protein). These bioactive components together with the essential and non-essential amino acids help improve body mass index especially in individuals doing exercise programs, by enhancing muscle mass and reducing fat mass. (Marshall, 2004).
- A 200 ml intervention can contains 20 g of protein, 250Kacl plus various micronutrient and macronutrient.
- A 200 ml placebo can contains 0 g protein, fat free, 100 kcal and enriched with electrolytes, per can (200 ml).

#### Measurements

1. Baseline measurement of body composition (fat mass and muscle mass), height and weight will be conducted on day one of the trial, using Tanita body composition instrument and other physical body measures.
2. Baseline blood test for total protein, albumin, Vit B12, Zinc and Magnesium levels will be collected.
3. All of the above measurements will be repeated at 1 month, 3 months and at the end of the follow up period (6 month).

*Note: All of the above measurements and the collection of blood sample is routine practice for post-bariatric surgery patient.

#### Study visits

Patients follow up visit once every month post-surgery at the Bariatric Dietician Clinic in OPD department at HGH, and covers the following:

A. Post-surgery dietary advice, to sustain a hypo caloric and protein-rich diet.
B. Anthropometric parameters for body composition (see below).
C. All patients will be followed up through weekly phone calls to ensure compliance and adherence to the study protocol. Questions such as “how many bottles of supplements are left?” Or, “how many bottles have you had so far?” will be asked. Patients will be asked to use a diary to register their daily use of the products as well as fill the forms provided by the hospital in relation to consumption of supplement.
D. Collection of blood sample will take place following an overnight fasting. All blood markers will be measured and calculated in the central laboratory at HGH.

#### Body composition (BC)

The measurements of anthropometric data will be done following overnight fast with patients wearing light clothes and barefoot (baseline and follow up). Tanita’s body composition analyzer used for body composition measurements provides precision in estimating body Weight, Body Fat Percentage, Body Fat Mass, Body Mass Index (BMI), Fat Free Mass, and Estimated Muscle Mass and percentage. Height will be measured using a fixed wall stadiometer. Both inter- and intra-reliability testing on all measures, including TanitaHealthWare, are conducted prior to the trial to ensure consistency of the measures across the study population. This is a standard hospital procedure and these tests will ensure that the assessment tools produced are consistent and reliable measurements.

#### Blood sample

Blood samples will be collected from patients in (HGH) laboratory at baseline and every month thereafter following an overnight fast. Complete blood count (CBC) will be used. Total protein, serum Albumin, zinc, magnesium and vitamin B12 will also be assessed. The laboratories of HGH are internationally accredited laboratories that follow international standards.

#### Data collection and handling

All data obtained from this study will be collected by the principal investigator and will be saved in a secure location where only the project supervisors and a statistician will be able to access. Data will be de-identified during and post collection period to ensure privacy and ethical protocols are maintained. All data will be stored and kept safe for a period of time according to HMC policy and will remain a property of HMC. The actual physical location of all electronic copies will also be at HGH. Confidentiality of patients will be highly maintained throughout the trial.

#### Study timeline

All participants will be followed up for 3 and 6 months from day one of their participation. Therefore, each randomized pair will have their own follow-up dates, based on the recruitment pattern at the HGH. For example, a patient who enters the trial on the 3rd May, he or she will have the 3 months follow up date on the 3rd August and the 6th months follow up date on the 3rd November.

## Statistical analysis

Data analyses will be performed using the statistical software SPSS package 23. The main aim is to analyze Body composition and other anthropometric changes throughout the follow up. The normality of the variables will be assessed by the Kolmogorov-Smirnov test. Non-parametric tests will be used in case of non-normal distributions of variables. Characteristics and demographics of participants will be summarized, based on the assignment of treatment or placebo, using means and standard deviations (SD) for quantitative variables. Baseline data for the two groups will be compared using two tailed independent samples t-tests and the *P* value of this difference for each variable will be provided. During the follow-up, changes in weight, body fat percentage, muscle mass, total protein level, albumin level, vit B12, Zinc and Magnesium will be compared within groups using paired t-test and between groups using independent sample t-test. In the case of non-normal distribution of variables, any two independent (unpaired) samples Student’s t test will be replaced by Mann-Whitney U test. However, in the case of Two dependent (paired) samples Student’s t test, Wilcoxon signed rank (paired samples) test will be used. A multiple linear regression model will also be conducted to adjust for age and gender will be conducted. Other fundamental confounders such as diet intake are not incorporated into the multiple binary regression model. This is primarily because post-bariatric patients within the 1st few months (early post-operative phase) are both unable and not allowed to take any solid foods and few specific liquid foods such as milk and soups are recommended to them by the dietician. All patients are trained to follow such diet. Moreover, being an RCT, it is expected that all confounders (including hidden confounders) are balanced between the 2 groups and no assessment of baseline variables such as diet, physical activity and any other biological differences between the 2 groups is required. This is consistent with the CONSORT statement for RCTs.

### Ethical consideration

1. Patients have the right to refuse participating in the study.
2. Patients have the right to withdraw from the study at any time.
3. All patients will be asked to provide consent to participate by signing the “patient consent form”, as an agreement to enter the study (Additional file 3).
4. Researchers and other health-care professionals (investigators) will hold all information about the patients in strict confidence and disclose it only to those who have a legal right to this information. Moreover, patient visits will be conducted in private rooms.
5. Any information that might lead to the identification of individual subjects will be omitted.
6. De-identification of data and confidentiality of patients will be highly maintained throughout the trial
7. “Intervene to protect patients” approach will be implemented if researchers are highly suspicious that a risk or abnormal lab value is imminent, and if necessary, staff will seek advice from more experienced professionals/clinicians.

Ethical approval was requested from both HMC and Qatar University IRB committees. Both committees granted the approval thereby confirming the legitimacy of our study. The RCT approval number from HMC is 16,433/16 (Additional files 4, 5).

### Trial registration

Our clinical trial was registered with ClinicalTrials.gov (US-NIH) PRS (protocol registration and results system), ID number: NCT03147453.

## Additional files

Additional file 1: (PDF 7359 kb)

Additional file 2: (PDF 1097 kb)

Additional file 3: (PDF 129 kb)

Additional file 4: (PDF 251 kb)

Additional file 5: (PDF 439 kb)
