# Supplementary material for: The effect of protein supplementation on body muscle mass and fat mass in post-bariatric surgery: a randomized controlled trial (RCT) study protocol
Source: Arch Public Health. 2018 Jan 22;76:7. doi: 10.1186/s13690-017-0252-2 (PMC5789587; doi:10.1186/s13690-017-0252-2)
Supplement: Supplementary file 1 — (PDF 7359 kb) [file 13690_2017_252_MOESM1_ESM.pdf]

TANITA®
Monitoring Your Health

Eng

BODY COMPOSITION ANALYZER

SC-331S

Instruction manual

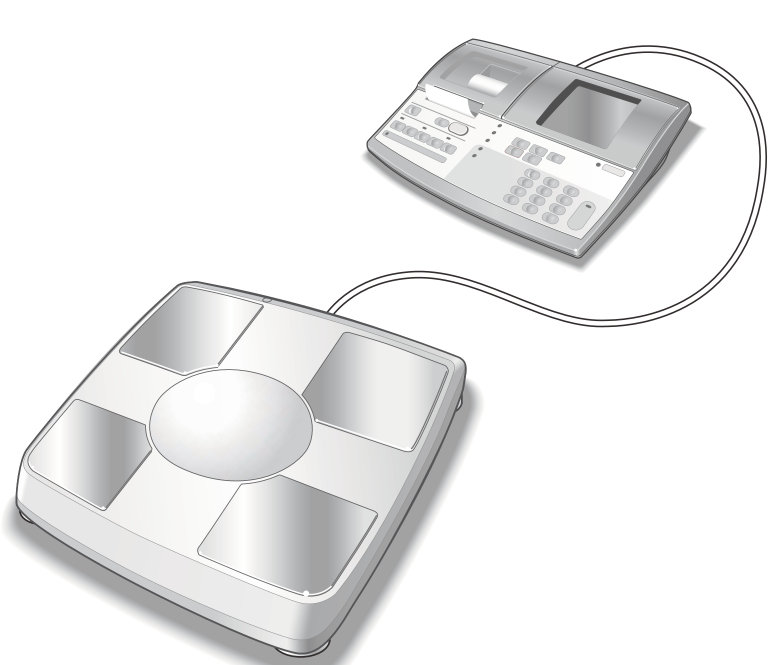

SC-331S

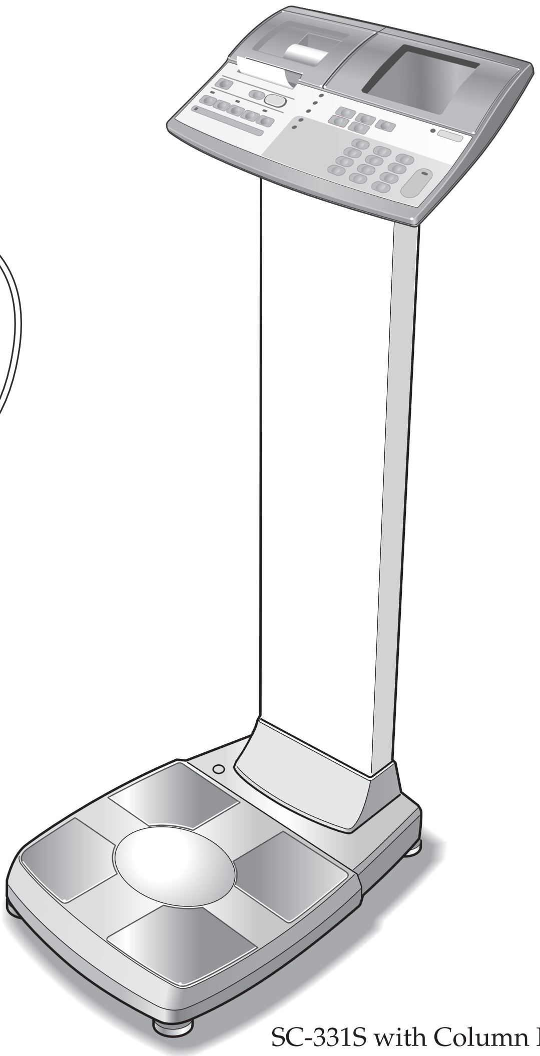

SC-331S with Column Kit

<Usage Conditions>

Temperature Range for Use	:	**0°C – 35°C**
Relative Humidity	:	**30% – 80%** (without condensation)
Max Altitude	:	**2,000m ASL**
Pressure Range of Environment	:	**86kPa - 106kPa**

<Storage Conditions>

Temperature Range of Environment	:	**-10°C – 60°C**
Range of Relative Humidity	:	**10% – 90%** (without condensation)

To avoid malfunctions, avoid storing the equipment where there is direct sunlight, significant temperature changes, the risk of dampness, a large amount of dust, in the vicinity of fires, or where there is the risk of receiving vibrations or shocks.

⚠ Please read this Instruction Manual carefully and keep it handy for future reference.

Eng

Intended use

- This equipment can be used in the screening of certain adult diseases and conditions related to body weight and composition.
- It can be used in the monitoring and prevention of conditions caused by excessive deposits of fat tissue such as diabetes, hyperlipidemia, cholelithiasis and fatty liver.
- It can be used in the monitoring of changes in individuals' body composition related to differences in the ratio of fat tissue to lean.
- It can be used to assess the effectiveness of individuals' nutrition and exercise programmers, both for health and physical fitness.
- The TANITA Body Composition Analyzer is indicated for use in the measurement of weight and impedance , and the estimation of body mass index (BMI), total body fat percent, total body water percent and weight , muscle mass (skeletal and smooth), physique rating, bone mass, visceral fat rating with healthy range, basal metabolic rate (BMR), metabolic age, and target body fat percent with predicted weight and fat mass, using B IA (Bioelectrical Impedance Analysis).
  The device is indicated for use for healthy children 5-17 years old and healthy adults with active, moderately active, to inactive lifestyles.

Efficacy

1. This product is simple to use, and requires no specialized facilities or expertise to take measurements.
2. Measurements can be taken quickly and easily, causing minimal inconvenience to the patient during measurments.

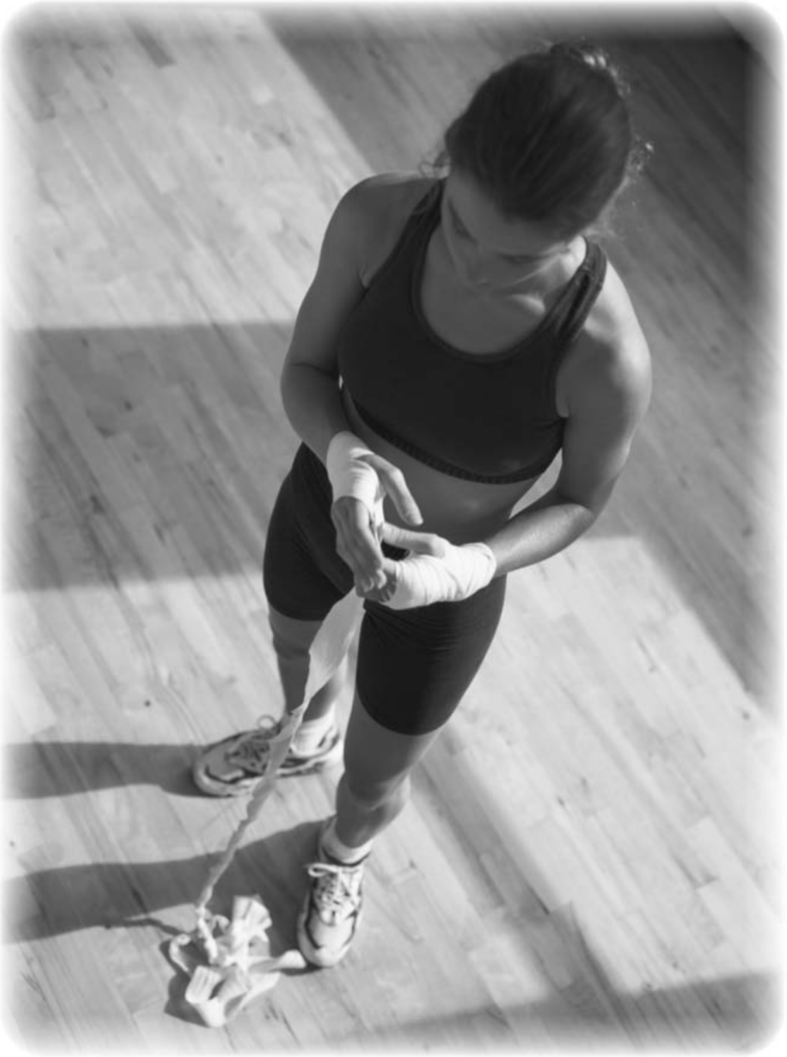

Eng

Contents

Before use

Safety Notes ······ 4
Product Assembly and Components ······ 8
Preparation ······ 11
Various settings ······ 12

How to use

Operating Instructions ······ 28
- When using as a body composition analyzer ······ 28
- Target body fat % ······ 38
- when using as a scale ······ 39

Various criteria ······ 42

When necessary

Troubleshooting ······ 48
Connection with a personal computer ······ 50
Technical Notes ······ 54
The New Regression Formula for Basal Metabolic Rate (BMR) ······ 58
Specifications ······ 60

For Your Safety

The following outlines precautionary measures to be taken to avoid injury to the users of this device and others, and to prevent damage to property. Please familiarise yourself with the contents to ensure the safe operation of this equipment.

⚠ Warning	Ignoring instructions highlighted with this mark could result in death or severe injury.
⚠ Caution	Ignoring instructions highlighted with this mark could result in injury or damage to property.
⃠	This mark indicates actions that are prohibited.
!	This mark indicates instructions that must always be adhered to.

⚠ Warning

Prohibited

This device must not be used on subjects with pacemakers or other mechanical implants.
This device passes a weak electrical current through the body which could interfere with and cause the malfunction of electrical medical implants, resulting in serious repercussions.

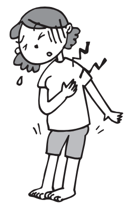

Do not handle the plug with a wet hand.
Such action may result in electric shock, fire, or leakage.

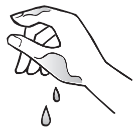

Keep this equipment away from flammable gas and oxygen rich environment.

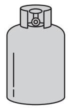

No modification of this equipment is allowed.
There is a risk of electrocution or injury. Nor can precise analysis be guaranteed.

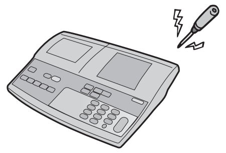

Do not use double adapters.
May result in fire.

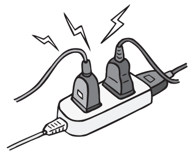

Always…

Use only a correctly wired outlet.
Only genuine cables and equipments are allowed.

⚠ Caution

Prohibited

Keep off wet circumstance.

Avoid using on subjects with allergies to metals.
Allergic reactions may be caused by the stainless steel used in the electrodes of this device.

Do not jump on the equipment.

Do not lean on the equipment.
Do not use this equipment near other products that emit electromagnetic waves.

Do not insert fingers into gaps and holes.

Avoid impacts on the display.
The screen panel may split and cause injury.

Ensure disabled persons do not attempt to use the equipment by themselves.
A companion must perform the measurements.

Always…

Be sure to clean the scale platform with appropriate disinfectant after each use.

For your safety to avoid the risk of electric shock and to secure the accuracy, keep a clearance with patients during measuring.

Continually monitor both the subject and the equipment for anomalies.
Should an anomaly with the subject or the equipment be discovered, take appropriate action, such as stopping the equipment, while ensuring the safety of the subject.

Unplug the equipment from the wall outlet when it will not be in use for long periods of time.

Do not interpret analysis results yourself (including evaluating measurements and formulating exercise programmes based on results, etc.).
Weight loss and exercise based on self-analysis could be detrimental to you health. Always follow the advice of a qualified professional.

This equipment is designated a Class B IT equipment (mainly for systems intended to be used in domestic environments) and CE (EMC), but weak equipment may be affected.
If you connect your computer or peripherals, please use the compliance with IEC60601-1 (EN60601-1). Or if using the compliance with IEC60950 (EN60950), power must be supplied from themedical isolating transformer. Keep at a distance of 1.5m between each equipment during operation. If you ignore this requirement, may cause electrical shock of patients or operation.

Eng
Before use (For Your Safety)

For Your Safety

For Accurate Measurements

Avoid measuring after hard exercise.

May result in measurement errors. Please take measurements after sufficient rest.

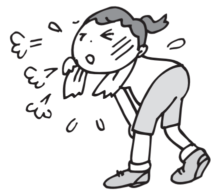

Avoid measuring after over-eating or over-drinking, and when severely dehydrated.

May result in measurement errors. For greater accuracy, avoid using directly after waking up. Use at the same time on each occasion, at least three hours after eating.

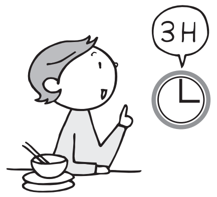

Do not take measurements while using transmitters, such as mobile phones, which may affect readings.

Use the device under the same conditions and in the same position as much as possible to track changes.

Readings are greatly affected by the level of hydration and position of the body. Please use at the same time each day, under the same conditions and in the same body position.

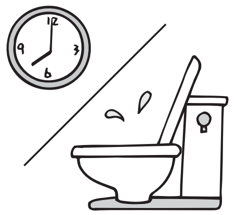

Avoid measuring in locations of greatly differing temperature.

May result in measurement errors. When the equipment has been moved to a location ±20ºC, allow to stand for at least 2 hours before using.

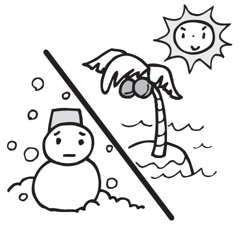

Bare feet should be placed correctly on the electrode panel for measurement.

Also, make sure the soles of feet are free of excess dirt, as this may also act as a barrier to the mild current.

Use in a stable location.

Errors in measurements may occur when the device is used in an unstable location.

Product Assembly and Components

SC-331S

Control Box

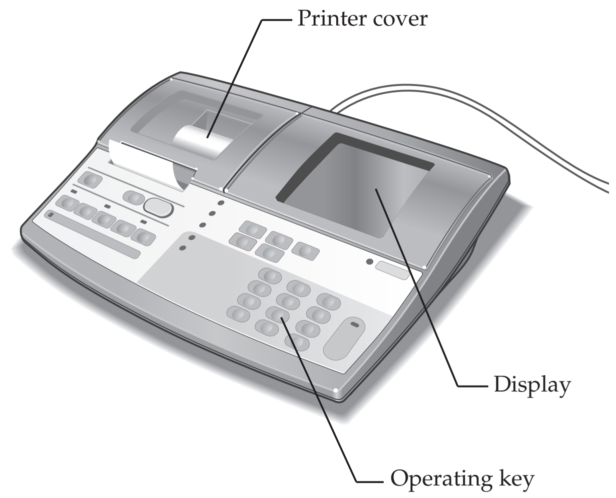

Platform

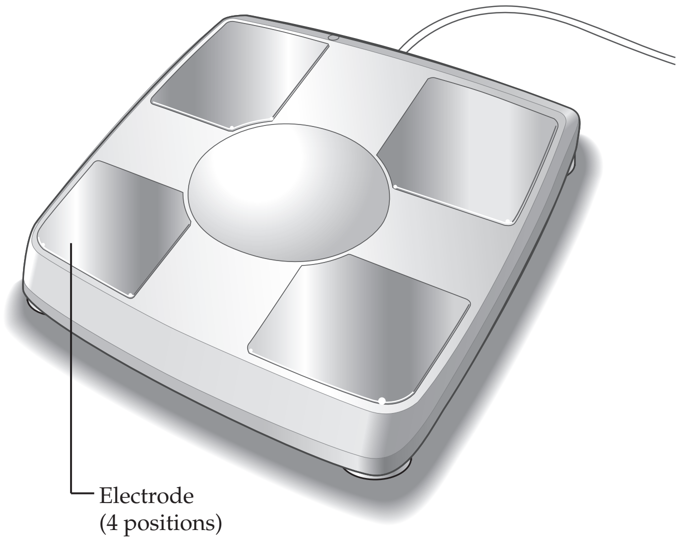

Accessories

- ☑ Instruction manual (This manual)
- ☐ Quick reference guide
- ☐ CD-ROM (USB driver)
- ☐ AC adapter
- ☐ Power cord

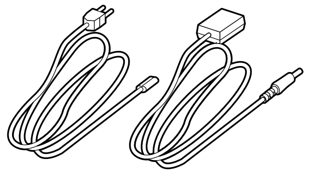

- ☐ Dropper (1 item)

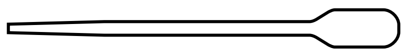

- ☐ Printer paper
  (Thermal paper, roll diameter: 2.2in / 55mm, roll length: approx. 112ft / 34m)
  * Please contact the agent from which you have purchased the product for details.

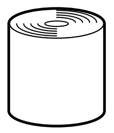

⚠Caution Make sure you place the Platform on a stable, level surface.If the Platform is not stable because not all the feet are on the surface, for example, there is a risk of stumbling or inaccurate measurement.

SC-331S with Column Kit

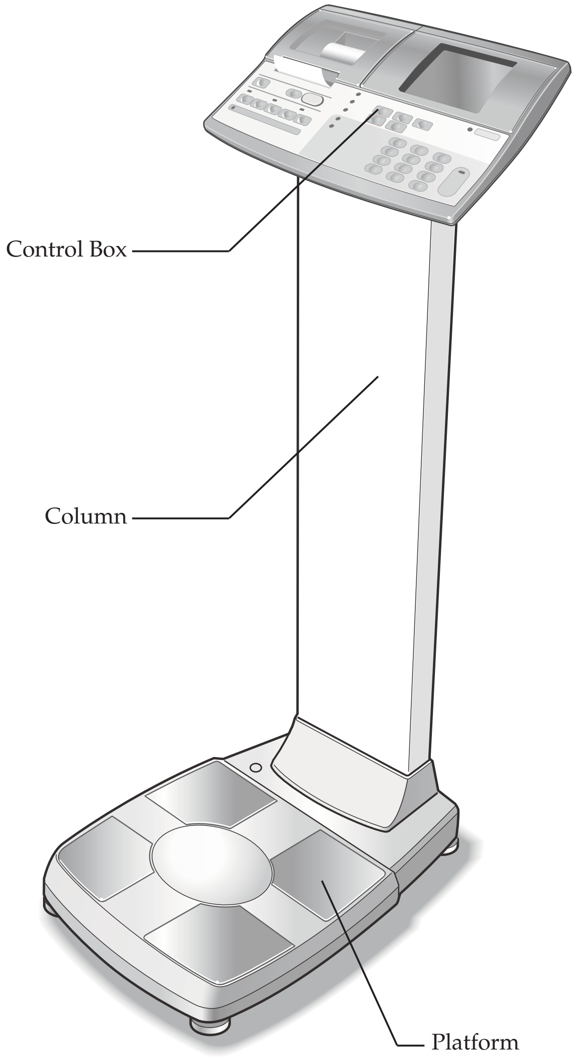

Accessories

- ☑ Instruction manual (This manual)
- ☐ Quick reference guide
- ☐ CD-ROM (USB driver)
- ☐ Assembling guide
- ☐ AC adapter
- ☐ Power cord

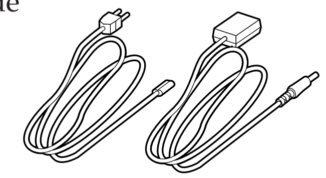

- ☐ Hexagonal wrench (1 item)
- ☐ Hexagon socket head bolts (M5L12) (4 items)
- ☐ Dropper (1 item)

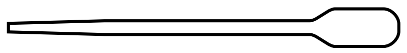

- ☐ Printer paper
  (Thermal paper, roll diameter: 2.2in / 55mm, roll length: approx. 112ft / 34m)
  * Please contact the agent from which you have purchased the product for details.

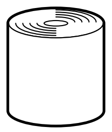

⚠Caution Make sure you place the Platform on a stable, level surface.If the Platform is not stable because not all the feet are on the surface, for example, there is a risk of stumbling or inaccurate measurement.

Control panel

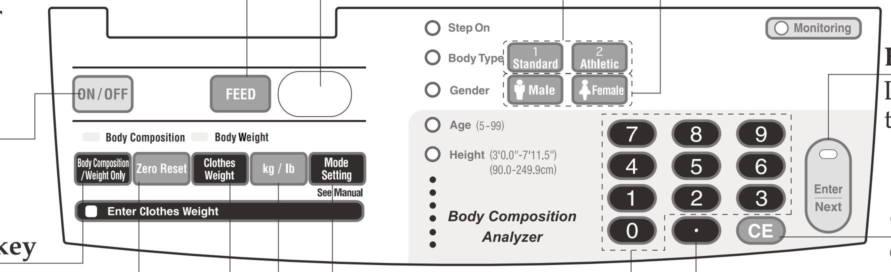

Connection of Plugs

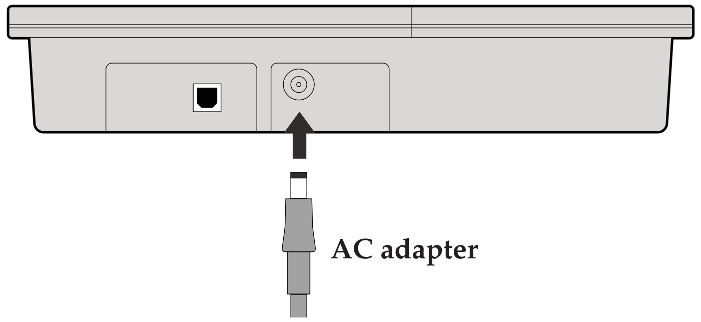

Warning

- **To avoid electric shock, do not insert or remove the plug with wet hands.**
- **To avoid electric shock, do not use the equipment near water.**

Caution

- To avoid measurement error, do not measure while using equipment that generates radio waves, such as cellular phone.
- Use only the original AC adapter. Using AC adapters other than the original one may cause malfunction, smoke or fire.

Caution

- Please change the printer paper when red lines appear along the sides of the paper.
- Please pay careful attention to avoid injury from the sharp edge.
- Please turn off the machine before clearing Paper jams.

Setting of the printer paper

1 Press ON/OFF to turn on the power.

- After all lamps light up, the model number is displayed as 331, and 0.0kg is displayed.

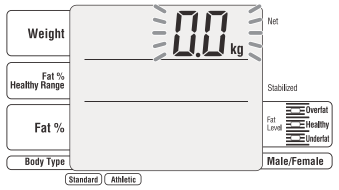

2 (1) Press OPEN.
(2) Remove the printer cover.

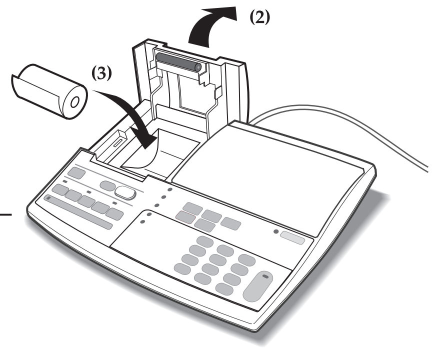

3 (3) Set up the printer paper

- Remove the adhesive of the printer paper and draw it out approx. 4in / 10cm.

4 Return the printer cover.

- In the case that C.OPEn is displayed,
  ⇨The printer cover is open, so please close it again properly (☞page 49).

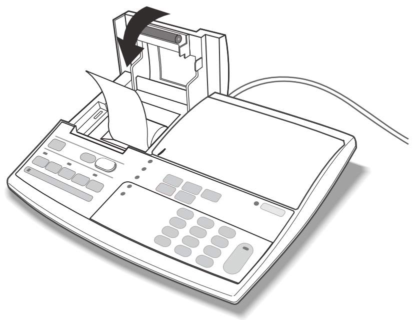

5 Press the FEED, and cut off the excessive paper.

- In the case that the automatic cutting is set to "OFF," the automatic cutting does not function (☞page 16).

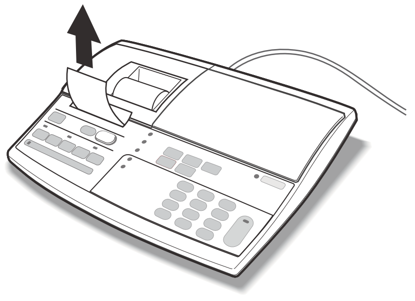

6 Setting completed.

Various settings (Setting methods)

Eng
Before use (Various settings)

Call up the setting item selection screen.

1 Press [ON/OFF] to turn on the power.

• After all lamps light up, the model number is displayed, and [0.0 kg] is displayed.

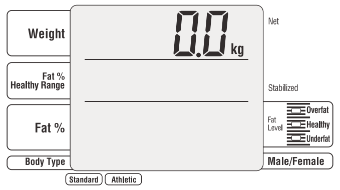

2 Press [Mode Setting]

• The setting item input screen is displayed.

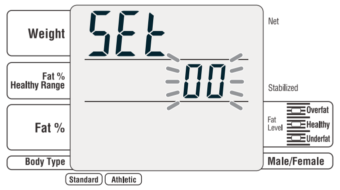

Note
- 39 – 64 are the setting items related to print out items (☞ page 24).
- When the various settings are all completed,
  ⇨Press [Mode Setting] on the "Setting item selection" screen (**it returns to the preset tare input screen**).

Setting items

1	Date and time(☞ page 14)
2	Number of sheets to print<br>Body composition analyzer (☞ page 16)
3	Number of sheets to print<br>Weight only mode (☞ page 15)
4	Auto Cutting of the printer paper (☞ page 16)
5	Beep sound (☞ page 16)
6	Display Fat % Healthy range (☞ page 17)
7	ID Number (☞ page 17)
8	Measurement flow (☞ page 18)
9	Athletic mode (☞ page 18)
10*	Height increment (☞ page 19)
11	Automatic determination time (☞ page 19)
17	Wrestler mode (☞ page 20)
18	Target body fat % (☞ page 21)
19	Select language (☞ page 21)
20	Print item preset (☞ page 22)

* Invalid in "lb" mode.

Print item setting ON / OFF (☞ page 24)

39	TANITA Logo
40	Category name
41	Date and Time
42	Serial number
43	Memo space
44	ID Number
45	Fat mass
46	Fat free mass
47	Muscle mass
48	Total body water
49	Total body water %
50	Bone mass
51	Basal metabolic rate
52	Metabolic age
53	Visceral fat rating
54	BMI
56	Ideal body weight
57	Degree of obesity
58	Desirable range Body fat
59	Indicator - Fat %
60	Indicator - BMI
61	Indicator - Visceral fat rating
62	Indicator - Muscle mass
63	Indicator - BMR
64	Physique rating

Note
*When various settings are continuously carried out,
⇨press each number to set.
*The set contents are memorized until they are changed next time.

*When various settings are all completed,
⇨press [Mode Setting] on the "setting item selection screen" (**it returns to the preset tare input screen**).

Various settings Setting methods (continued)

Register the date and time (Continued from page 12).

3 Press 1 and then press Enter / Next.

• The "date and time" setting screen is displayed.

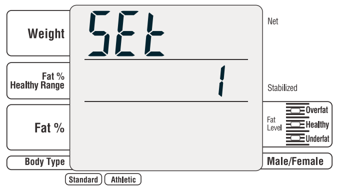

4 Input the date and time

• Input the year, month, day, hour and minute in order with 2 digits.

Example: In the case to input: 9:47 am January 21st, 2009

0 9 0 1 2 1 0 9 4 7

Year 2009 January 21st

In the case of 6:00 pm, press 1 8

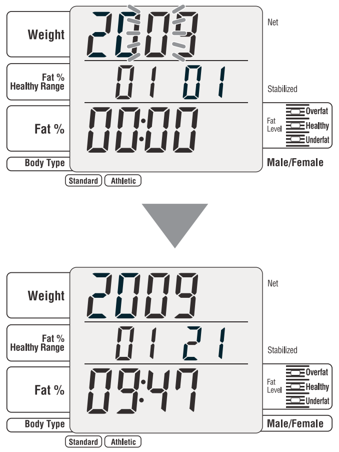

Note
• To input a number with 1 digit (0 – 9), first press 0.
• To correct the input value, or cancel the input,
⇨press CE (the input is deleted).
• To end inputting in midstream,
⇨press Enter / Next.

5 After inputting all the items, press Enter / Next.

• It returns to the "setting item selection" screen.

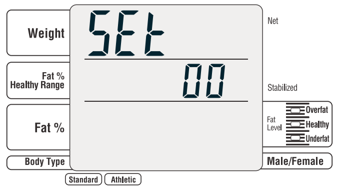

Note *When various settings are continuously carried out,
⇨press each number to set.
*The set contents are memorized until they are changed next time.

Set the number of sheets to print for the body composition Analyzer (Continued from page 12).

3 Press 2 and then press Enter / Next.

• The "number of sheets to print for the body composition analyzer" setting screen is displayed.

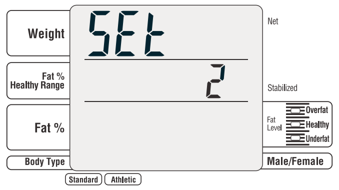

4 Input the number of sheets to print.

Note
• The default is "1" (input range: 0 – 3).
• In the case that the number of sheets to print is set to "0" for both the body composition analyzer and the scale, FEED key does not function.
• To correct the input value, or cancel the input,
⇨press CE (the input is deleted).

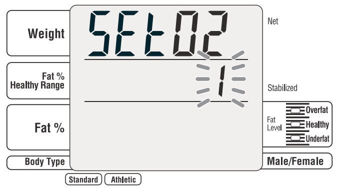

5 After inputting the numeric value, press Enter / Next.

• It returns to the "setting item selection" screen.

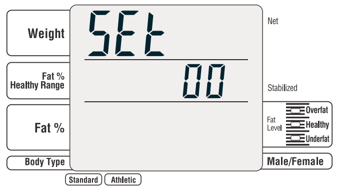

Set the number of sheets to print for the scale (Continued from page 12).

3 Press 3 and then press Enter / Next.

• The "number of sheets to print for the scale" setting screen is displayed.

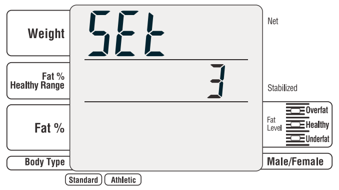

4 Input the number of sheets to print.

Note
• The default is "1" (input range: 0 – 3).
• In the case that the number of sheets to print is set to "0" for both the body composition analyzer and the scale, FEED key does not function.
• To correct the input value, or cancel the input,
⇨press CE (the input is deleted).

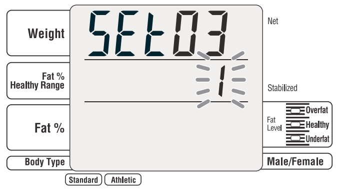

5 After inputting the numeric value, press Enter / Next.

• It returns to the "setting item selection" screen.

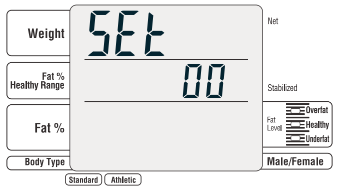

*When various settings are all completed,
⇨press Mode Setting on the "setting item selection screen" (**it returns to the preset tare input screen**).

Set ON / OFF of the automatic cutting of the printer paper (Continued from page 12).

3 **Press 4 and then press Enter / Next.**

• The “ON or OFF of automatic cutting” selection screen is displayed.

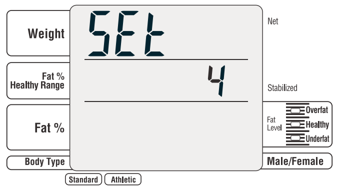

4 **Set ON or OFF of the automatic cutting.**

Note • The default is “0.off.” (“1. on” for valid, “0. off” for invalid.)
• To correct the input value, or cancel the input,
⇨press CE (the input is deleted).

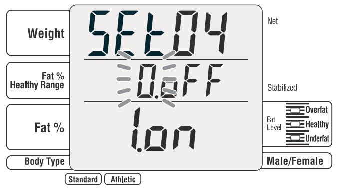

5 **After inputting the numeric value, press Enter / Next.**

• It returns to the “setting item selection” screen.

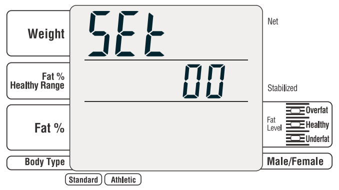

Set ON / OFF of the beep sound (Continued from page 12).

3 **Press 5 and then press Enter / Next.**

• The “ON or OFF of the beep sound” selection screen is displayed.

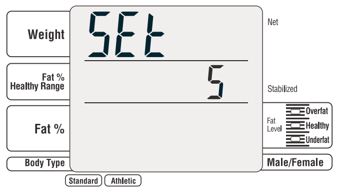

4 **Set ON or OFF of the beep sound.**

Note • The default is “1.on.” (“1. on” for valid, “0. off” for invalid.)
• To correct the input value, or cancel the input,
⇨press CE (the input is deleted).

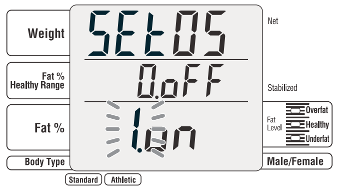

5 **After inputting the numeric value, press Enter / Next.**

• It returns to the “setting item selection” screen.

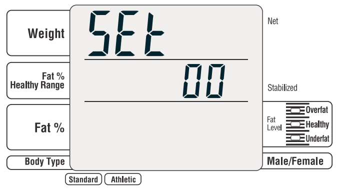

Note * When various settings are continuously carried out,
⇨press each number to set.
* The set contents are memorized until they are changed next time.

Set ON / OFF of the Fat % Healthy range display (Continued from page 12).

3 **Press 6 and then press Enter / Next.**

• The “ON or OFF of the Fat % Healthy range” selection screen is displayed.

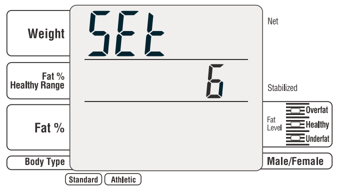

4 **Set ON or OFF of the Fat % Healthy range display when using the body composition**

Note • The default is “1.on.” (“1. on” for valid, “0. off” for invalid.)
• To correct the input value, or cancel the input,
⇨press CE (the input is deleted).

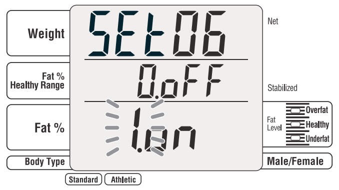

5 **After inputting the numeric value, press Enter / Next.**

• It returns to the “setting item selection” screen.

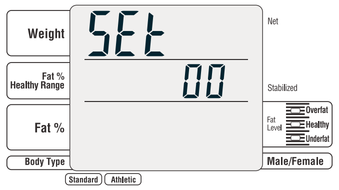

Set with or without an ID (Continued from page 12).

3 **Press 7 and then press Enter / Next.**

• The “with or without an ID” setting screen is displayed.

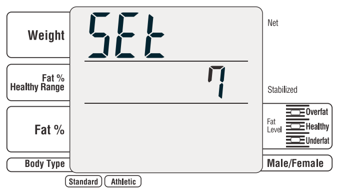

4 **Set with or without an ID.**

Note • The default is “0.off.” (“1. on” for valid, “0. off” for invalid.)
• To correct the input value, or cancel the input,
⇨press CE (the input is deleted).

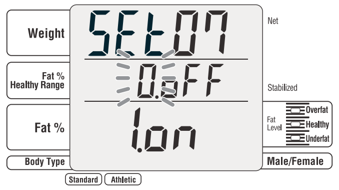

5 **After inputting the numeric value, press Enter / Next.**

• It returns to the “setting item selection” screen.

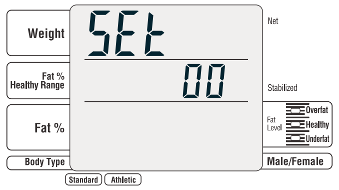

* When various settings are all completed,
⇨press Mode Setting on the “setting item selection screen” (**it returns to the preset tare input screen**).

Various settings (Setting methods (continued))

Select the measurement flow (Continued from page 12).

3 **Press 8 and then press Enter / Next.**

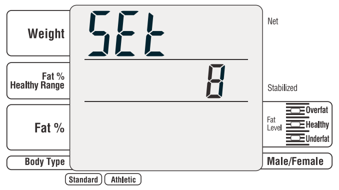

- The "ON or OFF of the one step mode" selection screen is displayed.

Note
- The one step mode is
  ⇨a mode to measure body weight after inputting personal information.

4 **Set ON or OFF of the one step mode when using the body composition analyzer.**

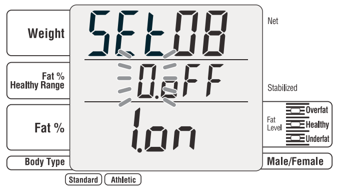

Note
- The default is "0. off." ("1. ON" for valid, "0. off" for invalid.)
- To correct the input value, or cancel the input,
  ⇨press CE (the input is deleted).

5 **After inputting the numeric value, press Enter / Next.**

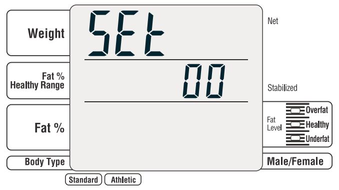

- It returns to the "setting item selection" screen.

Set ON or OFF of the athletic mode selection (Continued from page 12).

3 **Press 9 and then press Enter / Next.**

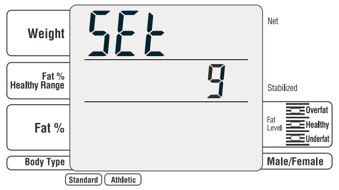

- The "Set ON / OFF of the athletic mode selection" setting screen is displayed.

4 **Set ON or OFF of the athletic mode selection when using the body composition analyzer.**

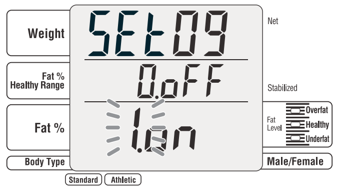

Note
- The default is "1. on." ("1. on" for valid, "0. off" for invalid.)
- To correct the input value, or cancel the input,
  ⇨press CE (the input is deleted).

5 **After inputting the numeric value, press Enter / Next.**

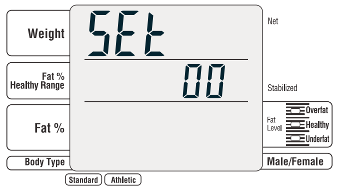

- It returns to the "setting item selection" screen.

Note
*When various settings are continuously carried out,
⇨press each number to set.
*The set contents are memorized until they are changed next time.

Set the height increment (Continued from page 12).

3 **Press 1 0 and then press Enter / Next.**

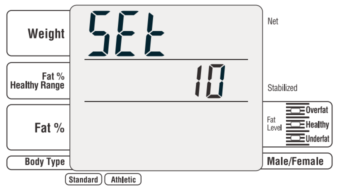

- The "height increment" selection screen is displayed.

* Invalid in "lb" mode.

4 **Selects the height incremant.**

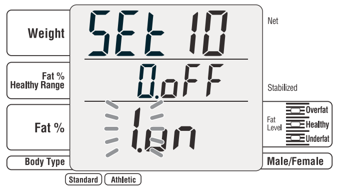

Note
- The default is "1. on" (1 cm / 0.5 in).
  0. off: sets 0.1 cm increment
  1. on: sets 1 cm / 0.5 in increment
- To correct the input value, or cancel the input,
  ⇨press CE (the input is deleted).

5 **After inputting the numeric value, press Enter / Next.**

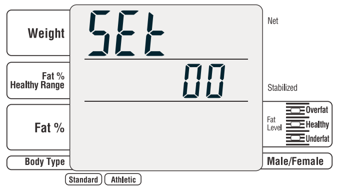

- It returns to the "setting item selection" screen.

Set the automatic determination time when inputting (Continued from page 12).

3 **Press 1 1 and then press Enter / Next.**

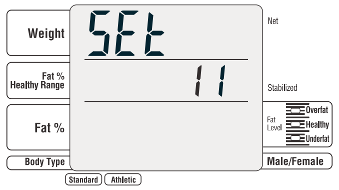

- The "automatic determination time when inputting" setting screen is displayed.

Note
- Automatic determination when inputting is,
  ⇨a function to determine the input value automatically even without pressing Enter / Next, after inputting the numeric value.

4 **Set the automatic determination time when inputting.**

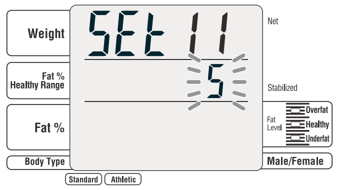

Note
- The default is 5 seconds ("5"). (input range: 0 – 9).
  *If "0" is set, it is not automatically determined.
- To correct the input value, or cancel the input,
  ⇨press CE (the input is deleted).

5 **After inputting the numeric value, press Enter / Next.**

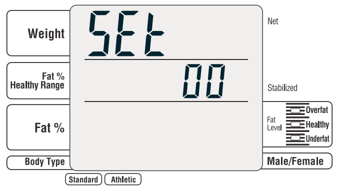

- It returns to the "setting item selection" screen.

*When various settings are all completed,
⇨press Mode Setting on the "setting item selection screen" (**it returns to the preset tare input screen**).

Various settings Setting methods (continued)

Set ON / OFF of Wrestler mode (Continued from page 12).

3 Press 1 7 and then press Enter / Next.

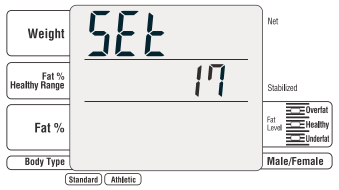

4 Set ON or OFF of Wrestler mode.

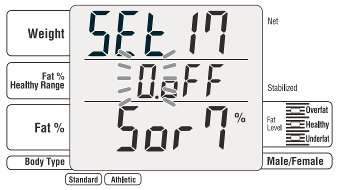

Note
- The default is 0.off
- 0: Set Wrestler mode off
- 5: Set Wrestler mode 5%
- 7: Set Wrestler mode 7%
- When the "Wrestler Mode" is activated, the SC-331S automatically calculates the Minimum Wrestling Weight at a predetermined minimum body fat %. The 1996 ACSM Position Stand "Weight Loss in Wrestlers" as adopted by the NCAA recommends the following MINIMUM body fat % as follows:
  - 5% for Collegiate Athletes (male)
  - 7% for High School Athletes (male)

⚠ The target body fat % value is completely separate from the Minimum Wrestling Weight (Min Weight) calculations.
For example:
The target body fat % value may be entered as 15%, even though the Min Weight is calculated at a predetermined minimum body fat of 5 or 7%.

5 After inputting the numeric value, press Enter / Next.

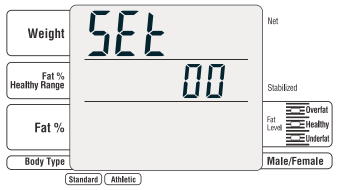

- It returns to the "setting item selection" screen.

Set ON / OFF of the target body fat % (Continued from page 12).

3 Press 1 8 and then press Enter / Next.

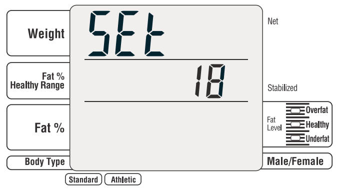

4 Set ON or OFF of the target body fat %.

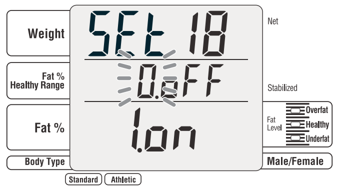

Note
- The default is "0. off" ("1. on" for valid, "0. off" for invalid.)
- To correct the input value, or cancel the input,
  ⇨press CE (the input is deleted).

5 After inputting the numeric value, press Enter / Next.

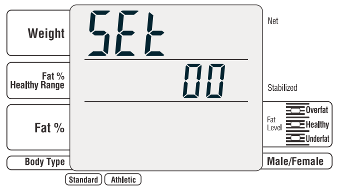

- It returns to the "setting item selection" screen.

Set print language (Continued from page 12).

3 Press 1 9 and then press Enter / Next.

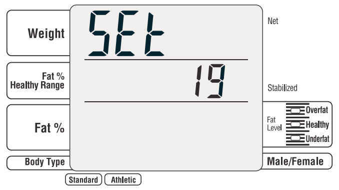

4 Set the print language.

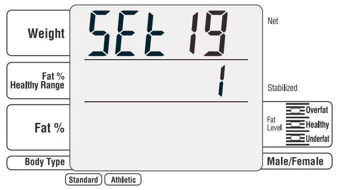

Note
- 1: English / 2: French / 5: Spanish / 7: Portuguese
- To correct the input value, or cancel the input,
  ⇨press CE (the input is deleted).

5 After inputting the numeric value, press Enter / Next.

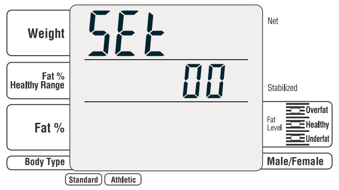

- It returns to the "setting item selection" screen.

Note
*When various settings are continuously carried out,
⇨press each number to set.
*The set contents are memorized until they are changed next time.

*When various settings are all completed,
⇨press Set Up on the "setting item selection screen" (**it returns to the preset tare input screen**).

Set the print item preset (Continued from page 12).

3 Press 2 0 and then press Enter / Next.

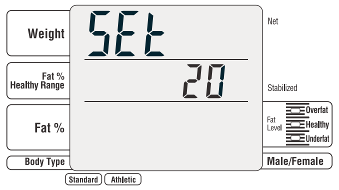

• The "print out preset" setting screen is displayed.

4 Set the print item preset.

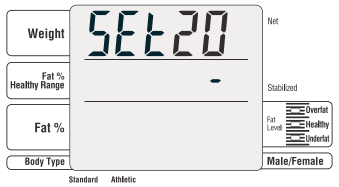

• Set with 1 – 3.

Note
• The print items preset are
⇨a function to set the print items of the pattern 1 – 3 to ON automatically at once (☞page 23). The default is "1" (pattern 1).
• When ON or OFF of print items are changed with the "setting of the print out item" after the print out preset (☞page 26), the state set for the last time is valid.

5 After inputting the numeric value, press Enter / Next.

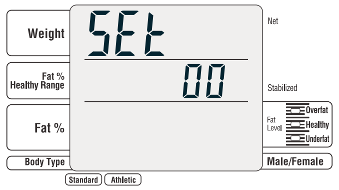

• It returns to the "setting item selection" screen.

Note
*When various settings are continuously carried out,
⇨press each number to set.
*The set contents are memorized until they are changed next time.

[Lists of contents of the print item preset]

Print item	Body composition analyzer									Scale
	1 Pattern 1			2 Pattern 2			3 Pattern 3			
Body type	Standard	Athletic	Child	Standard	Athletic	Child	Standard	Athletic	Child	
TANITA Logo	✓	✓	✓	✓	✓	✓	✓	✓	✓	
Category name	✓	✓	✓	✓	✓	✓	✓	✓	✓	✓
Date and time	✓	✓	✓	✓	✓	✓	✓	✓	✓	✓
Serial number	✓	✓	✓	✓	✓	✓	✓	✓	✓	✓
Memo Space	✓	✓	✓	✓	✓	✓	✓	✓	✓	
ID No	✓	✓	✓	✓	✓	✓	✓	✓	✓	✓
Fat mass	✓	✓	✓	✓	✓	✓				
Fat free mass	✓	✓	✓	✓	✓	✓				
Muscle mass	✓	✓		✓	✓					
Total body water	✓	✓		✓	✓					
Total body water %	✓	✓		✓	✓					
Bone mass	✓	✓		✓	✓					
BMR	✓	✓		✓	✓					
Metabolic age	✓	✓		✓	✓					
Visceral fat rating	✓	✓		✓	✓					
BMI	✓	✓	✓	✓	✓	✓	✓	✓	✓	
Ideal body weight	✓			✓						
Degree of obesity	✓			✓						
Desirable range	✓	✓	✓							
Wrestler Mode										
Indicator Fat %	✓	✓	✓	✓	✓	✓				
Indicator BMI	✓	✓		✓	✓					
Indicator Visceral fat rating	✓	✓								
Indicator Muscle mass	✓	✓								
Indicator BMR	✓	✓								
Physique rating	✓	✓								

• The items marked with "✓" are printed.
• The items marked with "✓" can be selected to print (☞page 24)
* See page 24 for an example of preset print.

*When various settings are all completed,
⇨press Mode Setting on the "setting item selection screen" (**it returns to the preset tare input screen**).

[In the case to select the print item preset "1"]

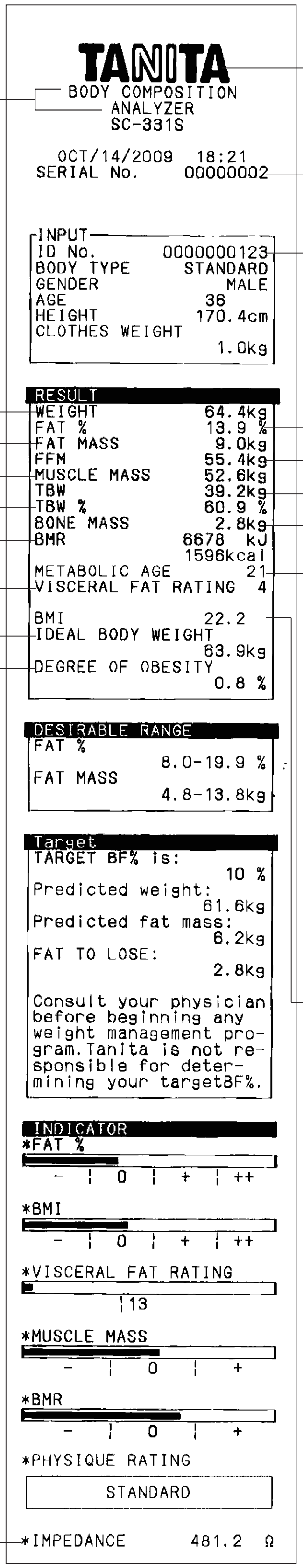

Category name

Weight
• Measured weight.

Fat mass
• -Total weight of fat mass in the body.

Muscle mass*
• - Bone-free lean tissue mass (LTM)

TBW %*

BMR*
• Basal Metabolic Rate represents the total energy expended by the body to maintain normal functions at rest such as respiration and circulation.

Visceral fat rating*
• - Visceral fat rating feature indicates the rating of visceral fat.

Ideal body weight*
• Ideal body weight is a value for which the BMI is 22.

Degree of obesity*
• Calculated as (weight – Ideal body weight) / Ideal body weight × 100.

Impedance
• Impedance (This does not affect judgment of the measurement results.)

LOGO

Serial No.
• The default is 00000001.
Adds 1 each time it measures.

ID
• When it is set with an ID, it is printed out. (The default is without an ID.)

Fat %
• Fat % is amount of body fat as a proportion of body weight.

FFM
• Fat Free Mass is comprised of muscle, bone, tissue, water, and all other fat free mass in the body.

TBW*
• Total Body Water is the amount of water retained in the body. TBW is said to comprise between 50% - 70% of total body weight. Generally, men tend to have higher water weight than women due to a greater amount of muscle.

Bone mass*
• Bone mineral amount included in the entire bone.

Metabolic age*
• Metabolic age is evaluated young when a muscular amount is larger, and BMR is higher.

BMI
• Calculated with "weight (kg) / height (m)^2"
• The standard value is for the Standard mode. In the case of the Athletic mode, the standard value is just a reference. And for those who are 17 years old or younger, only the body fat % is displayed as the standard value. The muscle mass, total body water and the estimated bone mass for those who are 17 years old or younger are for reference.

***18 - 99 years only**

⚠ Please consult your doctor before you start a body weight management program. Tanita is not responsible for the target body fat ratio.

[In the case to select the print item preset "2"]

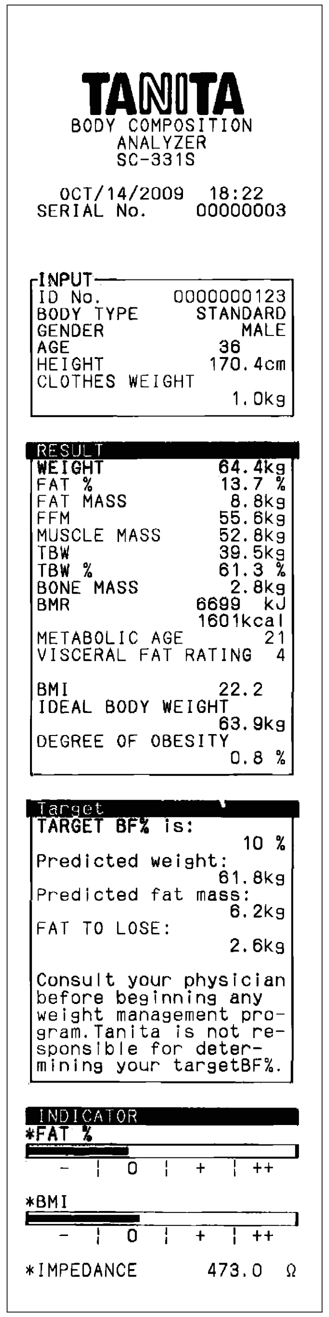

[In the case to select the print item preset "3"]

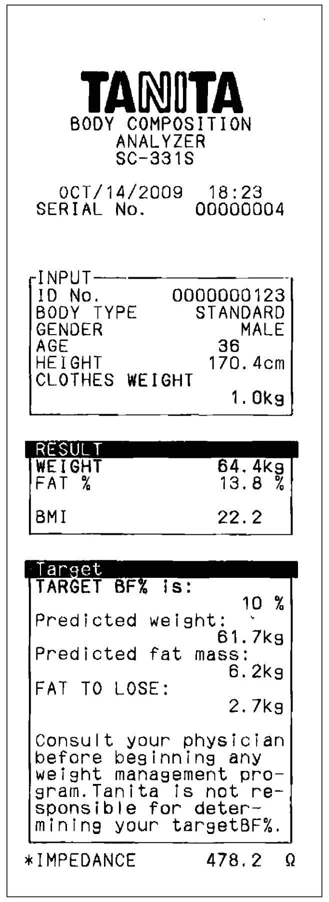

⚠ Please consult your doctor before you start a body weight management program. Tanita is not responsible for the target body fat ratio.

Set the items to print out (Continued from page 12).

3 Select the number to set with the numeric keys (3 9 – 6 4) and press Enter / Next.

<In the case of selecting 39>

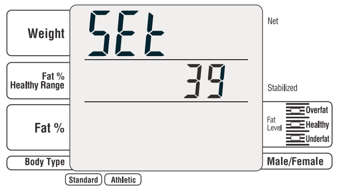

• The setting screen is displayed.

Note

• See the next page for the setting number of each item.
• When the print item preset is set, after setting the items to print out (☞page 24), the items to print may be changed automatically. Please confirm, "Print items preset list" (☞page 23).

Print item setting ON / OFF

39	TANITA Logo
40	Category name
41	Date and Time
42	Serial number
43	Memo space
44	ID Number
45	Fat mass
46	Fat free mass
47	Muscle mass
48	Total body water
49	Total body water %
50	Bone mass
51	Basal metabolic rate (BMR)

52	Metabolic age
53	Visceral fat rating
54	BMI
56	Ideal body weight
57	Degree of obesity
58	Desirable range Body fat %
59	Indicator - Fat %
60	Indicator - BMI
61	Indicator - Visceral fat rating
62	Indicator - Muscle mass
63	Indicator - BMR
64	Physique rating

4 Selects ON or OFF of the item to print out.

⟨ In the case of selecting the logo setting on. ⟩

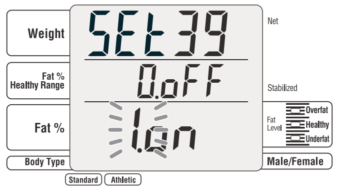

Note

• "0. off" is not to print out and "1. on" is to print out.
• To correct the input value, or cancel the input,
⇨press CE (the input is deleted).

5 After inputting the numeric value, press Enter / Next.

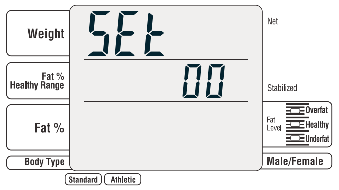

• It returns to the "setting item selection" screen.

Note

*When various settings are continuously carried out,
⇨press each number to set.
*The set contents are memorized until they are changed next time.

*When various settings are all completed,
⇨press Mode Setting on the "setting item selection screen" (**it returns to the preset tare input screen**).

This explains the procedure when the printer is turned on. Please be aware that the display may be different if the number of sheets to print is set to 0.

- Do not wipe the equipment with corrosive chemicals (gasoline, cleaner, etc.). Please use a neutral detergent to clean the equipment.
- When the equipment has been transferred to any location where there is a temperature difference of 36˚F / 20˚C or more, wait for at least two hours before using it.
- In taking measurements, please keep the person away from the unit, who uses transmitters such as a cellular phone avoid causing margin errors.
- The SC-331S estimates the various outputs specified in the Specification Section of this Manual. Keep in mind that there are many factors that can influence the accuracy of these measurements. Please refer to the General Instructions Section of this manual as this will help you minimize the variability (inaccuracy) of the measurements.

About Athletic Mode

- **It is recommended for those who are 18 years old or older and meet the following conditions to select ”Athletic Mode” and measure as reference values.**
- Those who exercise for 12 hours or more per a week.
- Those who belong to a sport team or a sport organization with the aim of participation in competition, etc.
- Those who exercise to build up like a bodybuilder.
- Those who are professional athletes.

Attention

- **The posture when measuring**
- Stand with both feet parallel on the electrodes.
- Stand fullface without bending knees.
- **The age input range is 5 – 99 years old.**
Input age 99 for those who are 100 years old or older.

Note

- False results may be reported after excessive food/fluid intake, or after periods of intense exercise.
- When the user is 18 years or older, Athletic mode can be used.
- When Clothes weight is input, Clothes weight is subtracted from measurements and it displays it as Weight.
- **“One step mode”should be the default.**
- **“Option mode”should move to back of “One step mode” page.**

In the case of the “one step mode”

In the one step mode, after inputting the personal data, the weight and body composition are measured.

Personal data input ▶ **Weight measurement** ▶ **Body composition measurement**

1 Press ON/OFF to turn on the power.

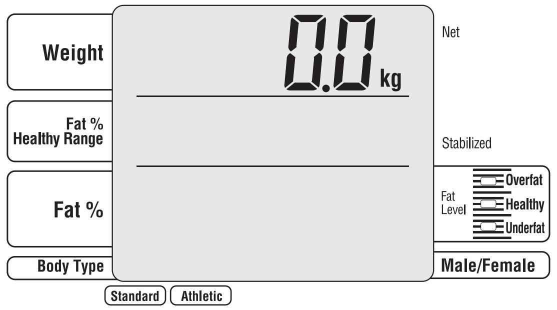

(kg mode)

2 Check that the body composition is selected, input weight of closthing.

Input it by pressing 0 – 9 and ·.

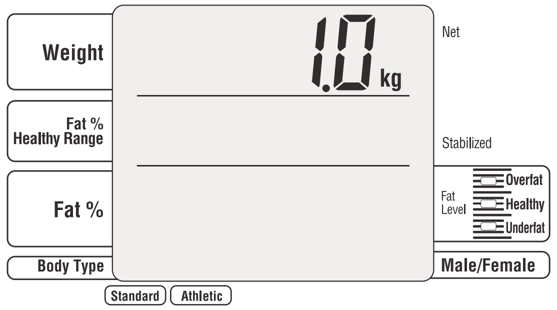

Note

- The clothes weight (preset tare) can be input in the range of 0.0-20.0lb or 0.0-10.0kg.
*0.2lb / 0.1kg increments
*Unit is changed by kg / lb key.
- If it is mistakenly input,
⇨press CE (the input is deleted).

3 Press Enter / Next.

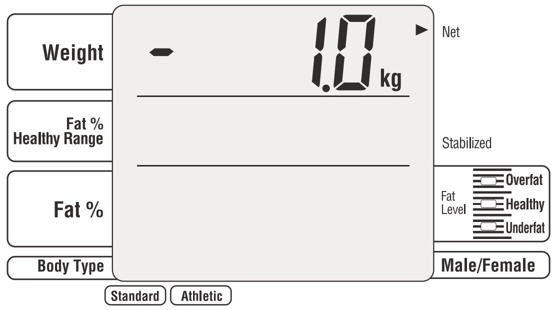

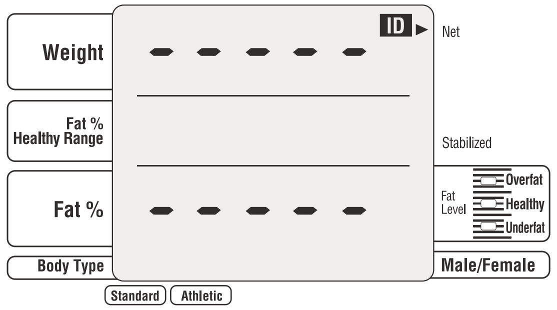

Note

- **This screen is not displayed if OFF is set in the “setting with or without an ID” (☞page 17). (The “body type selection” screen is displayed.)**

4 Input ID number.

Input it by pressing 0 – 9.

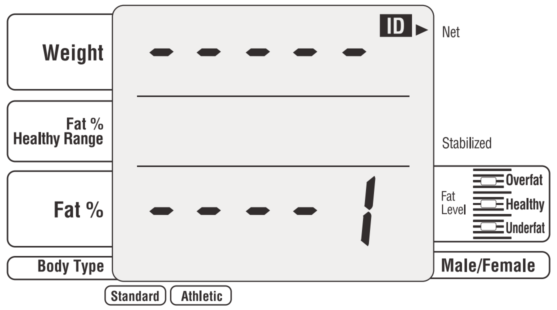

Note

- **This screen is not displayed if OFF is set in the “setting with or without an ID” (☞page 17).**
- The ID number can be input from 0 – 9999999999. If Enter / Next is pressed, the non-inputted digits are filled with 0s.
- If it is mistakenly input,
⇨press CE (the input is deleted).
- If CE is pressed in the state that an ID number is not input, it returns to the “measurement start” screen.

5 Select body type.

Press the body type selection keys to input.
When the body type is selected, the lamp flashes on the "Gender".

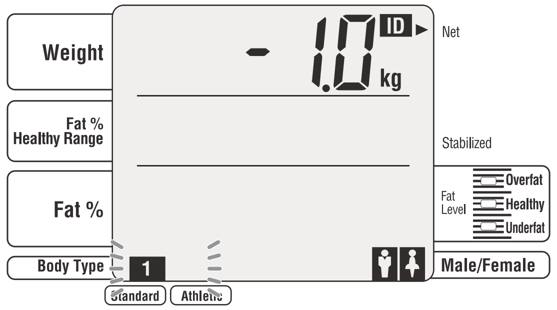

Note

- **This screen is not displayed if OFF is set in the "setting ON or OFF of the athletic mode selection" (☞ page 18).**
- The body type can also be selected with the numeric keys (1 2).
- If it is mistakenly input,
  ⇨press CE (the input is deleted, and it returns to the "body type selection" screen).
- If CE is pressed in the state that the body type is not input, it returns to the "ID number input" screen (or "measurement start" screen).

6 Select gender.

Press the male / female selection keys to input.
When male or female is selected, the lamp flashes on the "Age".

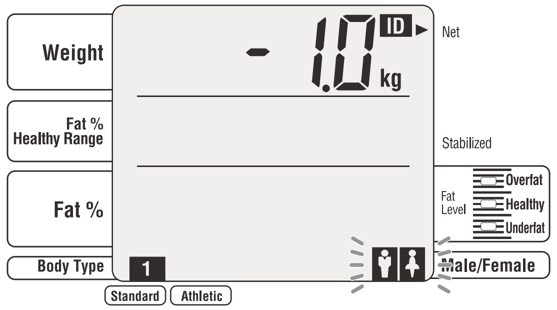

Note

- If it is mistakenly input,
  ⇨press CE (the input is deleted, and it returns to the "gender selection" screen).
- If CE is pressed in the state that the male or female is not selected, it returns to the "body type selection" screen.

7 Input age.

Input it by pressing 0 – 9 .

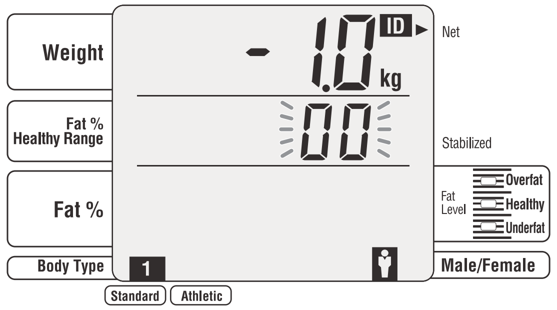

Note

- The age can be input from 5 – 99.
- If it is mistakenly input,
  ⇨press CE (the input is deleted).
- If CE is pressed in the state that the age is not input, it returns to the "gender selection" screen.

8 Press Enter / Next .

When the age is input, the lamp flashes on the "Height".

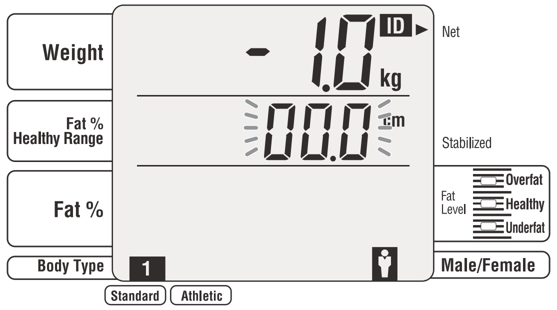

9 Input height.

Input it by pressing 0 – 9 .

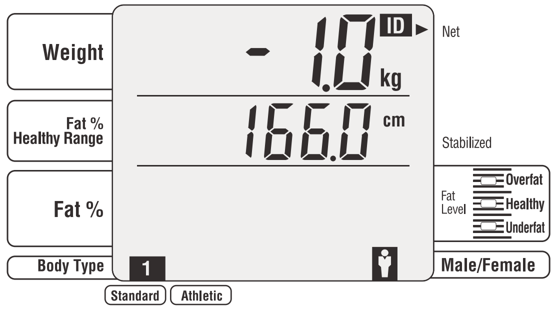

Note

- The height can be input from 3ft -7ft11.5in / 90.0-249.9cm.
  *Unit is changed by kg / lb key.
- If it is mistakenly input,
  ⇨press CE (the input is deleted).
- If CE is pressed in the state that the height is not input, it returns to the "age input" screen.

10 Press Enter / Next .

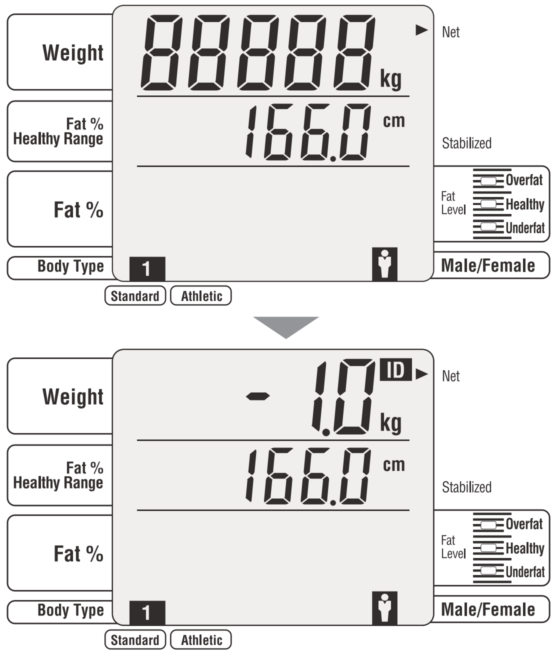

The "Step on" lamp flashes.

Note

- When CE is pressed, it returns to the previous screen.

11 Step on the electrodes with bare feet.

Take off your socks and stockings before stepping on.

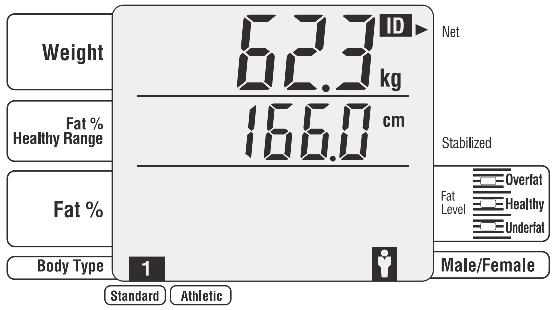

12 Under measurement of the body composition.

88888 display goes off sequentially.

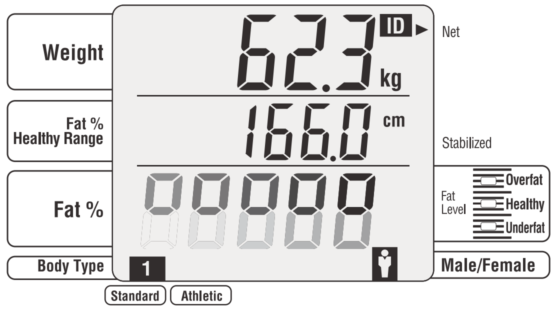

13 Measurement completion

The measurement result and the body fat percentage evaluation are displayed.
It is automatically printed out. (In the case that other than 0 is set in the "Setting the number of sheets to print" (☞ page 15).

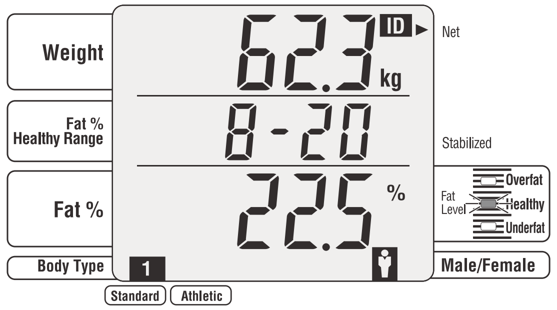

Note

- **Fat % Healthy range is not displayed if OFF is set in the "setting ON or OFF of the Fat % Healthy range display" (☞ page 17).**

Step off the platform.
It returns to the "ID input" screen.

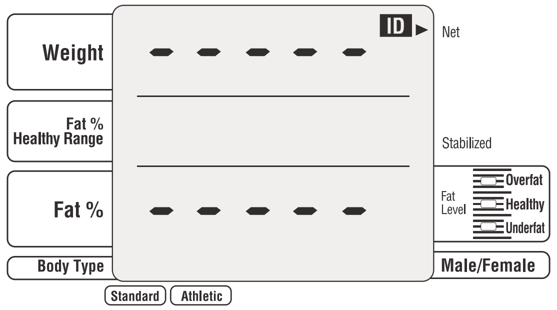

Note

- **The "body type selection" screen is displayed in the case that OFF is set in the "setting with or without an ID" (☞ page 17).**

How to read the display <kg mode>

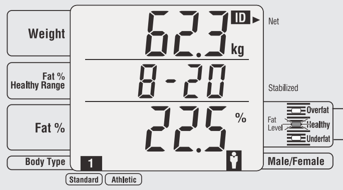

Judgment result based on the body fat percentage. (☞ page 42.)

How to read the display <lb mode>

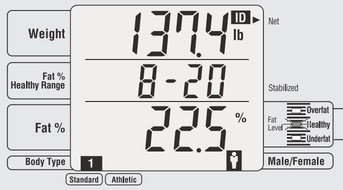

Judgment result based on the body fat percentage. (☞ page 42.)

In the case of the "Option mode" (not the one step mode)

In the option mode, after measuring weight, personal data is input and then body composition is measured.

Weight measurement ▶ Personal data input ▶ Body composition measurement

1 Press ON/OFF to turn on the power.

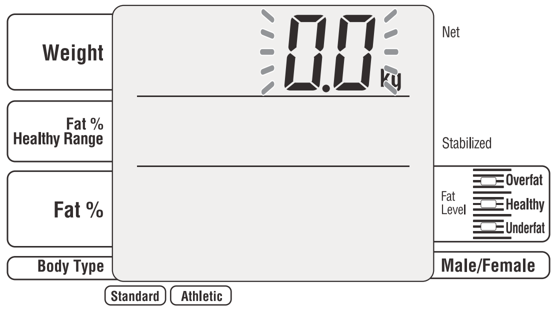

(kg mode)

Note

- Unit is changed by kg / lb key.

2 Check that the body composition is selected and input weight of clothing.

Input it by pressing 0 – 9 and ..

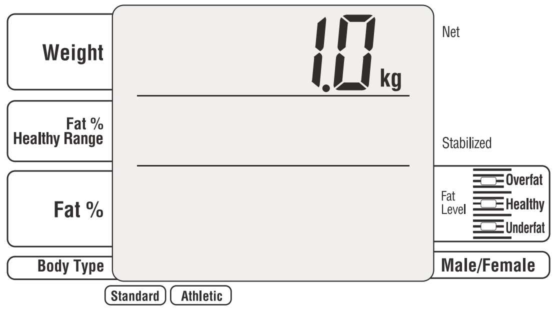

Note

- The clothes weight (preset tare) can be input in the range of 0.0-20.0lb or 0.0-10.0kg.
  *0.2lb / 0.1kg increments
- To correct the input value,
  ⇨press CE (the input is deleted).

3 Press Enter / Next.

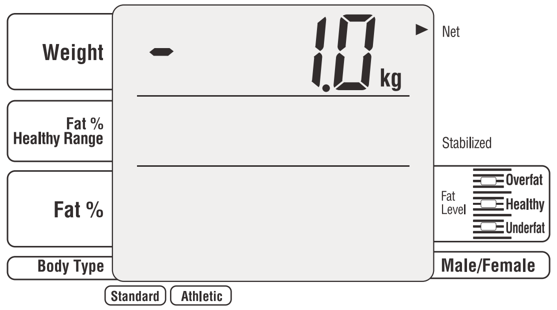

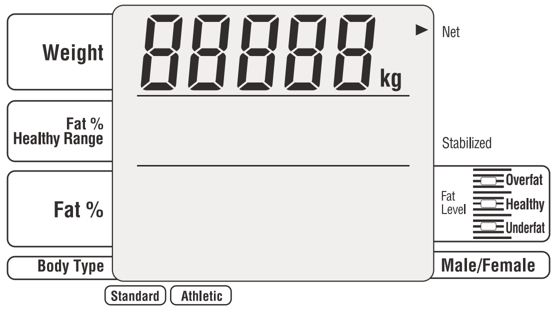

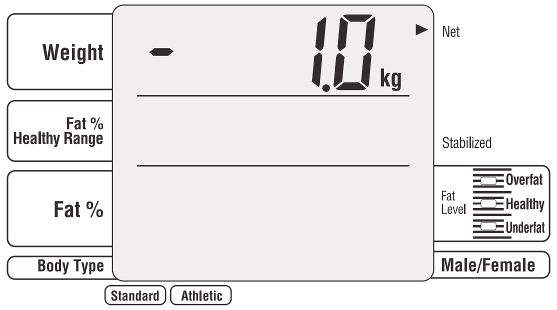

The "Step on" lamp flashes.

Note

- When CE is pressed, it returns to the previous screen.

4 Step on the electrodes with bare feet.

Take off your socks and stockings before stepping on.

<Child> <Adult>

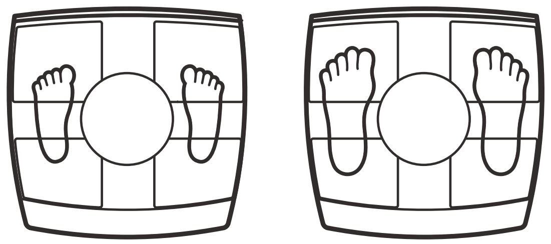

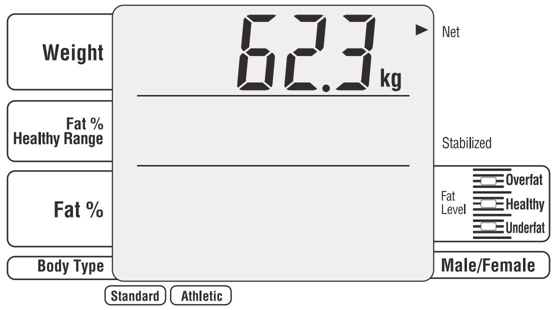

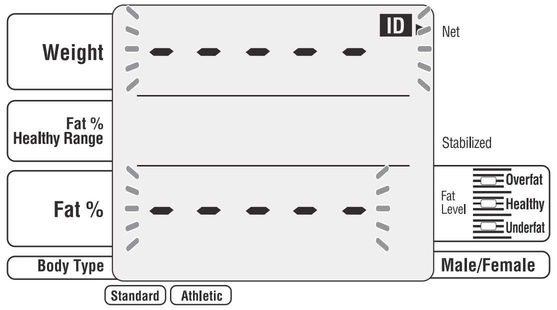

When the weight becomes stable, it changes to the screen at the right.

Note

- Do not step off the platform.
- **This screen is not displayed if OFF is set in the “setting with or without an ID” (☞ page 17). (The “body type selection” screen is displayed.)**

5 Input ID number.

Input it by pressing 0 – 9.

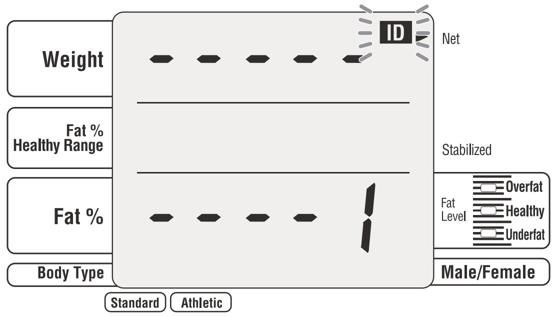

Note

- **This screen is not displayed if OFF is set in the “setting with or without an ID” (☞ page 17).**
- The ID number can be input from 0 – 9999999999. If Enter / Next is pressed, the non-inputted digits are filled with 0s.
- If it is mistakenly input,
  ⇨press CE (the input is deleted).
- If CE is pressed in the state that an ID number is not input, it returns to the “measurement start” screen.

6 Press Enter / Next.

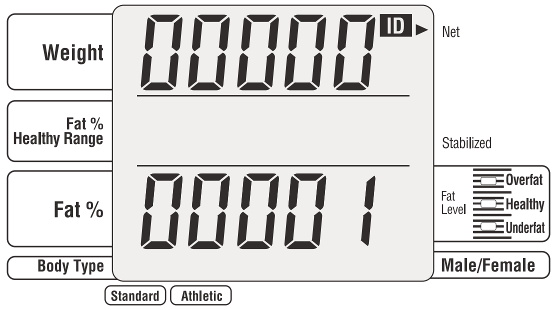

Note

- **This screen is not displayed if OFF is set in the “setting with or without an ID” (☞ page 17).**

7 Select body type.

Press the body type selection keys to input.
When the body type is selected, the lamp flashes on the “Gender”.

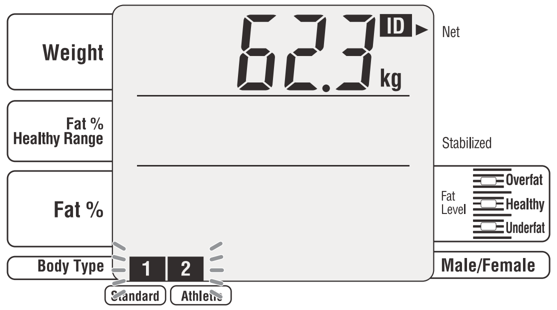

Note

- **This screen is not displayed if OFF is set in the “setting ON or OFF of the athletic mode selection” (☞ page 18).**
- The body type can also be selected with the numeric keys (1 2).
- If it is mistakenly input,
  ⇨press CE (the input is deleted, and it returns to the “body type selection” screen).
- If CE is pressed in the state that the body type is not input, it returns to the “ID number input” screen (or “measurement start” screen).

8 Select gender.

Press the male / female selection keys to input.
When male or female is selected, the lamp flashes on the “Age”.

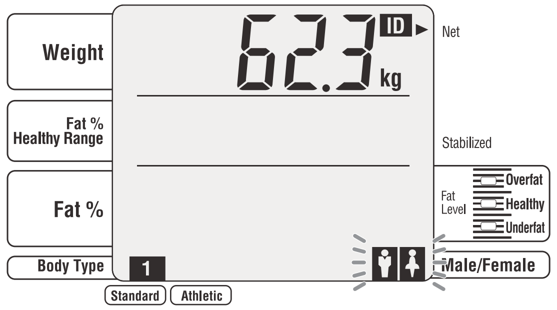

Note

- If it is mistakenly input,
  ⇨press CE (the input is deleted, and it returns to the “gender selection” screen).
- If CE is pressed in the state that the male or female is not selected, it returns to the “body type selection” screen.

9 Input age.

Input it by pressing 0 – 9.

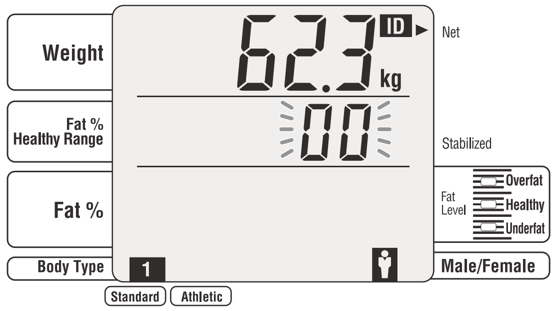

Note

- The age can be input from 5 – 99.
- If it is mistakenly input,
  ⇨press CE (the input is deleted).
- If CE is pressed in the state that the age is not input, it returns to the “gender selection” screen.

10 Press Enter / Next.

When the age is input, the lamp flashes on the "Height".

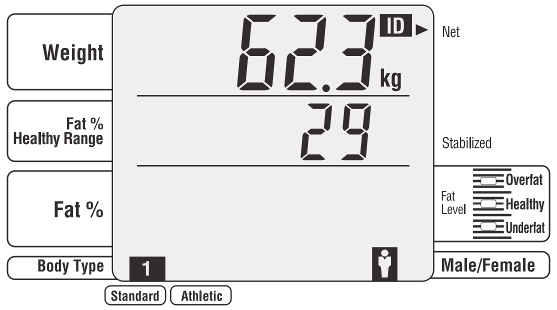

11 Input height.

Input it by pressing 0 – 9.

Note

- The height can be input from 3ft -7ft11.5in / 90.0-249.9cm.

  *Unit is changed by kg / lb key.
- If it is mistakenly input,

  ⇨press CE (the input is deleted).
- If CE is pressed in the state that the height is not input, it returns to the "age input" screen.

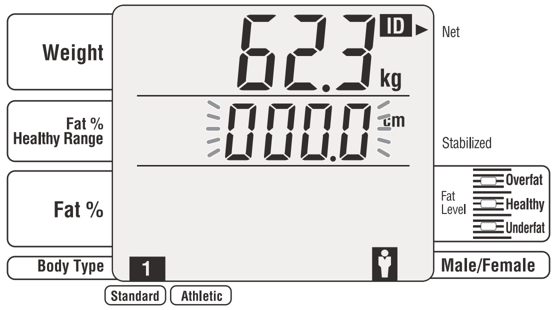

12 Press Enter / Next.

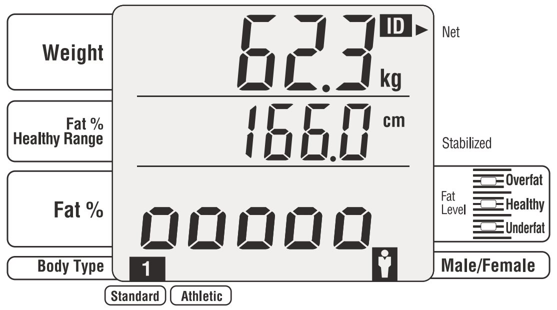

13 Under measurement of the body composition.

88888 display goes off sequentially.

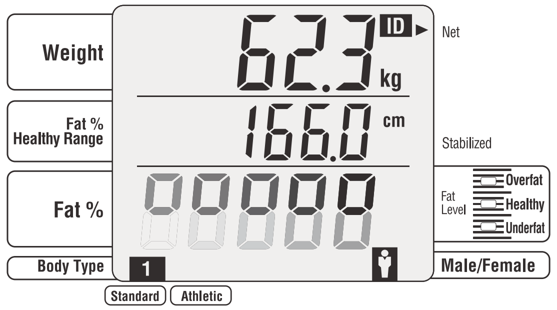

14 Measurement completion

The measurement result and the body fat percentage evaluation are displayed.

It is automatically printed out. (In the case that other than 0 is set in the "Setting the number of sheets to print" (☞page 15).

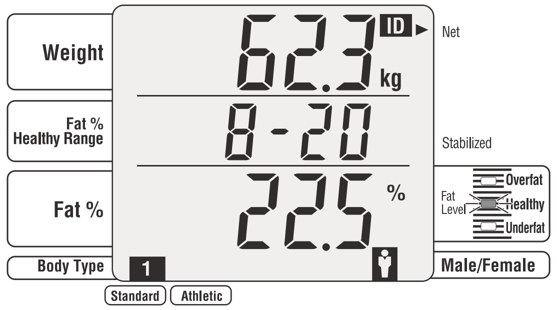

Note

- **Fat % Healthy range is not displayed if OFF is set in the "setting ON or OFF of the Fat % Healthy range display" (☞page 17).**

Step off the platform.

It returns to the "measurement start" screen.

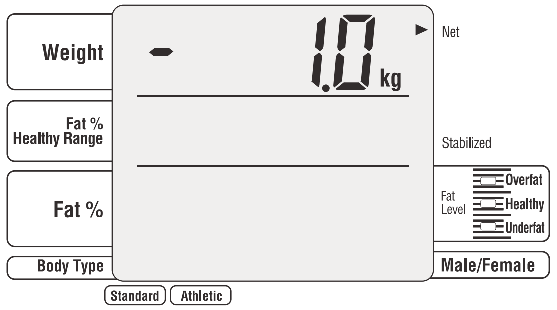

How to read the display <kg mode>

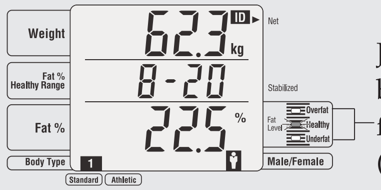

Judgment result based on the body fat percentage. (☞page 42.)

How to read the display <lb mode>

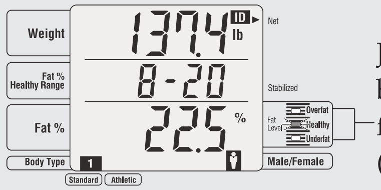

Judgment result based on the body fat percentage. (☞page 42.)

Operating Instructions (Target body fat %)

1 Input the target body fat %.

Input it by pressing (0) – (9).

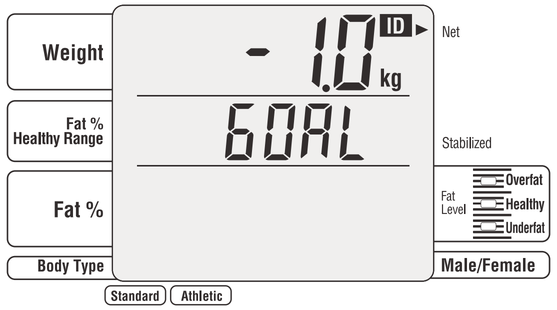

Note

- This screen is displayed if ON is set in the "setting ON or OFF of the target body fat %" (☞ page 21).
- The target body fat % can be input from 4-55.
- If it is mistakenly input,
  ⇨press (CE) (the input is deleted).

If the number of sheet to print is set to "0", target body fat % setting function will be OFF, automatically.
If the target body fat is set to 0 or nothing, the target body fat % will not print.

⚠Caution Before you start a body weight management program and set the appropriate personal body fat %, please consult your doctor. Tanita is not responsible for setting the appropriate target body fat % for specific individuals.

Operating Instructions (when using as a scale)

1 Press (ON/OFF) to turn on the power.

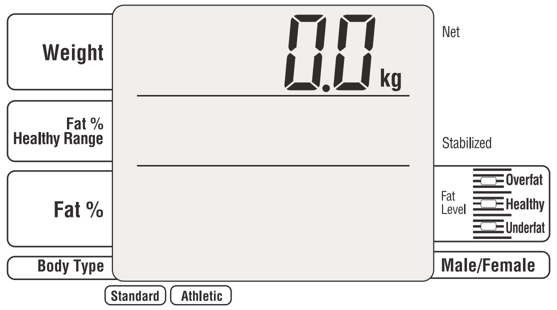

Note

- **There is no automatic weight lock function.**

2 Check that the body weight is selected and input the clothes weight.

Input it by pressing (0) – (9) and (.).

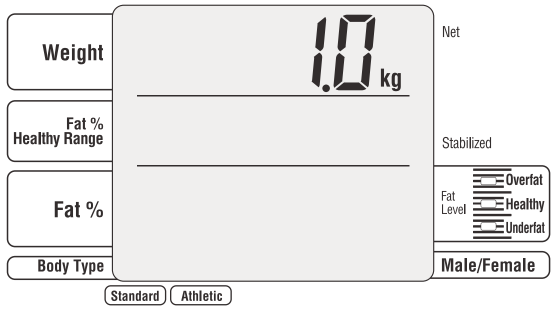

Note

- The clothes weight (preset tare) can be input in the range of 0.0-20.0lb or 0.0-10.0kg.
  *0.2lb / 0.1kg increments
  *Unit is changed by (kg / lb) key.
- If it is mistakenly input,
  ⇨press (CE) (the input is deleted).

3 Press (Enter / Next).

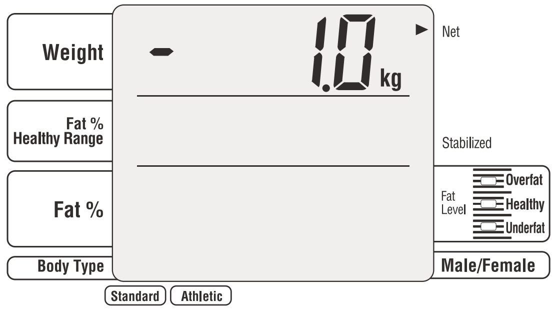

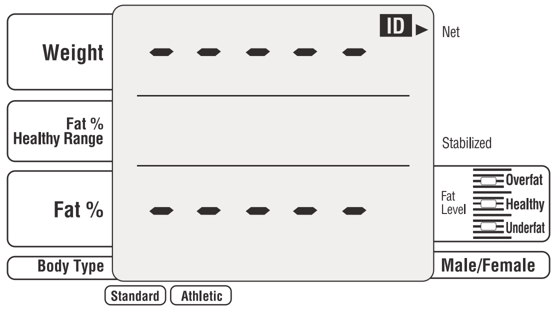

Note

- **This screen is not displayed if OFF is set in the "setting with or without an ID" (☞ page 17). (The "measurment start" screen is displayed.)**

Operating Instructions (when using as a scale (continued))

4 Input an ID number.

Input it by pressing 0 – 9.

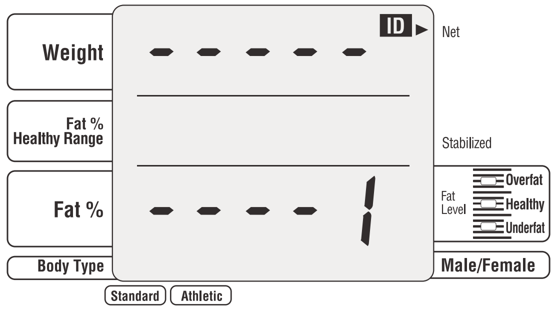

Note

- **This screen is not displayed in the case that OFF is set in the "setting with or without an ID" (☞ page 17).**
- The ID number can be input from 0 – 9999999999.
- If it is mistakenly input,
  ⇨press CE (the input is deleted).
- If CE is pressed in the state that an ID number is not input, it returns to the "measurement start."

5 Press Enter / Next .

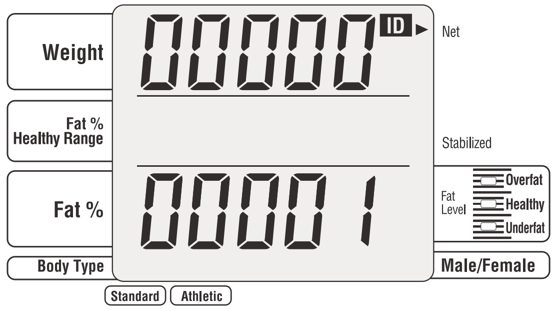

Note

- **This screen is not displayed in the case that OFF is set in the "setting with or without an ID" (☞ page 17).**

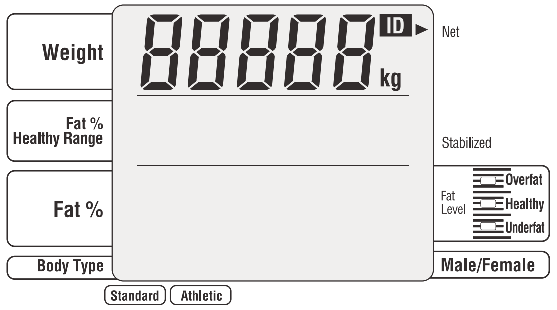

The lamp flashes for "Step on."

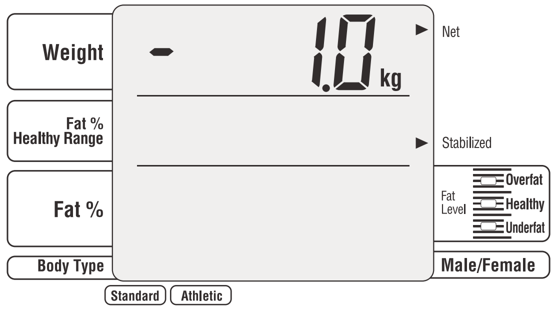

Note

- If CE is pressed, it returns to the previous screen.

6 Measurement completion

It is automatically printed out. (In the case that other than 0 is set in the "Setting the number of sheets to print" (☞page 15).

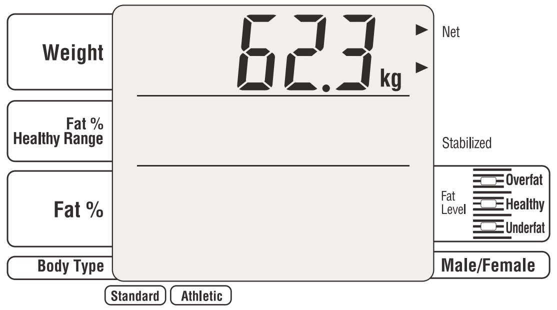

Step off the platform.
It returns to the "measurement start" screen.

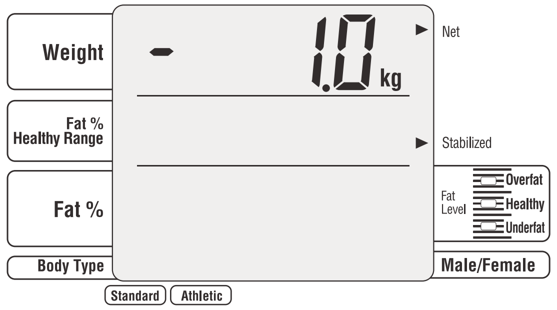

Various criteria

- What is body fat percentage? (Applicable age 5 - 99)

Body fat percentage is the amount of body fat as a proportion of your body weight.
Reducing excess levels of body fat has shown to reduce the risk of certain conditions such as high blood pressure, heart disease,diabetes and cancer. The chart below shows the healthy ranges for body fat.

Body Fat Ranges for Standard Children [1]
Body Fat Ranges for Standard Adults [2]

[1] *Susan Jebb et al. Obesity Reseach[o2] 2004;12:A156-157 "New Body Fat Reference Curves for children"*

[2] *Gallagher D et al. Am J Clin Nutr 2000,72:694-701. "Healthy percentage body fat ranges:an approach for developing guidelines based on body mass index."*

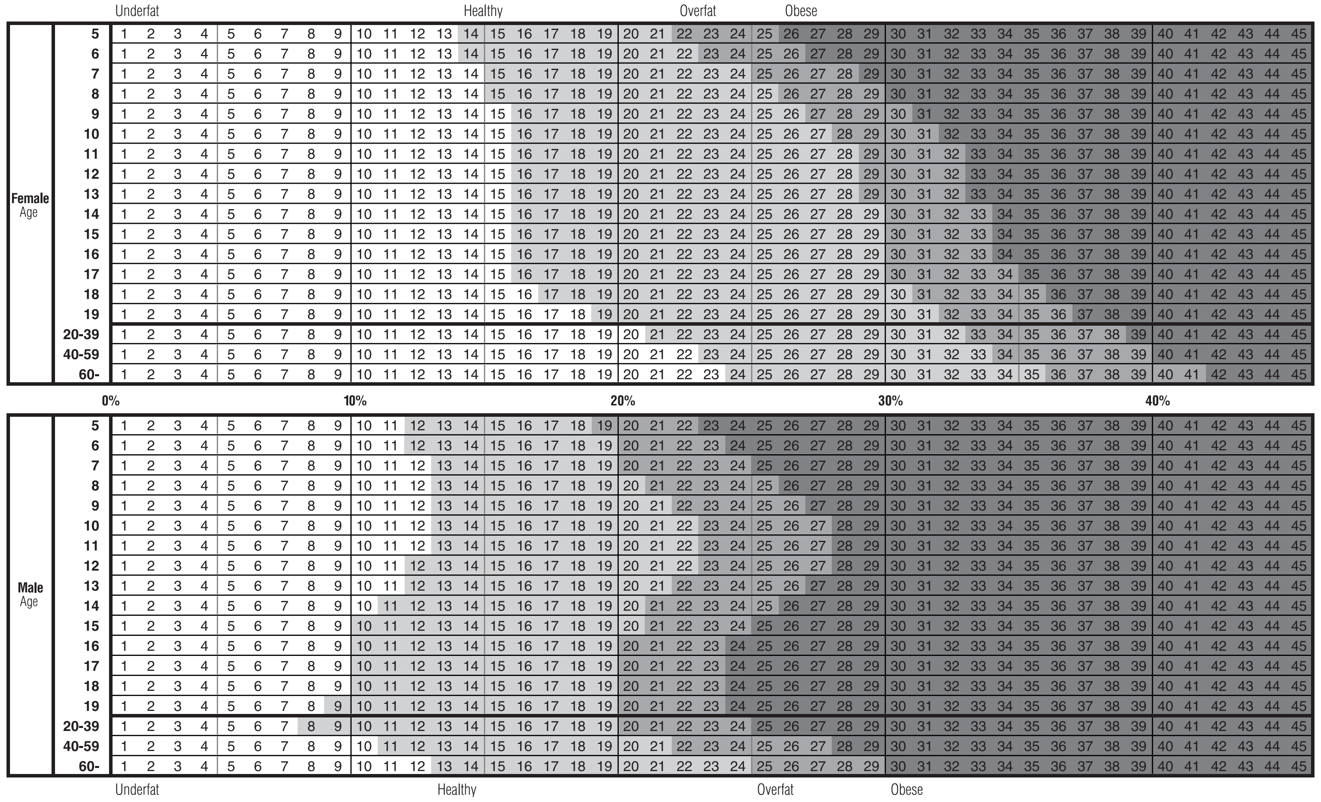

Copyright (C) 2004 TANITA Corporation. All Rights Reserved.

Healthy Range Indicator

Your Body Composition Analyzer automatically compares your body fat percentage reading to the Healthy Body Fat Range chart. After your body fat percentage has been calculated, the LED on the side of the display will light up, identifying where you fall within the Body Fat Ranges for your age and gender.

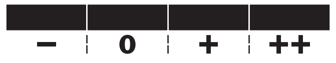

(-) : Underfat; below the healthy body fat range. Increased risk for health problems.
(0) : Healthy; within the healthy body fat percentage range for your age/gender.
(+) : Overfat; above the healthy range. Increased risk for health problems.
(++) : Obese; high above the healthy body fat range. Greatly increased risk of obesity-related health problems.

***Note** : Athletes may have a lower body fat range depending on their particular sport or activity.

- What is total body water percentage? (Applicable age 18 - 99)

Total Body Water Percentage is the total amount of fluid in a person's body expressed as a percentage of their total weight.
Water plays a vital role in many of the body's processes and is found in every cell, tissue and organ. Maintaining a healthy total body water percentage will ensure the body functions efficiently and will reduce the risk of developing associated health problems.

Your body water levels naturally fluctuate throughout the day and night. Your body tends to be dehydrated after a longnight and there are differences in fluid distribution between day and night. Eating large meals, drinking alcohol, menstruation, illness, exercising, and bathing may cause variations in your hydration levels.

Your body water percentage reading should act as a guide and should not be used to specifically determine your absolute recommended total body water percentage. It is important to look for long-term changes in total body water percentage and maintain a consistent, healthy total body water percentage.

Drinking a large quantity of water in one sitting will not instantly change your water level. In fact, it will increase your body fat reading due to the additional weight gain. Please monitor all readings over time to track the relative change.

Every individual varies but as a guide the average total body water percentage ranges for a healthy adult are:
Female : 45 to 60%
Male : 50 to 65%
Source : Based on Tanita's Internal Research

Note: The total body water percentage will tend to decrease as the percentage of body fat increases. A person with a high percentage of body fat may fall below the average body water percentage. As you lose body fat the total body water percentage should gradually move towards the typical range given above.

- What is visceral fat rating? (Applicable age 18 - 99)

This feature indicates the rating of visceral fat in your body.
Visceral fat is the fat that is in the internal abdominal cavity, surrounding the vital organs in the trunk (abdominal) area. Research shows that even if your weight and body fat remains constant, as you get older the distribution of fat changes and is more likely to shift to the trunk area especially post menopause. Ensuring you, have healthy levels of visceral fat my reduce the risk of certain diseases such as heart disease, high blood pressure, and the onset of type 2 diabetes.

The Tanita Body Composition Analyzer will provide you with a visceral fat rating from 1 – 59.

Rating from 1 to 12
Indicates you have a healthy level of visceral fat. Continue monitoring your rating to ensure that it stays within the healthy range.

Rating from 13 – 59
Indicates you have an excess level of visceral fat. Consider making changes in your lifestyle possibly through diet changes or increasing exercise.

Source : 1) Tanita Institute Contract Study. Algorithm Development for Estimating Visceral Fat Rating. SB Heymsfield MD. Columbia University College of Physicians and Surgeons 2004.
2) Wang, Z., et al. Japanese-American Differences in Visceral Adiposity and a Simplified Estimation Method for Visceral Adipose Tissue. North American Association for the Study of Obesity. Annual Meeting. Abstract 518-P. 2004

Note:
- Even if you have a low body fat rate, you may have a high visceral fat level.
- For medical diagnosis, consult a physician.

- What is basal metabolic rate (BMR)? (Applicable age 18 - 99)

WHAT IS BMR?
Your Basal Metabolic Rate(BMR) is the minimum level of energy your body needs when at rest to function effectively including your respiratory and circulatory organs, neural system, liver, kidneys, and other organs. You burn calories when sleeping.

About 70% of calories consumed every day are used for your basal metabolism. In addition, energy is used when doing any kind of activity however; the more vigorous the activity is the more calories are burned. This is because skeletal muscle (which accounts for approximately 40% of your body weight) acts as your metabolic engine and uses a large amount of energy. Your basal metabolism is greatly affected by the quantity of muscles you have, therefor increasing your muscle mass will help increase your basal metabolism.

By studying healthy individuals, scientists have found that as people age, their metabolic rate changes. Basal metabolism rises as a child matures. After a peak at the age of 16 or 17, it typically starts to decrease gradually.

Having a higher basal metabolism will increase the number of calories used and help to decrease the amount of body fat. A low basal metabolic rate will make it harder to lose body fat and overall weight.

HOW DOES A TANITA BODY COMPOSITION ANALYZER CALCULATE BMR?
The basic way of calculating Basal Metabolic Rate BMR is a standard equation using weight and age. Tanita has conducted in-depth research into the relationship of BMR and body composition giving a much more accurate and personalized reading for the user based on the impedance measurement. This method has been medically validated using indirect calorimetry (measuring the breath composition).*

* Reliability on equation for Basal Metabolic Rate: At 2002 Nutrition Week : A Scientific and Clinical Forum and Exposition Title: International Comparison: Resting Energy Expenditure Prediction Models: The American Journal of Clinical Nutrition

- What is metabolic age? (Applicable age 18 - 99)

This feature calculates your BMR and indicates the average age associated with that type of metabolism.
If your BMR Age is higher than your actual age, it is an indication that you need to improve your metabolic rate. Increased exercise will build healthy muscle tissue, which will improve your metabolic age.

You will obtain a reading between 12 and 90. Under 12 will be displayed as “12” and over 90 displayed as “90”.

Eng
How to use (Various criteria)

- What is mascle mass? (Applicable age 18 - 99)

This feature indicates the weight of muscle in your body. the muscle mass displayed includes the skeletal muscles, smooth muscles (such as cardiac and digestive muscles) and the water contained in these muscles.
Muscles play an important role as they act as an engine in consuming energy. As your muscle mass increase, your energy consumption increases helping you reduce excess body fat levels and lose weight in a healthy way.

What is physique rating?
This feature assesses your physique according to the ratio of body fat ad muscle mass in your body.
As you become more active and reduce the amount of body fat, your physique rating will also change accordingly. Even though your weight may not change, your muscle mass and body fat levels may be changing making you healthier and at lower risk of certain diseases.
Each person should set their own goal of which physique they would like and follow a diet and fitness program to meet that goal.

Result	Physique Rating	Explanation
1	Hidden obese	**Small Frame Obese** This person seems to have a healthy body type based on physical appearance; however, they have a high body fat % with low muscle mass level.
2	Obese	**Medium Frame Obese** This person has a high body fat percentage, with a moderate muscle mass level.
3	Solidly-built	**Large Frame Obese** This person has both a high body fat % and a high muscle mass.
4	Under exercised	**Low Muscle & Average Body Fat%** This person has an average body fat % and a less than average muscle mass level
5	Standard	**Ave. Muscle & Ave. Body Fat %** This person has average levels of both body fat and muscle mass.
6	Standard Muscular	**High Muscle & Ave. Body Fat % (Athlete)** This person has an average body fat % and higher than normal muscle mass level.
7	Thin	**Low Muscle & Low Fat** This person has both a lower than normal body fat % and muscle mass level.
8	Thin and muscular	**Thin and muscular (Athlete)** This person has lower than normal body fat % while having adequate muscle mass.
9	Very Muscular	**Very Muscular (Athlete)** This person has lower than normal body fat % while having above average muscle mass.

Source : Data from Columbia University (New York) & Tanita Institute (Tokyo)

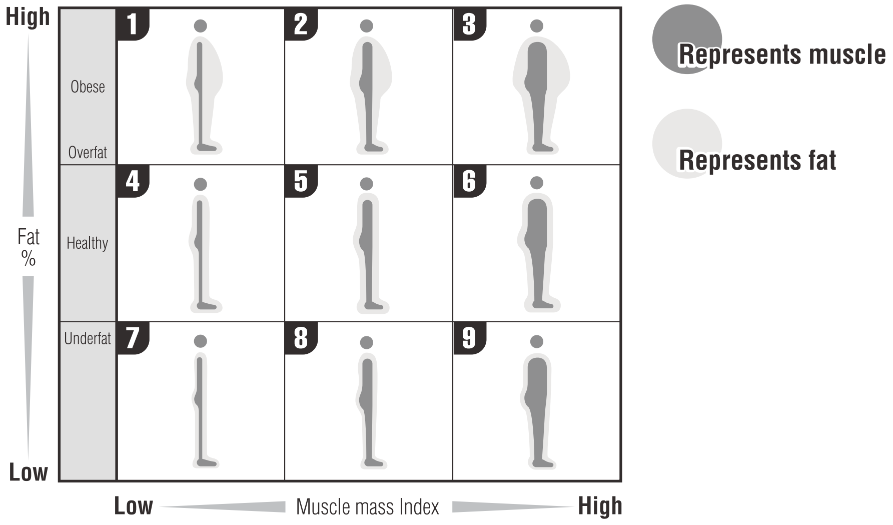

Index of amount of muscle against height = Muscle mass (kg) / height (cm)^2

- What is bone mass? (Applicable age 18 - 99)

This feature indicates the amount of bone (bone mineral level, calcium or other minerals) in the body. Research has shown that exercise and the development of muscle tissue are related to stronger, healthier bones. While bone structure is unlikely to make noticeable changes in a short period, it is important that you develop and maintain healthy bones by having a balanced diet and plenty of exercise. People worried about bone disease should consult their physician. People who suffer from osteoporosis or low bone densities due to advanced age, young age, pregnancy,hormonal treatment or other causes, may not get accurate estimations of their bone mass.

Below is the result of estimated bone masses of persons aged 20 to 40, who are said to have the largest amounts of bone masses, by weight. (Source : Tanita Body Weight Science Institute)

Please use the below charts as a guide to compare your bone mass reading.

Women : Average of estimated bone mass

Weight (lb)		
Less than 110 lb	110lb - 165 lb	165 lb and up
4.3 lb	5.3 lb	6.5 lb

Weight (kg)		
Less than 50 kg	50 kg - 75 kg	75 kg and up
1,95 kg	2,40 kg	2,95 kg

Men : Average of estimated bone mass

Weight (lb)		
Less than 143 lb	143 lb - 209 lb	209 lb and up
5.9 lb	7.3 lb	8.1 lb

Weight (kg)		
Less than 65 kg	65 kg - 95 kg	95 kg and up
2,66 kg	3,29 kg	3,69 kg

Note: Persons described below may obtain varying readings and should take the values given for reference purposes only.
- Elderly persons - Women during or after menopause
- People receiving hormone therapy

"Estimated bone mass" is a value estimated statistically based on its correlation with the fat-free amount (tissues other than the fat). "Estimated bone mass" does not give a direct judgment on the hardness or strength of the bones or the risks of bone fractures. If you have concerns over your bones, you are recommended to consult a specialist physician.

Troubleshooting

- Please check the following before asking for repair.

Symptom		Please check
How to measure	Impedance measurement error<br>Err40<br>Err67	• Measure with bare feet.<br>• When the soles of your feet are dry, drop water for about 0.5 ml with the attached dropper on the electrodes before measurement.<br>• Check the input information.
How to measure	Zero point error<br>uuuuu	• Turn off the power, and remove the items on the platform and turn on the power again, and then redo the measurement.
How to measure	The measured weight is not stable.	• Is it installed at a place with vibrations?<br>• Is the platform inclined?<br>⇨Keep the platform horizontal. (☞ pages 8 and 9)<br>• Is anything caught in the gaps of the platform?<br>⇨Remove anything caught in the gaps.
Display part	Nothing is displayed even after turning on the power.	• Check that the power supply is connected correctly.
Display part	––––– is displayed.	• The weight to measure exceeds the measurement range.

Symptom		Please check
Printer	Run out of printer paper<br>P-End	• Printer paper is not supplied.<br>⇨Supply paper.<br>⇨In the case that the printer is not used, press CE and redo the initial setting.
Printer	Printer cover open<br>COPEn	• The printer cover is open.<br>⇨Properly close it.<br>⇨Check that the printer paper is not slanted.
Printer	Paper does not come out.	**Check the settings.**<br>• Is 0 set for the number of sheets to print in the "determination of the number of sheets to print?"<br>⇨Press 1 – 3. (☞ page 15)<br>• The printer may be broken.<br>⇨Contact the agent from which you have purchased the product.
Printer	The paper comes out, however, nothing has been printed.	• Is the reverse side of the printer paper set?<br>⇨Set the paper properly. (☞ page 11)<br>• The printer may be broken.<br>⇨Contact the agent from which you have purchased the product.

Note

• In the case that an error is displayed other than the above, turn off the power once, and then measure again.
If the same error is displayed repeatedly, contact our customer service center.

Connection with a personal computer

⚠ The USB interface enables input and output from this equipment.

- Specifications

Communication standards	USB
Communication method	Asynchronous Transmission
Signal speed	9600 baud
Data bit length	8 bits
Parity	None
Stop bit	1 bit
Flow control	None
Terminator	CR+LF

Note

- USB connector (B - Type 4 - pin female) are located on the back of the control box.
- Please provide your own cable as necessary as none are included.
  USB cable : A - Type 4 - pin (male) - B - Type 4 - pin (male)
- You must install the secessary driver onto your PC, available download from http://www.tanita.com.

- Transmit data

⚠ **Caution** Transmission Data is output immediately after measurement regardless of the status of the receiving equipment (personal computer, etc.). Therefore the receiving equipment needs to be ready to accept the data before measuring.

(1) Output example (in the case to kg unit)

Note

- The data are divided with commas (,) for each data.
- The terminator (end of the data) is CR (ASCII code 0DH), LF (ASCII code 0AH).
- The control data for the items 1 – 4 are for expansion. They are not used presently so the receiving side can ignore them.
- Because of its specifications, the scale will output an irrelevant signal approximately 0.1 second after data output. Make sure you ignore this signal and do not import it.

Entire body data

Model number		Serial number		ID number		Date (mm/dd/yyyy)		Time	
MO	"XXXXXX"	SN	"XXXXXXXX"	ID	"XXXXXXXXXX"	Dt	"mm/dd/yyyy"	TI	"hh:mm"

Body type		Gender		Age		Height		Preset tare	
Bt	0or2	GE	1or2	AG	XX	Hm	XXX.X	Pt	XX.X

Body weight		Fat %		Fat mass		Fat free mass		Muscle mass	
Wk	XXX.X	FW	XX.X	fW	XXX.X	MW	XXX.X	mW	XXX.X

Muscle score		Bone mass		Body water		Body water %		BMI	
sW	XX	bW	XXX.X	wW	XXX.X	ww	XX.X	MI	XXX.X

Ideal body weight		Degree of obesity		Visceral fat rating		BMR (kJ)		BMR (kcal)	
Sw	XXX.X	OV	XXX.X	IF	XX	rb	XXXXX	rB	XXXXX

BMR score		Metabolic age		Target body fat %		Predicted weight		Predicted fat mass	
rJ	XX	rA	XX	gF	XX	gW	XXX.X	gf	XXX.X

Fat to gain / lose		Impedance (50kHz)		Checksum	
gt	XXXXX.X	ZF	XXXX.X	CS	XX

(2) Output data items

<kg / cm mode>

Item	Header	format	kg / cm mode	Order of output			
				Body composition			Scale
				Adult	Athlete	Child	
Control data	{0		2 bytes fixed length	1	1	1	1
Control data	~0		1 byte fixed length	2	2	2	2
Control data	~1		1 byte fixed length	3	3	3	
Control data	~2		1 byte fixed length	4	4	4	
Model number	MO	"XXXXXX"	8 bytes fixed length ("SC-331")	5	5	5	3
Serial number	SN	"XXXXXXXX"	10 bytes fixed length	6	6	6	4
ID number	ID	"XXXXXXXXXX"	12 bytes fixed length	7	7	7	5
Date	Dt	"mm / dd / yyyy"	12 bytes fixed length ("mm / dd / yyyy")	8	8	8	6
Time	TI	"hh : mm"	7 bytes fixed length	9	9	9	7
Body type	Bt	0 or 2	1 byte fixed length (0:Standard 2:Athletic)	10	10	10	
Gender	GE	1 or 2	1 byte fixed length (1:Male 2:Female)	11	11	11	
Age	AG	XX	1-2 bytes variable length	12	12	12	
Height	Hm	XXX.X	4-5 bytes variable length (cm)	13	13	13	
Preset tare	Pt	XX.X	3-4 bytes variable length (increments 0.1kg)	14	14	14	8
Body weight	Wk	XXX.X	3-5 bytes variable length (increments 0.1kg)	15	15	15	9
Fat %	FW	XX.X	3-4 bytes variable length (increments 0.1%)	16	16	16	
Fat mass	fW	XXX.X	3-5 bytes variable length (increments 0.1kg)	17	17	17	
FFM	MW	XXX.X	3-5 bytes variable length (increments 0.1kg)	18	18	18	
Muscle mass	mW	XXX.X	3-5 bytes variable length (increments 0.1kg)	19	19		
Muscle score	sW	XX	1-2 bytes variable length (1-24)	20	20		
Bone mass	bW	XXX.X	3-5 bytes variable length (increments 0.1kg)	21	21		
Body water	wW	XXX.X	3-5 bytes variable length (increments 0.1kg)	22	22		
Body water %	ww	XX.X	4 bytes fixed length (increments 0.1%)	23	23		
BMI	MI	XXX.X	3-5 bytes variable length (increments 0.1)	24	24	19	
Ideal body weight	Sw	XXX.X	3-5 bytes variable length (increments 0.1kg)	25			
Degree of obesity	OV	XXX.X	3-5 bytes variable length (increments 0.1%)	26			
Visceral fat rating	IF	XX	1-2 bytes variable length	27	25		
BMR (kJ)	rb	XXXXX	1-5 bytes variable length (increments 1kJ)	28	26		
BMR (kcal)	rB	XXXXX	1-5 bytes variable length (increments 1kcal)	29	27		
BMR score	rJ	XX	1-2 bytes variable length (1-24)	30	28		
Metabolic age	rA	XX	2 bytes fixed length	31	29		
Target body fat %	gF	XX	1-2 bytes variable length (4-55)	32	30	20	
Predicted weight	gW	XXX.X	3-5 bytes variable length (increments 0.1kg)	33	31	21	
Predicted fat mass	gf	XXX.X	3-5 bytes variable length (increments 0.1kg)	34	32	22	
Fat gain / lose	gt	XXXXX.X	3-7 bytes variable length (increments 0.1kg)	35	33	23	
Impedance (50kHz)	ZF	XXXX.X	5-6 bytes variable length (increments 0.1)	36	34	24	
Checksum	CS	XX	2 bytes fixed length	37	35	25	10

Output sample
• Oct-09-2009, 19:59, Standard, Male, 25 years old, Height 174.5cm
{0,16,~0,1,~1,1,~2,1,MO,"SC-331",SN,"0000000002",ID,"0000000112",Dt,"10/09/2009",TI,"19:59",Bt,0,GE,1,AG,25, Hm,174.5,Pt,1.5,Wk,65.6,FW,20.3,fW,13.3,MW,52.3,mW,49.6,sW,0,bW,2.7,wW,33.6,ww,51.2,MI,22.7, Sw,63.6,OV,-5.8,IF,10,rb,7134,rB,1705,rJ,10,rA,30,gF,4,gW,123.4,gf,123.4,gt,-123.4,ZF,490.9,CS,C7

<lb / ft-in mode>

Item	Header	format	lb / ft-in mode	Order of output			
				Body composition			Scale
				Adult	Athlete	Child	
Control data	{0		2 bytes fixed length	1	1	1	1
Control data	~0		1 byte fixed length	2	2	2	2
Control data	~1		1 byte fixed length	3	3	3	
Control data	~2		1 byte fixed length	4	4	4	
Model number	MO	"XXXXXX"	8 bytes fixed length ("SC-331")	5	5	5	3
Serial number	SN	"XXXXXXXX"	10 bytes fixed length	6	6	6	4
ID number	ID	"XXXXXXXXXX"	12 bytes fixed length	7	7	7	5
Date	Dt	"mm / dd / yyyy"	12 bytes fixed length ("mm / dd / yyyy")	8	8	8	6
Time	TI	"hh : mm"	7 bytes fixed length	9	9	9	7
Body type	Bt	0 or 2	1 byte fixed length (0:Standard 2:Athletic)	10	10	10	
Gender	GE	1 or 2	1 byte fixed length (1:Male 2:Female)	11	11	11	
Age	AG	XX	1-2 bytes variable length	12	12	12	
Height	Hi	XX.X	4 bytes variable length (in)	13	13	13	
Preset tare	Pt	XX.X	3-4 bytes variable length (increments 0.2lb)	14	14	14	8
Body weight	Wp	XXX.X	3-5 bytes variable length (increments 0.2lb)	15	15	15	9
Fat %	FW	XX.X	3-4 bytes variable length (increments 0.1%)	16	16	16	
Fat mass	fW	XXX.X	3-5 bytes variable length (increments 0.2lb)	17	17	17	
FFM	MW	XXX.X	3-5 bytes variable length (increments 0.2lb)	18	18	18	
Muscle mass	mW	XXX.X	3-5 bytes variable length (increments 0.2lb)	19	19		
Muscle score	sW	XX	1-2 bytes variable length (1-24)	20	20		
Bone mass	bW	XXX.X	3-5 bytes variable length (increments 0.2lb)	21	21		
Body water	wW	XXX.X	3-5 bytes variable length (increments 0.2lb)	22	22		
Body water %	ww	XX.X	4 bytes fixed length (increments 0.1%)	23	23		
BMI	MI	XXX.X	3-5 bytes variable length (increments 0.1)	24	24	19	
Ideal body weight	Sw	XXX.X	3-5 bytes variable length (increments 0.2lb)	25			
Degree of obesity	OV	XXX.X	3-5 bytes variable length (increments 0.1%)	26			
Visceral fat rating	IF	XX	1-2 bytes variable length	27	25		
BMR (kJ)	rb	XXXXX	1-5 bytes variable length (increments 1kJ)	28	26		
BMR (kcal)	rB	XXXXX	1-5 bytes variable length (increments 1kcal)	29	27		
BMR score	rJ	XX	1-2 bytes variable length (1-24)	30	28		
Metabolic age	rA	XX	2 bytes fixed length	31	29		
Target body fat %	gF	XX	1-2 bytes variable length (4-55)	32	30	20	
Predicted weight	gW	XXX.X	3-5 bytes variable length (increments 0.2lb)	33	31	21	
Predicted fat mass	gf	XXX.X	3-5 bytes variable length (increments 0.2lb)	34	32	22	
Fat gain / lose	gt	XXXXX.X	3-7 bytes variable length (increments 0.2lb)	35	33	23	
Impedance (50kHz)	ZF	XXXX.X	5-6 bytes variable length (increments 0.1)	36	34	24	
Checksum	CS	XX	2 bytes fixed length	37	35	25	10

Eng

When necessary
(Connection with a personal computer)

Body composition measurement by the BIA method.

Introduction

This equipment provides estimated values for each measured value of body fat percentage, fat mass, fatfree mass, muscle mass and bone mass by the DXA method, estimated value for the total body water measured value by the dilution method and estimated value for the visceral fat rating by MRI method using the Bioelectrical Impedance Analysis (BIA method).
For measurement, a mode must be selected based on body type.
1) Standard (for 5-99 years of age)
2) Athletic (for Athletic persons who exercise considerably more than non-athlete)
Making a distinction by body type in the measurement mode produces more reliable body composition measurements for athletic persons, whose body compositions differ from those of average persons.

- Principles of body composition measurement

BIA is a means of measuring body composition – fat mass, predicted muscle mass, etc. – by measuring bioelectrical impedance in the body. Fat within the body allows almost no electricity to pass through, while electricity passes rather easily through water, much of which is found in muscles. The degree of difficulty with which electricity passes through a substance is known as the electrical resistance, and the percentage of fat and other body constituents can be inferred from measurements of this resistance.
The Tanita Body Composition Analyzer measures body composition using a constant current source with a high frequency current (50kHz, 90µA). The 8 electrodes are positioned so that electric current is supplied from the electrodes on the tips of the toes of both feet, and voltage is measured on the heel of both feet. The current flows into the upper limbs or lower limbs, depending on the body part(s) to be measured.

- What is the DXA method?

DXA was originally designed to measure bone mineral content, but in the full-body scan mode the body fat percentage, fat mass, and fat free mass of individual body parts (arms, legs, trunk) can also be measured. The image below shows one example of body composition measurement results obtained by DXA.

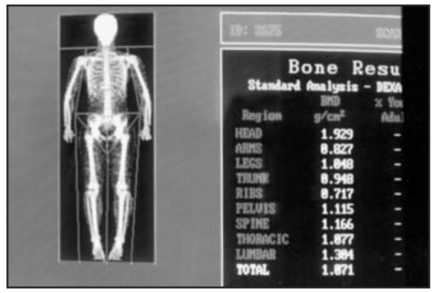

Body composition measurement results obtained by DXA (Lunar Co., Ltd; DPX-L)

- What is dilution method?

In the dilution method, a labeled substance for a known amount is given and the concentration in equilibrium diffusing evenly is measured to obtain the total amount of the solvent that dilutes the labeled substance.
To measure the total body water (TBW), deuterium oxide (D_2O) is generally used as the labeled substance. Deuterium oxide uses the overall total body water as dilution space so the total body water can be obtained. To obtain the extracellular fluid amount, sodium bromide (NaBr) is used as a labeled substance. Bromine (Br) is said to not enter the inside of cells, and uses extracellular fluid as the dilution space.

- What is the visceral fat?

Visceral Adipose Tissue (VAT) has been associated with increased risk of developing lifestyle-related diseases. Accordingly, knowing and periodically checking the estimated VAT accumulation serves as one factor of a number of factors in assessing the prevention of lifestyle related diseases.

Tanita has developed the technology for measuring the VAT accumulation risk through bioelectrical impedance analysis (BIA) in comparison with image analysis applied to magnetic resonance imaging (MRI), in addition to the established technology for measuring the percent of body fat. The VAT accumulation risk is calculated by estimating the VAT area by the BIA method on the basis of MRI image processing. This method has a higher correlation than the estimation of the VAT accumulation risk based on BMI or abdominal circumference (waist circumference), allowing estimation that corresponds more precisely to individuals.
* The VAT area by MRI is calculated by carrying out an image processing of the cross section of the lumber vertebra L4-L5 regions.

(Fig. 1 - Fig. 3: Research results by N. Y. Columbia University and Jikei University Published by the North American Association for the Study of Obesity [NAASO] in 2004.)

<Fig. 1> Relationship between VAT Area and BMI

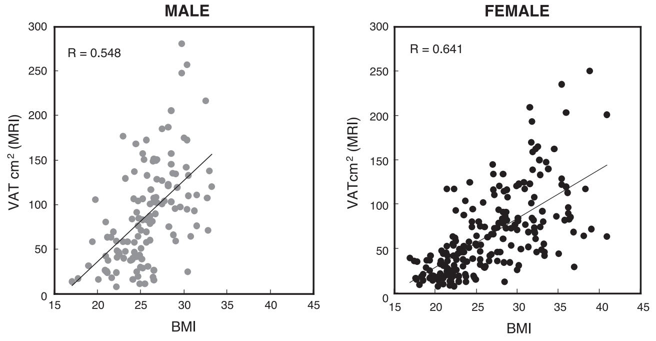

<Fig. 2> Relationship between VAT Area and Waist Circumference

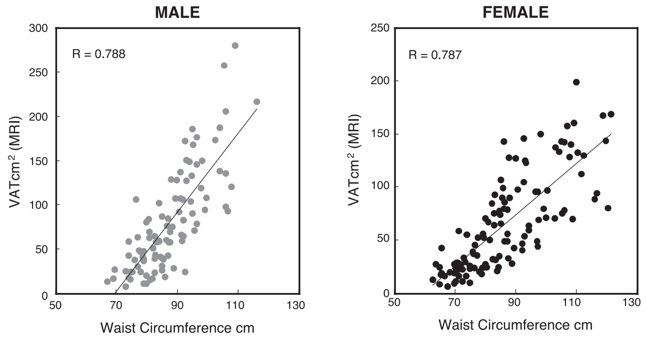

<Fig. 3> Relationship between VAT Area by MRI and Estimated VAT Area by Tanita's BIA

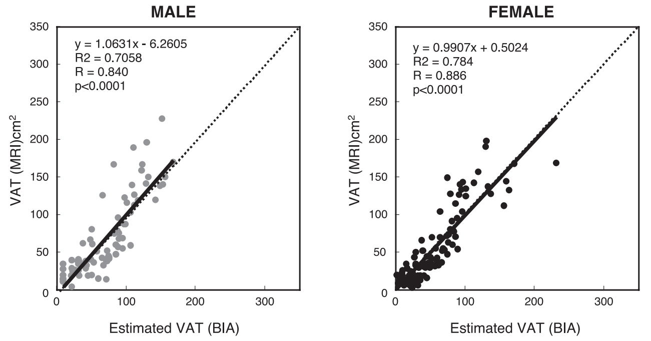

- Factors giving errors in measurement

In the BIA method, impedance is measured and the body composition is calculated based on the value. It is known that impedance changes by the amount of the total body water that occupies about 60% of weight and the change in its distribution and temperature change. Therefore, for the purpose of research or for daily repeating of measurements, the measurement conditions must be kept constant. Measurement under the changing conditions of temperature and total body water distribution or blood flow volume of extremities due to exercising, taking a bath, etc., affects the measurement result since the electric resistance in the body also changes.

Therefore, it is recommended to measure under the following conditions for stable measurement.

1) 3 hours have passed after getting up and normal lifestyle activities are carried out during this period. (The impedance transits staying at a high level if you remain sitting after getting up or drive a car, etc.)
2) 3 hours or more have passed after eating. (For 2 – 3 hours after eating, the impedance has a tendency to decrease.)
3) 12 hours or more have passed after vigorous exercise for measurement. (The tendency toward changes in impedance is not stable depending on the type and rigorousness of the exercise.)
4) If possible urinate before taking measurement.
5) For repeated measurements, measure at the same hour as much as possible. (At the same time of measurement of weight, the measurements can be made more stable by measuring at the same time of the day)

Very stable measured values can be obtained by measuring under the above conditions.

And in the development of this equipment, the following 6 items were set as conditions for the regression equation.

1) Prohibition of alcohol intake for 12 hours before measurement
2) Prohibition of excessive exercise for 12 hours before measurement.
3) Prohibition of excessive eating and drinking the day before measurement
4) Prohibition of eating and drinking for 3 hours before measurement
5) Urination just before measurement
6) Avoid measurements during menstruation (for female)

2) Inter-day changes

The diagrams below offer examples of actual measurements made of inter-day changes. A study was done to determine the degree of change in the impedance between the feet during dehydration; the first two days represent a normal daily routine, while in the latter two days a state of dehydration was induced using a sauna.

No significant inter-day change was measured in body weight, impedance between the feet, or body fat percentage during the normal daily routine. During the dehydrated state, however, a drop in body weight of 1kg was noted, with the impedance between the feet rising approximately 15Ω on the first day of dehydration and 30-35Ω on the second day. As a result, body fat percentage was up by around 1% on the first day of dehydration and by 1.5-2% on the second day.

As mentioned earlier, impedance increases when body weight is reduced (such as by dehydration), and decreases when body weight is increased through excess consumption of food and drink. The inter-day change in impedance is thus inversely proportional to the change in body weight.

Intra-day fluctuations (Weight)

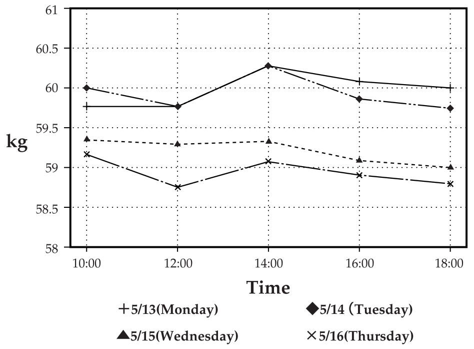

Intra-day fluctuations (Impedance value)

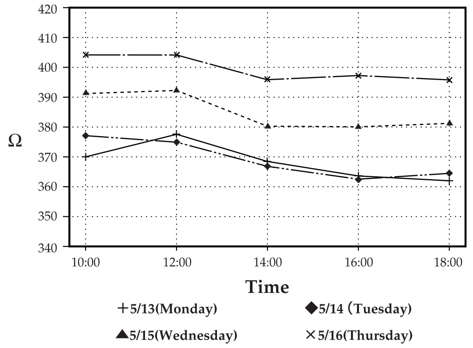

These inter-day changes stem from such causes as:

1) Temporary increases in body weight (total body water) through overeating and overdrinking
2) Dehydration due to heavy sweating during vigorous exercise
3) Dehydration due to alcohol consumption or the use of diuretics
4) Dehydration due to heavy sweating during saunas, etc.

Accordingly, it is recommended that instructions be provided to the subject to help eliminate these causes when accurate measurements are needed.

The Regression Formula for Basal Metabolic Rate (BMR)

It has long been said among medical and nutritional specialists that "The Basal Metabolic Rate (BMR) is more determined by the Fat Free Mass (FFM) than by the body weight" (Persons of a given body weight with a higher FFM will have a higher BMR), and that from the aspect of evaluating the body composition, should be estimated from the FFM. In addition, in cases of simple estimation formulae which can calculate from the height, weight and age, without evaluating the body composition, there was a problem with excessively high evaluations being given to obese persons with large body weight, and conversely excessively small BMR evaluations given to muscular athletes, though these are not as many in number.

Currently, the BMR estimation recursion formula developed by Tanita, the manufacturer of body composition analyzers, based on their research, works by multiple regressive analysis using this FFM, and has a higher degree of accuracy in the individual differences in body composition. In order to derive the BMR, resting respiratory metabolism (Resting Energy Expenditure: REE) was measured using a breath gas analysis device, and this estimation recursion formula was created based on this data.

<Figure 1> The Relationship Between Resting Energy Expenditure (REE) According to Breath Gas Analysis and Weight, FFM

(Presented at Nutrition Week, Held in San Diego in 2002)

As shown in Figure 1: the REE (BMR) has a stronger relationship to the FFM than to body weight, and a difference is visible between males and females in the distribution trends. We see that in principle that we should calculate from the FFM rather than by the old formula centred on the relationship with weight.

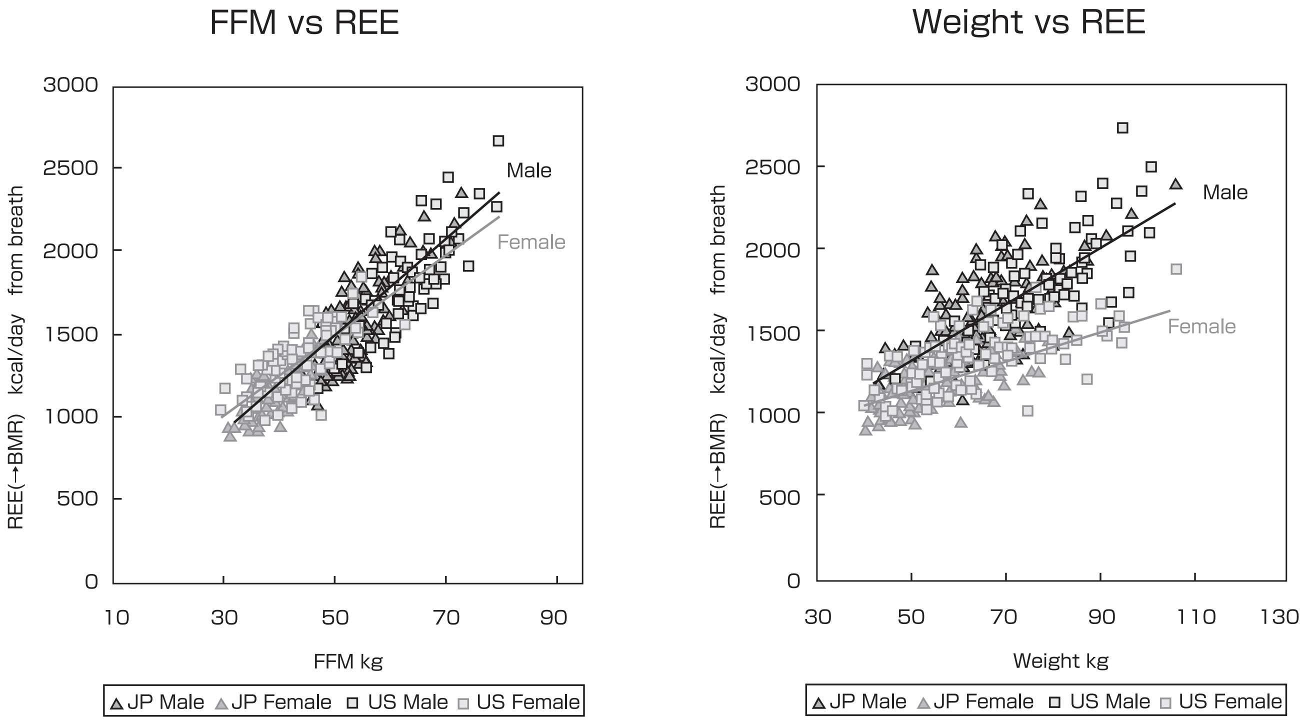

<Figure 2> Comparison of BMR Values from the TANITA Multiple regression model and Breath Analysis

(Presented at Nutrition Week Held in San Diego in 2002)

The current BMR retrogression formula is a formula which acts on the principle of using the FFM value from the results of body composition measurement according to the BIA, A good relationship is shown in the BMR value based on actual breath analysis REE or R=0.9 (p<0.0001). These results were presented at the First Annual Nutrition Week (American College of Nutrition, American Society for Clinical Nutrition, American Society for Parenteral and Enteral Nutrition, North American Association for the Study of Obesity) held in 2002 in San Diego.

NOTE: This model has been calibrated for those between ages of 18-84. Those individuals outside of this age range may not be obtain accurate readings.

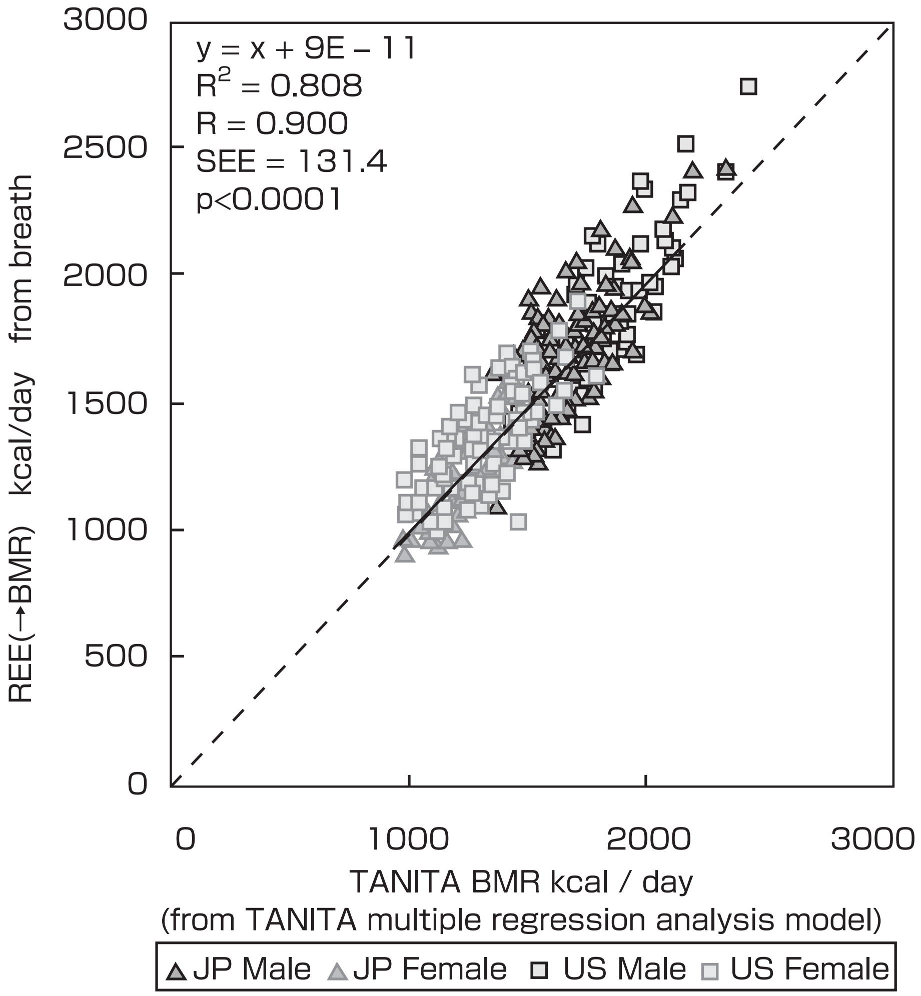

Specifications

Model number		SC-331S	
Power source		AC adapter (included) Center Minus	
		Input Voltage: 100-240 VAC 50/60 Hz 1.5A Output Voltage: 7 VDC	
		Rated Current: 4 A No Load Input Voltage: 7VDC	
Power Consumption		28 W	
Impedance Measurement	Measurement System	Tetra polar Bioelectrical Impedance Analysis	
	Measurement Frequency	50 kHz	
	Measurement Current	90 µA	
	Electrode Material	Pressure Contact Stainless Steel Foot Pads	
	Measurement Style	Between Both Feet	
	Measurement Range	150 - 1200Ω	
	Accuracy at first calibration	± 2%	
Weight Measurement	Measurement System	Strain Gauge Load Cell	
	Maximum Capacity / Minimum Graduation	270 kg / 0.1 kg<br>600 lb / 0.2 lb	
	Accuracy at first calibration	± 0.2 kg / ± 0.4 lb	
Input Items	Clothes Weight	0 - 10 kg / 0.1 kg increments	0 - 20 lb / 0.2 lb increments
	Gender	Male / Female	
	Body Type	Standard (5 - 99 years) / Athletic (18 - 99 years)	
	Age	5 - 99 years old / 1 year increments	
	Height	90.0 - 249.9 cm / 0.1 cm increments	3 ft - 7 ft 11.5 in / 0.5 in increments
	Target Body Fat %	4 - 55 %	

Output Items	Display	Target Body Fat %	4 - 55 %	
		Weight	0 - 270 kg / 0.1 kg increments	600lb / 0.2 lb increments
		Gender	Male / Female	
		Body Type	Standard / Athletic	
		Age	5 - 99 years old / 1 year increments	
		Height	90.0 - 249.9 cm / 0.1 cm increments	3 ft - 7 ft 11.5 in / 0.5 in increments
		FAT%	3 - 75% / 0.1% increments	
	Print-out	Logo	TANITA LOGO (240x64 dot)	
		Model number	SC-331S	
		Date and Time	2009 / 1 / 1 - 2099 / 12 / 31	
		Serial Number	00000001 - 99999999	
		ID	0000000000 - 9999999999	
		Body Type	Standard (5 - 99 years) / Athletic (18 - 99 years)	
		Gender	Male / Female	
		Age	5 - 99 years old / 1 year increments	
		Height	90.0 - 249.9 cm / 0.1 cm increments	3 ft - 7 ft 11.5 in / 0.5 in increments
		Clothes Weight	0 - 10 kg / 0.1 kg increments	0 - 20 lb / 0.2 lb increments
		Weight	0 - 270 kg / 0.1 kg increments	600lb / 0.2 lb increments
		FAT%	3 - 75% / 0.1% increments	
		Fat Mass	0.1 kg increments	0.2 lb increments
		FFM	0.1 kg increments	0.2 lb increments
		Muscle Mass	0.1 kg increments	0.2 lb increments
		TBW	0.1 kg increments	0.2 lb increments
		TBW%	15 - 85% / 0.1% increments	
		Predicted Fat Mass	0.1 kg increments	0.2 lb increments
		BMR	1 kJ increments / 1 kcal increments	
		Metabolic Age	1 years increments (12 - 90 years)	
		Visceral FAT Rating	1 Level increments (1-59 Level)	
		BMI	0.1 increments	
		Predicted Weight	0.1 kg increments	0.2 lb increments
		Desirable range	3 - 75% / 0.1% increments	
		Indicator - FAT %		
		Indicator - BMI		
		Indicator - Visceral FAT Level		
		Indicator - Muscle Mass		
		Indicator - BMR		
		Physique Rating		
		Impedance	150 - 1200Ω	
Display			3 Rows, 5 Digits LCD	
Output Data Interface			USB	
Temperature Range of Usage			32°F to 95°F / 0°C to 35°C	
Relative Humidity			30 - 80% (without condensation)	
Weight of Equipment (Remote Display Version)			6.8 kg	15.0 lb
Weight of Equipment (Column Mounted Version)			12.1kg	26.7 lb
Size	Platform		372 × 375 × 101 mm (14.6 × 14.8 × 4.0 in)	
	Height (Column Mounted Version)		1024 mm (40.3in)	

Eng

USA and Canada

Federal Communications Commission and Canadian ICES Notice

This equipment has been tested and found to comply with the limits for a Class B digital device, pursuant to Part 15 of the FCC Rules and Canadian ICES-003. These limits are designed to provide reasonable protection against harmful interference in a residential installation. This equipment generates, uses and can radiate radio frequency energy and, if not installed and used in accordance with the instructions, may cause harmful interference to radio communications. However, there is no guarantee that interference will not occur in a particular installation. If this equipment does cause harmful interference to radio or television reception, which can be determined by turning the equipment off and on, the user is encouraged to try to correct the interference by one or more of the following measures:

- Reorient or relocate the receiving antenna.
- Increase the separation between the equipment and receiver.
- Connect the equipment into an outlet on a circuit different from that to which the receiver is connected.
- Consult the dealer or an experienced radio or television technician for help.

Modifications

The FCC requires the user to be notified that any changes or modifications made to this device that are not expressly approved by Tanita Corporation may void the user’s authority to operate the equipment.

Disposal

This equipment is electronic device. Please dispose of this equipment appropriately as not the genera l household waste but electronic equipment. Please follow a regional regulation when you dispose of this.

<U.S.A. representative>

TANITA® Corporation of America, Inc.

2625 South Clearbrook Drive, Arlington Heights, Illinois 60005, U.S.A. TEL: 1-847-640-9241 FAX: 1-847-640-9261 http://www.tanita.com

<Manufacturer>

TANITA® Corporation

1-14-2, Maeno-cho, Itabashi-ku, Tokyo 174-8630 , Japan TEL: +81 (0) 3-3968-7048 FAX: +81 (0) 3-3967-3766 http://www.tanita.co.jp

TANITA Europe B.V.
Hoogoorddreef 56-E,
1101 BE Amsterdam, the Netherlands
TEL: +31-20-560-2970
FAX: +31-20-560-2988
http://www.tanita.eu

TANITA Health Equipment H.K.LTD.
Unit 301-303, Wing On Plaza, 3/F., 62 Mody Rord,
Tsimshatsui East, Kowloon, Hong Kong
TEL: +852-2834-3917
FAX: +852-2838-8667
www.tanita.asia

TANITA India Private Limited
A-502, Mittal Commercia, Off. M.V. Road(Andheri Kurla Road),
Marol, Andheri-East, Mumbai 400 059 INDIA
TEL: +91-22-3192-6107
FAX: +91-22-2859-9143
www.tanita.co.in

TANITA (Shanghai) Trading Co., Ltd.
Room 8005, 877 Huai Hai Zhong Lu, Shanghai,
The People's Republic of China
TEL: +86-21-6474-6803
FAX: +86-21-6474-7901
www.tanita.com.cn

Copyright © 2013 TANITA Corporation. All Rights Reserved.

Feb 2013.TY.C.SC3317601(3)

TANITA®
Monitoring Your Health

ANALIZADOR DE COMPOSICIÓN CORPORAL

SC-331S

Manual de instrucciones

E

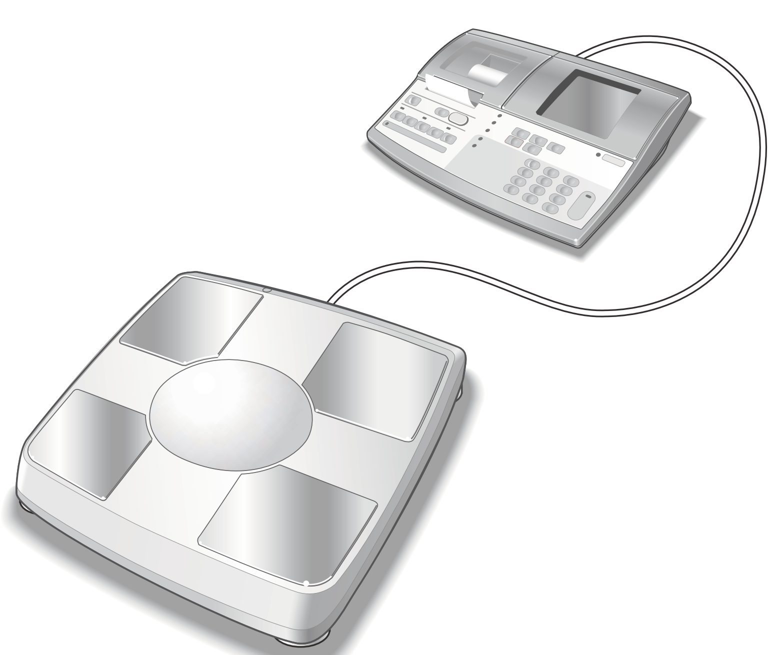

SC-331S
VERSIÓN CON UNIDAD DE VISUALIZACIÓN REMOTA

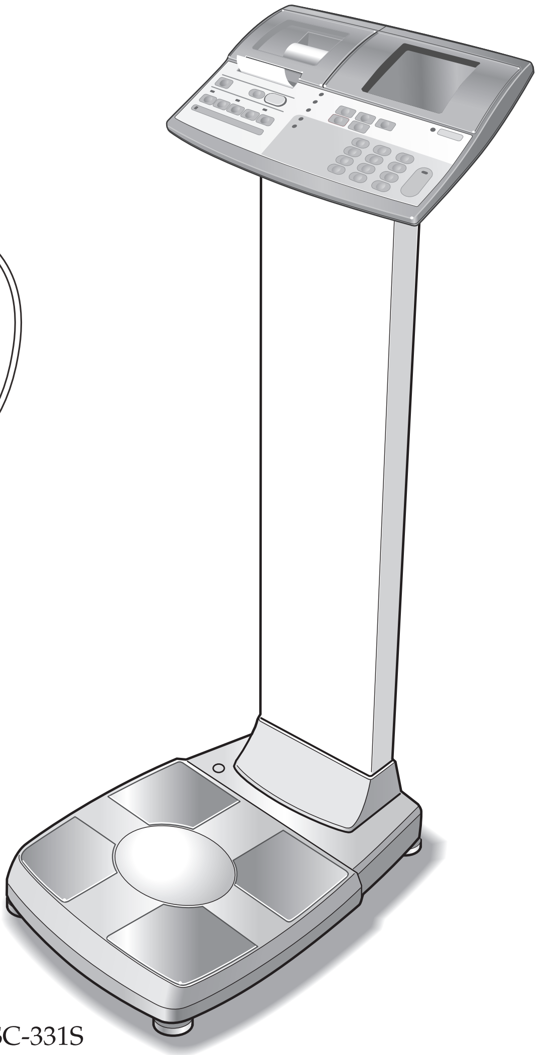

SC-331S
VERSIÓN MONTADA EN COLUMNA

<Condiciones de uso>

Intervalo de temperatura de uso	:	**0°C – 35°C**
Humedad relativa	:	**30% – 80%** (sin condensación)
Altitud máxima	:	**2,000m ASL**
Gama de presión de Medio Ambiente	:	**86kPa - 106kPa**

<Condiciones de almacenamiento>

Intervalo de temperatura ambiental	:	**-10°C – 60°C**
Intervalo de humedad relativa	:	**10% – 90%** (sin condensación)

Para evitar problemas de funcionamiento no almacene este equipo donde haya luz solar directa, cambios significativos de temperatura, riesgo de humedad, mucho polvo, fuego cercano o donde haya riesgo de que reciba choques o vibraciones.

⚠ Lea atentamente este MANUAL DE INSTRUCCIONES y téngalo a mano para posibles consultas.

E

Uso previsto

- Este equipo puede usarse en la detección de ciertas enfermedades de los adultos y condiciones relacionadas con el peso y composición corporal.
- El equipo puede usarse en el control y prevención de afecciones causadas por depósitos excesivos de tejido graso, tales como diabetes, hiperlipidemia, colelitiasis (cálculos biliares) e hígado graso.
- El equipo puede usarse en el control de cambios en la composición corporal de las personas relacionados con la razón de tejido graso a tejido magro.
- El equipo puede usarse para evaluar la efectividad de los programas de nutrición y ejercicios de las personas para la salud y el bienestar físico.
- El analizador de composición corporal Tanita Body Composition Analyzer es indicado para uso en la medición de peso e impedancia y el presupuesto del índice de masa corporal (BMI), el porcentaje de grasa corporal total agua corporal, la masa muscular (esquelétal y lisa) , Tipo de cuerpo, la masa osea, el nivel de grasa visceral con margen saludable, el indice del metabolismo basal (BMR), la edad metabolica, y con el enfoqué al porcentaje de grasa corporal con peso y masa grasa predichos, usando BIA (análisis de la impedancia Bioelectrica).
  El dispositivo es indicado para el uso para niños sanos de 5-17 años y adultos sanos con vidas activas, vidas moderadas, o de vidas inactivas.

Eficacia

1. Este equipo es fácil de usar y no requiere instalaciones especializadas ni experiencia para tomar las mediciones.
2. Las mediciones pueden tomarse en forma rápida y sencilla, causando un mínimo de inconvenientes al paciente durante la medición.

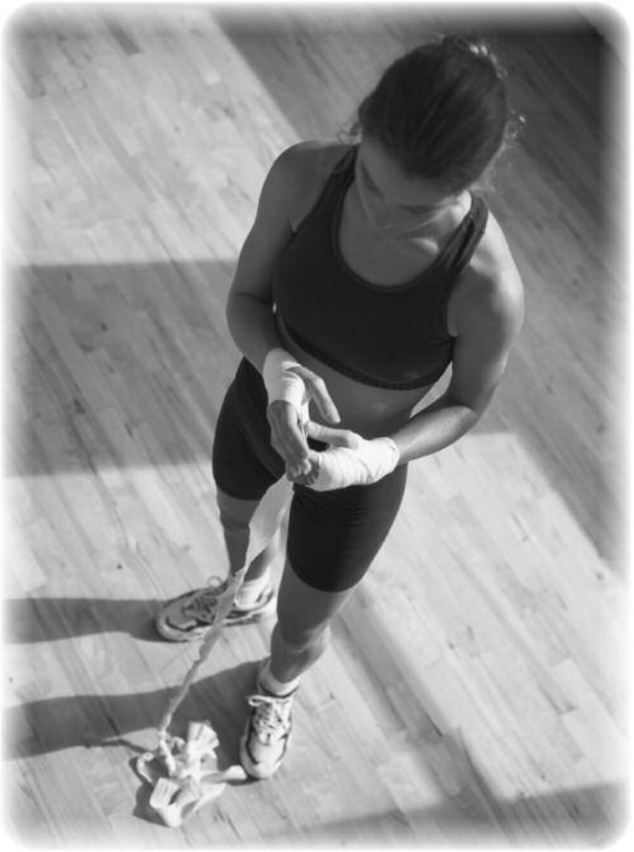

Índice

Antes de utilizar

Notas sobre seguridad ··· **128**

Nombre de los componentes / método de conexión ··· **132**

Preparación ··· **135**

Varios parámetros ··· **136**

Instrucciones de uso

Instrucciones de uso ··· **152**

- Al usar la máquina como analizador de composición corporal ··· 152
- Índice de grasa corporal deseado ··· 162
- Al utilizar la máquina como báscula ··· 163

Diversos criterios ··· **166**

Cuando sea necesario

Daño a la unidad ··· **172**

Conexión a un ordenador personal ··· **174**

Aspectos técnicos ··· **178**

La nueva fórmula de regresión para calcular la Tasa Metabólica Basal (MB) ··· **182**

Especificaciones ··· **184**

E

Para su seguridad

Esta sección explica las medidas de precaución que deben tomarse para evitar lesiones a los usuarios de este dispositivo y a otras personas, así como para evitar daños a la propiedad. Es recomendable que se familiarice con esta información para garantizar la operación segura de este equipo.

**⚠ Advertencia**	**No seguir las instrucciones resaltadas con este símbolo podría producir la muerte o una lesión grave.**
**⚠ Precaución**	**No seguir las instrucciones resaltadas con este símbolo podría producir una lesión o daño a la propiedad.**
⊘	**Este símbolo indica las acciones que están prohibidas.**
❗	**Este símbolo indica las instrucciones que siempre deben seguirse.**

⚠ Advertencia

Prohibido

Este equipo no debe usarse en personas con marcapasos o cualquier otro implante mecánico.
Este equipo envía una pequeña corriente eléctrica a través del cuerpo, la cual podría interferir o causar el mal funcionamiento de los implantes médicos eléctricos, produciendo un grave daño.

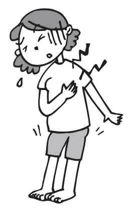

No manipule el enchufe con las manos húmedas.
Esto puede producir un choque eléctrico, incendio o fuga.

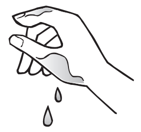

Mantenga este equipo lejos de los gases inflamables y ambientes ricos en oxígeno.

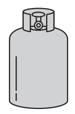

No modifique este equipo de ninguna forma.
Esto puede producir un choque eléctrico o lesión, o puede afectar la precisión del análisis.

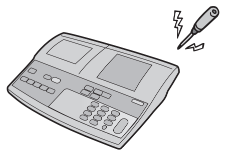

No utilice un adaptador para múltiples enchufes.
Esto puede producir un incendio.

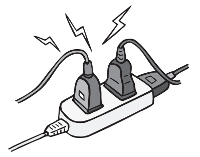

Siempre…

Utilice sólo una toma correctamente cableada.
Sólo los cables originales y los equipos se les permite.

⚠ Precaución

Prohibido

Manténgalo lejos del agua.

Evite usar el equipo en personas con alergias a los metales.
Las reacciones alérgicas pueden ser causadas por el acero inoxidable utilizado en los electrodos de este dispositivo.

No salte en el equipo.

No se apoye en el equipo.
No use este equipo cerca de otros productos que emitan ondas electromagnéticas.

No use este equipo cerca de otros productos que emitan ondas electromagnéticas.

No aplique fuerza a la pantalla.
El panel de la pantalla puede romperse o producir una lesión.

Ayude a las personas con discapacidades.
Otra persona debe realizar las mediciones para las personas con discapacidades que afecten su capacidad de hacerlo ellas mismas.

Siempre…

Asegúrese de limpiar la plataforma de la balanza con un desinfectante adecuado después de cada uso.

Manténgase lejos de la persona durante la medición para evitar un choque eléctrico y para garantizar la precisión.

Controle continuamente tanto a la persona como al equipo para detectar anomalías.
Si se detecta alguna anomalía en la persona o en el equipo, tome la acción adecuada, tal como detener el equipo a la vez que garantiza la seguridad de la persona.

Desenchufe el cable de corriente alterna (CA) cuando mueva el equipo.

La interpretación de los resultados del análisis (por ejemplo, evaluación de las mediciones y formulación de los programas de ejercicios basada en los resultados) debe ser realizada por un profesional.
Las medidas para perder peso y los ejercicios basados en el autoanálisis podrían ser dañinos para su salud. Siga siempre el consejo de un profesional calificado.

Este equipo está designado como un dispositivo de Clase B IT (principalmente para sistemas diseñados para utilizarse en ambientes domésticos) y tiene la certificación de CE (EMC), pero puede afectar a los dispositivos que son sensibles a las ondas electromagnéticas.
Si va a conectar una computadora o dispositivos periféricos a este equipo, utilice los dispositivos cumpliendo con IEC60601-1 (EN60601-1). La energía debe suministrarse desde un transformador de aislamiento para uso médico para los dispositivos IEC60950 (EN60950). Mantenga una distancia de 1,5 m entre las unidades durante la operación. No hacerlo puede producir un choque eléctrico a las personas o un mal funcionamiento.

Para su seguridad

Para realizar una medición precisa

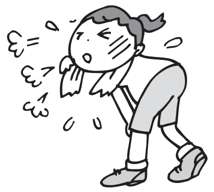

Evite realizar mediciones después de un ejercicio extenuante.

Esto puede producir mediciones inexactas. Realice las mediciones después de un descanso suficiente.

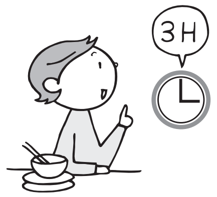

Evite realizar mediciones después de una ingesta de alimentos o líquido en exceso o cuando esté deshidratado.

Esto puede producir mediciones inexactas. Para una mayor precisión, evite usar el equipo directamente después de levantarse. Utilícelo a la misma hora en cada ocasión, por lo menos tres horas después de cada comida.

No tome medidas cuando utilice transmisores, como los teléfonos móviles, que pueden afectar las lecturas.

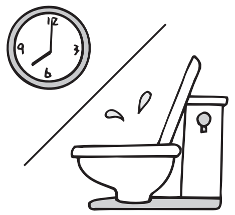

Use el equipo bajo las mismas condiciones y en la misma posición, en la medida de lo posible, para registrar en forma precisa los cambios.

Las lecturas se afectan enormemente por el nivel de hidratación y la posición del cuerpo. Úselo a la misma hora del día cada vez, bajo las mismas condiciones y con la misma posición del cuerpo.

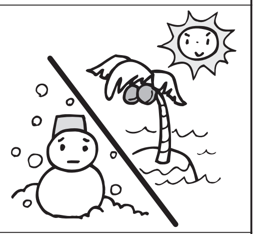

Evite las mediciones en múltiples ubicaciones con grandes diferencias de temperaturas.

Esto puede producir mediciones inexactas. Deje que el equipo descanse por lo menos dos horas antes de usarlo, si ha sido llevado a una nueva ubicación con una diferencia de temperaturas de 20°C o más.

Los pies desnudos deben colocarse correctamente sobre la plataforma de electrodos.

Asegúrese de que las plantas de sus pies estén libres de exceso de suciedad, ya que esto puede actuar como un obstáculo para el paso de la suave corriente eléctrica.

Use una ubicación estable.

Pueden producirse errores en las mediciones si el dispositivo se utiliza en una ubicación inestable.

Mantenimiento programado

Siempre…

TANITA recomienda que cada instalación realice inspecciones periódicas de cada unidad.

1. Revise lo siguiente por lo menos diariamente:
   - Que la unidad esté sobre una superficie estable y nivelada
2. Inspeccione visualmente lo siguiente por lo menos semanalmente:
   - La pantalla, para detectar daños o contaminación
   - Todos los cables, cordones y extremos de los conectores, para detectar daños o contaminación
   - Todas las etiquetas relacionadas con la seguridad, para verificar que estén legibles
   - Todos los accesorios (sensores, electrodos, etc.), para detectar desgaste o daños
   - documentación de la revisión actual de los equipos
3. Inspeccione visualmente lo siguiente por lo menos mensualmente:
   - Los tornillos de montaje en la base

Actualice los ajustes, reemplace los ítems o llame al servicio técnico, según sea necesario, de acuerdo a los resultados de la inspección visual. No utilice la unidad si ve signos de daños. El equipo que está dañado debe ser inspeccionado por personal calificado para verificar su operación adecuada antes de usarlo nuevamente.

El equipo debe ser inspeccionado de acuerdo con la normativa de cada país.

E
Antes de utilizer
(Para su seguridad)

Nombre de los componentes / método de conexión

Versión con unidad de visualización remota

Caja de control

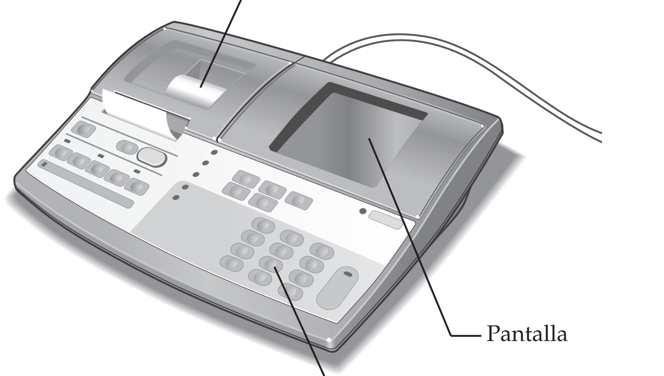

Plataforma

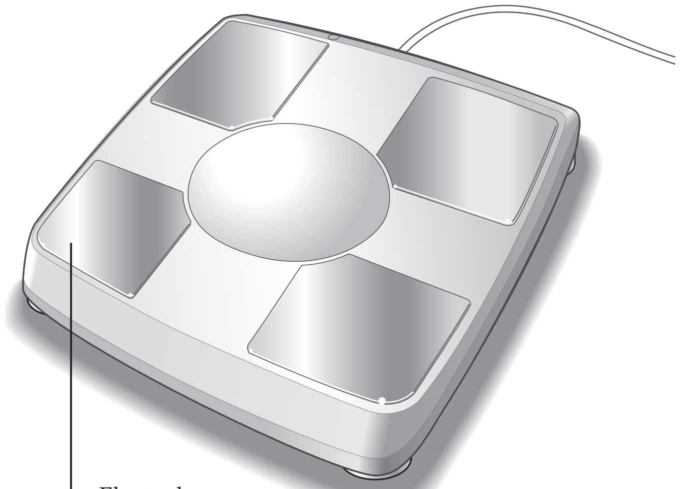

Accesorios

☑ Manual de instrucciones (este manual)
☐ Guía de funcionamiento
☐ D-ROM (controlador USB)
☐ Adaptador de alimentación de CA
☐ Cable de corriente alterna

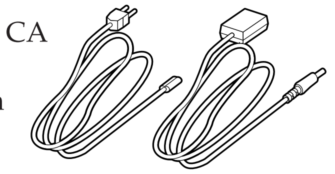

☐ Cuentagotas 1 unidad

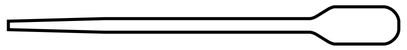

☐ Rollo de papel de impresora
(Papel térmico común; diámetro del rollo: 55 mm, longitud del rollo: aprox. 34 m)
* Para obtener más información, póngase en contacto con el distribuidor que le vendió el producto.

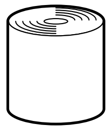

⚠PRECAUCIONES Asegúrese de que la plataforma de medición esté situada sobre una superficie nivelada y estable. Si la plataforma de medición no se encuentra estable debido, por ejemplo, a que no todas las patas se apoyan en el suelo, existe el riesgo de dar un traspiés o de que las mediciones sean inexactas.

Versión montada en columna

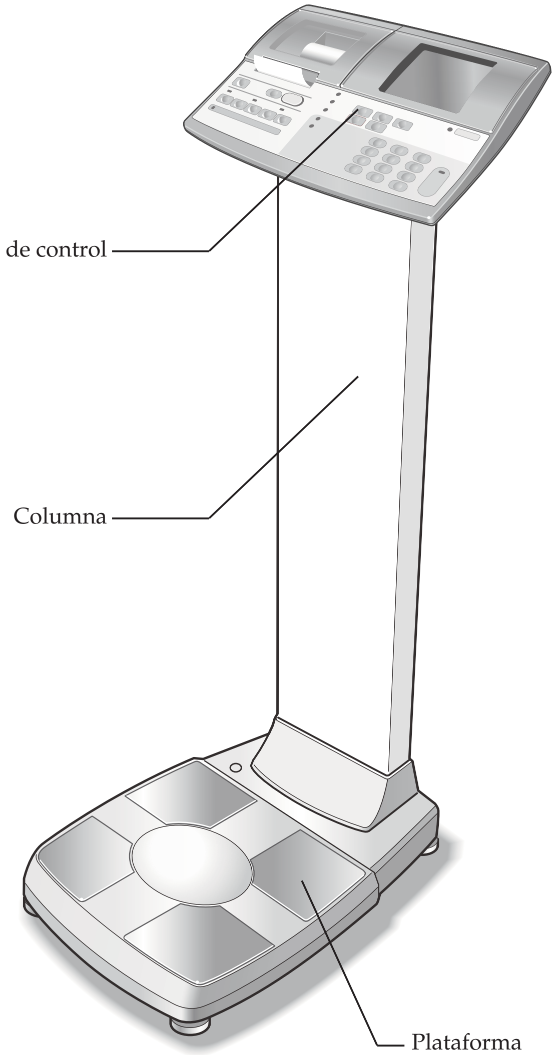

Accesorios

☑ Manual de instrucciones (este manual)
☐ Guía de funcionamiento
☐ D-ROM (controlador USB)
☐ Montaje de guía
☐ Adaptador de alimentación de CA
☐ Cable de corriente alterna

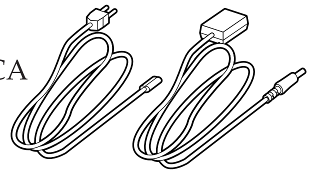

☐ Llave hexagonal (1 unidad)
☐ Pernos de cabeza hexagonal (M5L12) (4 unidades)
☐ Cuentagotas 1 unidad

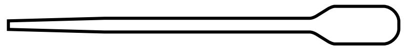

☐ Rollo de papel de impresora
(Papel térmico común; diámetro del rollo: 55 mm, longitud del rollo: aprox. 34 m)
* Para obtener más información, póngase en contacto con el distribuidor que le vendió el producto.

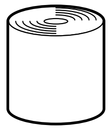

⚠PRECAUCIONES Asegúrese de que la plataforma de medición esté situada sobre una superficie nivelada y estable. Si la plataforma de medición no se encuentra estable debido, por ejemplo, a que no todas las patas se apoyan en el suelo, existe el riesgo de dar un traspiés o de que las mediciones sean inexactas.

Caja de control (nombre de las teclas)

Tecla de alimentación de papel
Para alimentar el papel de la impresora.

Tecla OPEN
Para abrir la tapa de la impresora.

Teclas de selección de tipo corporal
Para seleccionar el tipo corporal.

Teclas de selección de sexo masculino/femenino
Para seleccionar sexo masculino/femenino.

Tecla ON / OFF
Para encender/apagar (ON / OFF) la unidad.

Tecla Enter/Next
Para registrar la información introducida.

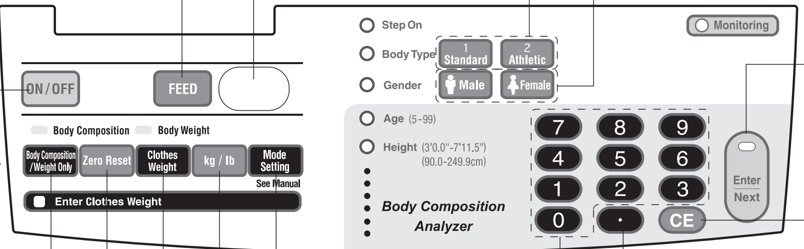

Tecla de selección de modo
Para seleccionar entre el modo de analizador de composición corporal y el modo de sólo peso.

Tecla CE
Para borrar/cancelar la información introducida.

Tecla de configuración
Para ajustar la fecha y la hora, configurar la impresora, etc.

Tecla de punto decimal
Para introducir un punto decimal.

Tecla de puesta a cero
Para poner la báscula a cero.

Teclas numéricas
Para introducir valores numéricos.

Tecla de ajuste de tara preestablecida (peso de la ropa)
Para establecer la tara (peso de la ropa)

Tecla de “kg / lb”
Unidad de cambio

Conexión de enchufes

Parte posterior del controlador

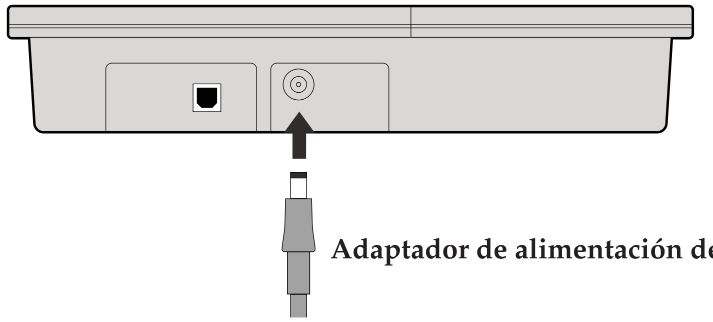

Adaptador de alimentación de CA

⚠ **AVISO IMPORTANTE**
- **Para evitar descargas eléctricas, no conecte o desconecte el enchufe con las manos húmedas.**
- **Para evitar descargas eléctricas, no utilice este equipo cerca del agua.**

⚠ **PRECAUCIONES**
- Para evitar errores de medición, no realice mediciones mientras utiliza equipos que generan ondas de radio, como los teléfonos portátiles.
- Use solamente el adaptador CA original (Modelo: SA25-0535U). Utilizar otros adaptadores diferentes del original puede alterar el funcionamiento o provocar humo o incendios.

Preparació (Instalación del rollo de papel en la impresora)

⚠ **PRECAUCIONES**
- Por favor, cambie el rollo de papel cuando aparezcan líneas rojas en los laterales del papel.
- Por favor, preste especial atención para evitar lesionarse con el borde afilado.
- Por favor, apague la máquina antes de eliminar atascos de papel.

Instalación del rollo de papel en la impresora

1 **Pulse ON/OFF para encender la unidad.**
- Después de que se encienden todas las lámparas, aparece el número de modelo 331 y luego se visualiza 0.0kg.

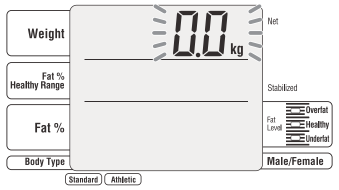

2 **(1) Pulse OPEN (abrir).**
(2) Abra la tapa de la impresora.

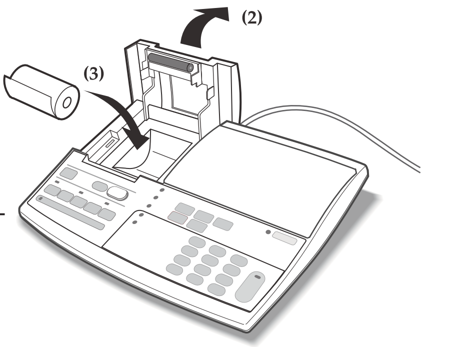

3 **(3) Instale el rollo de papel en la impresora.**
- Quite el adhesivo del rollo de papel y desenróllelo aproximadamente 10 cm.

4 **Cierre la tapa de la impresora.**

⚠
- Si se visualiza C.OPEn
⇨La tapa de la impresora está abierta; asegúrese de cerrarla correctamente (☞ página 173).

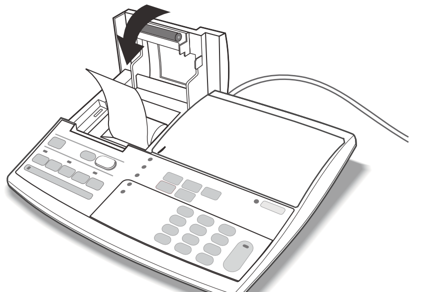

5 **Pulse la tecla FEED (papel) y corte el exceso de papel.**

⚠
- Si la opción de corte automático está ajustada a “OFF,” el corte automático no se lleva a cabo (☞ página 140).

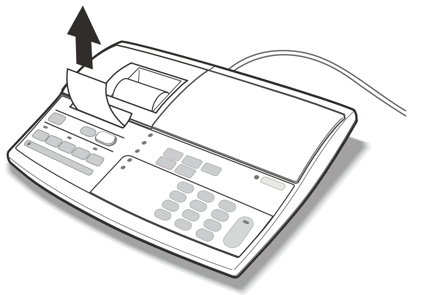

6 **Instalación completa.**

Varios parámetros (Métodos de ajuste)

Abra la pantalla de selección del ajuste.

1 Pulse ON/OFF para encender la unidad.

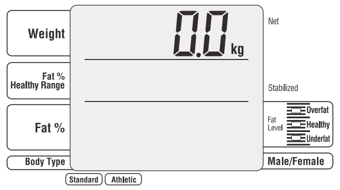

- Después de que se encienden todas las lámparas, aparece el número de modelo y luego se visualiza 0.0 kg.

2 Pulse Mode Setting.

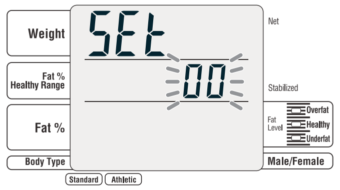

- Aparece la pantalla de introducción del ajuste.

Nota
- Los elementos 39 – 67 son los ajustes para imprimir (☞ página 148).
- Una vez realizados todos los ajustes:
  ⇨Pulse Mode Setting en la pantalla “Selección del ajuste” (**vuelve a aparecer la pantalla de introducción de tara**).

Elementos de ajuste

1	Fecha y hora (☞ página 138)
2	Cantidad de hojas a imprimir<br>Modo de composición corporal (☞ página 140)
3	Cantidad de hojas a imprimir<br>Modo de sólo peso (☞ página 139)
4	Corte automático del papel de la impresora<br>(☞ página 140)
5	Señal audible (☞ página 140)
6	Visualización del margen de % de grasasaludable<br>(☞ página 141)
7	Núm. de ID (☞ página 141)
8	Flujo de medición (☞ página 142)
9	Modo atlético (☞ página 142)
10*	Introducción de unidad de estatura (☞ página 143)
11	Tiempo de determinación automática (☞ página 143)
17	Modalità Lottatore (☞ página 144)
18	Porcentaje objetivo de grasa corporal (☞ página 145)
19	Seleccionar idioma (☞ página 145)
20	Preajuste de elementos a imprimir (☞ página 146)

* Inválido:lb

Activación / desactivación de ajustes de impresión (☞ página 146)

39	Logotipo de TANITA
40	Nombre de categoría
41	Fecha
42	Número de serie
43	Memoria
44	Núm. de ID
45	Masa de grasa
46	Masa libre de grasa
47	Masa muscular
48	Agua corporal total
49	% de agua corporal total
50	Masa ósea
51	Tasa de metabolismo basal (TMB)
52	Edad metabólica
53	Índice de grasa visceral
54	IMC (índice de masa corporal)
56	Peso corporal ideal
57	Grado de obesidad
58	Rango deseable de % de grasa corporal
59	Gráfico de % de grasa %
60	Gráfico de IMC
61	Gráfico de índice de grasa visceral
62	Gráfico de masa muscular
63	Gráfico de TMB
64	Índice de físico

Nota

*Al realizar diversos ajustes de forma continua,
⇨pulse cada número para registrarlo.
*La máquina retendrá la información introducida hasta la próxima vez que se modifique.

*Una vez realizados todos los ajustes:
⇨Pulse Mode Setting en la pantalla “selección del ajuste” (**vuelve a aparecer la pantalla de introducción de tara**).

Registre la fecha y la hora (viene de la página 136).

3 Pulse 1 y luego pulse Enter / Next.

• Aparece la pantalla de ajuste de "fecha y hora".

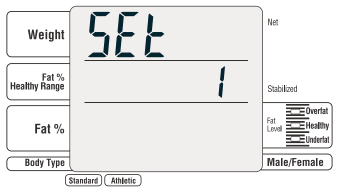

4 Introduzca la fecha y la hora.

• Introduzca el año, mes, día, horas y minutos con 2 dígitos, en ese orden.

Ejemplo: Para introducir: 9:47 am, 21 de enero, 2009

0 9 0 1 2 1 0 9 4 7

Año 2009 Enero 21st

Para introducir las 6:00 pm, pulse 1 8

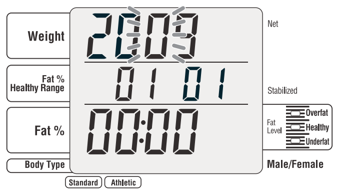

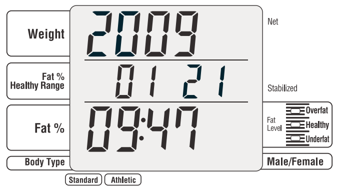

Nota
- Para introducir un número de 1 dígito (0 – 9), primero pulse 0.
- Para corregir el valor introducido o cancelar la entrada:
  ⇨pulse CE (se borra la información introducida).
- Para dejar de introducir durante el proceso,
  ⇨pulse Enter / Next.

5 Después de introducir todos los elementos, pulse Enter / Next.

• La máquina vuelve a mostrar la pantalla "selección del ajuste".

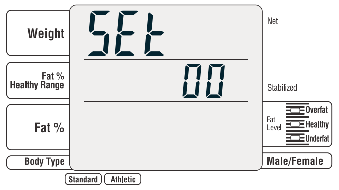

Nota
*Al realizar diversos ajustes de forma continua,
⇨pulse cada número para registrarlo.
*La máquina retendrá la información introducida hasta la próxima vez que se modifique.

Establezca el número de páginas a imprimir para el analizador de composición corporal (viene de la página 136).

3 Pulse 2 y luego pulse Enter / Next.

• Aparece la pantalla de ajuste del "número de hojas a imprimir para el monitor de composición corporal".

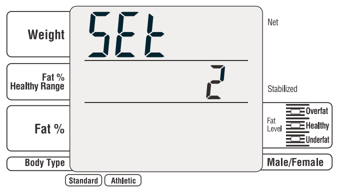

4 Introduzca el número de hojas a imprimir.

Nota
- El ajuste predeterminado es "1" (margen de introducción: 0 – 3).
- Si el número de hojas a imprimir se ajusta a "0" tanto para el monitor de composición corporal como para la báscula, la tecla FEED (papel) no funcionará.
- Para corregir el valor introducido o cancelar la entrada:
  ⇨pulse CE (se borra la información introducida).

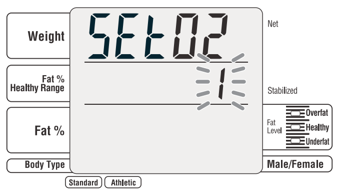

5 Después de introducir el valor numérico, pulse Enter / Next.

• La máquina vuelve a mostrar la pantalla "selección del ajuste".

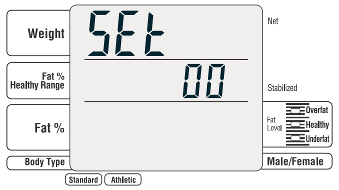

Ajuste el número de hojas a imprimir para la báscula (viene de la página 136).

3 Pulse 3 y luego pulse Enter / Next.

• Aparece la pantalla de ajuste del "número de hojas a imprimir para la bascula".

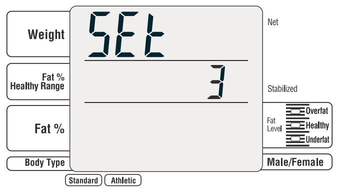

4 Introduzca el número de hojas a imprimir.

Nota
- El ajuste predeterminado es "1" (margen de introducción: 0 – 3).
- Si el número de hojas a imprimir se ajusta a "0" tanto para el monitor de composición corporal como para la báscula, la tecla FEED (papel) no funcionará.
- Para corregir el valor introducido o cancelar la entrada:
  ⇨pulse CE (se borra la información introducida).

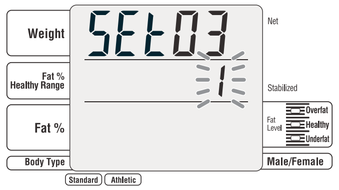

5 Después de introducir el valor numérico, Enter / Next.

• La máquina vuelve a mostrar la pantalla "selección del ajuste".

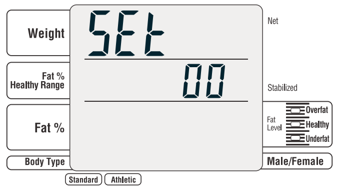

*Una vez realizados todos los ajustes:
⇨Pulse Mode Setting en la pantalla "selección del ajuste" (**vuelve a aparecer la pantalla de introducción de tara**).

Varios parámetros (Métodos de ajuste (continuación))

Seleccione ON / OFF para la opción de corte automático del papel de la impresora (viene de la página 136).

3 **Pulse** 4 **y luego pulse** Enter / Next**.**

- Aparece la pantalla de selección "corte automático ON / OFF."

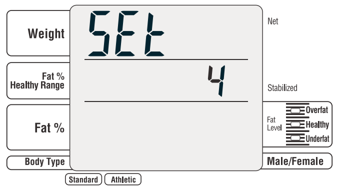

4 **Seleccione ON u OFF para la opción de corte automático.**

Nota
- El ajuste predeterminado es "1.on." ("1. on" = válido, "0. off" = no válido)
- Para corregir el valor introducido o cancelar la entrada:
  ⇨pulse CE (se borra la información introducida).

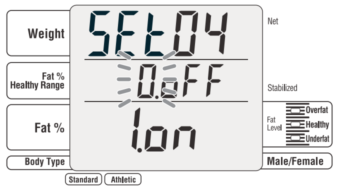

5 **Después de introducir el valor numérico,** Enter / Next**.**

- La máquina vuelve a mostrar la pantalla "selección del ajuste".

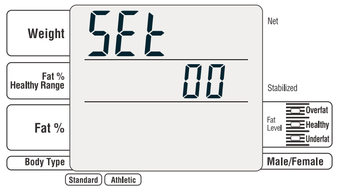

Seleccione ON u OFF para la señal audible (viene de la página 136).

3 **Pulse** 5 **y luego pulse** Enter / Next**.**

- Aparece la pantalla de selección "señal audible ON / OFF".

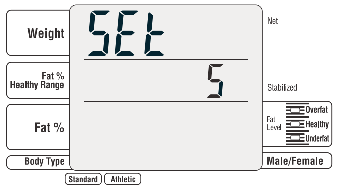

4 **Seleccione ON u OFF para la señal audible.**

Nota
- El ajuste predeterminado es "1.on." ("1. on" = válido, "0. off" = no válido)
- Para corregir el valor introducido o cancelar la entrada:
  ⇨pulse CE (se borra la información introducida).

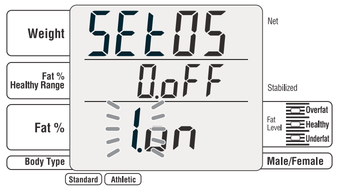

5 **Después de introducir el valor numérico, pulse** Enter / Next**.**

- La máquina vuelve a mostrar la pantalla "selección del ajuste".

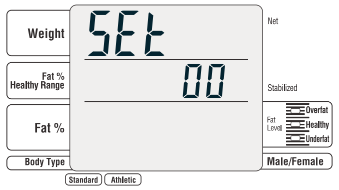

Nota

*Al realizar diversos ajustes de forma continua,
⇨pulse cada número para registrarlo.
*La máquina retendrá la información introducida hasta la próxima vez que se modifique.

Seleccione ON / OFF para la opción Visualización del margen de % de grasa saludable (viene de la página 136).

3 **Pulse** 6 **y luego pulse** Enter / Next**.**

- Aparece la pantalla de selección "ON o OFF para la opción Visualización del margen de % de grasa saludable".

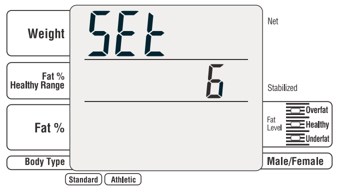

4 **Seleccione ON u OFF para la opción "Visualización del margen de % de grasa condición" al utilizar el analizador de composición corporal.**

Nota
- El ajuste predeterminado es "1.on." ("1. on" = válido, "0. off" = no válido)
- Para corregir el valor introducido o cancelar la entrada:
  ⇨pulse CE (se borra la información introducida).

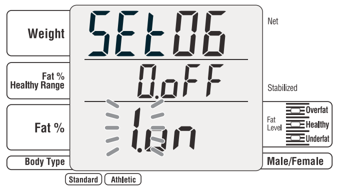

5 **Después de introducir el valor numérico,** Enter / Next**.**

- La máquina vuelve a mostrar la pantalla "selección del ajuste".

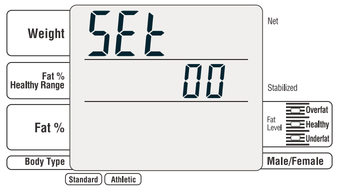

Realice el ajuste con/sin un ID (viene de la página 136).

3 **Pulse** 7 **y luego pulse** Enter / Next**.**

- Aparece la pantalla de ajuste "con o sin ID".

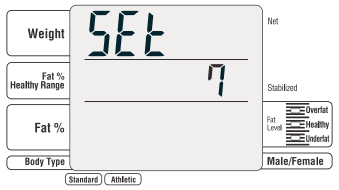

4 **Realice el ajuste con o sin un ID.**

Nota
- El ajuste predeterminado es "0. off." ("1. on" = con ID, "0. off" = sin ID)
- Para corregir el valor introducido o cancelar la entrada:
  ⇨pulse CE (se borra la información introducida).

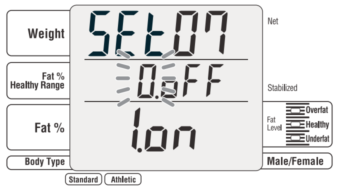

5 **Después de introducir el valor numérico,** Enter / Next**.**

- La máquina vuelve a mostrar la pantalla "selección del ajuste".

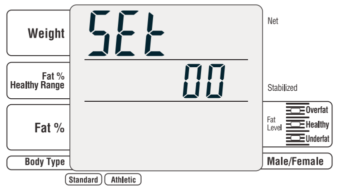

*Una vez realizados todos los ajustes:
⇨Pulse Mode Setting en la pantalla "selección del ajuste" (**vuelve a aparecer la pantalla de introducción de tara**).

Varios parámetros (Métodos de ajuste (continuación))

Seleccione el flujo de medición (viene de la página 136).

3 Pulse 8 y luego pulse Enter / Next.

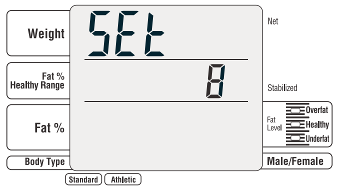

- Aparece la pantalla de selección “ON u OFF para el modo de un solo paso”.

Nota
- El modo de un solo paso es
  ⇨un modo que se utiliza para medir el peso corporal después de introducir información personal.

4 Seleccione ON u OFF para el modo de un solo paso al utilizar el analizador de composición corporal.

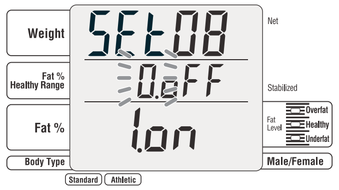

Nota
- El ajuste predeterminado es “0. off.” (“1. on” = válido, “0. off” = no válido)
- Para corregir el valor introducido o cancelar la entrada:
  ⇨pulse CE (se borra la información introducida).

5 Después de introducir el valor numérico, pulse Enter / Next.

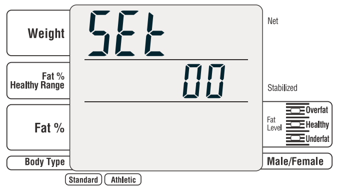

- La máquina vuelve a mostrar la pantalla “selección del ajuste”.

Seleccione ON / OFF para la selección de modo atlético (viene de la página 136).

3 Pulse 9 y luego pulse Enter / Next.

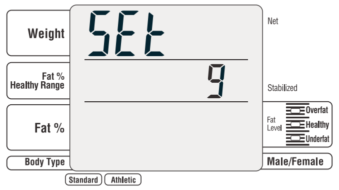

- Aparece la pantalla de selección “Seleccione ON / OFF para la selección del modo atlético”.

4 Seleccione ON u OFF para la selección de modo atlético al utilizar el analizador de composición corporal.

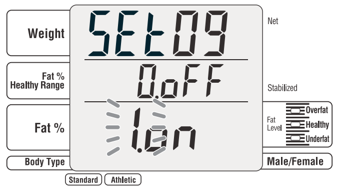

Nota
- El ajuste predeterminado es “1.on.” (“1. on” = válido, “0. off” = no válido)
- Para corregir el valor introducido o cancelar la entrada:
  ⇨pulse CE (se borra la información introducida).

5 Después de introducir el valor numérico, pulse Enter / Next.

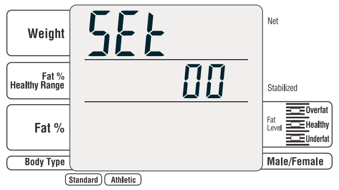

- La máquina vuelve a mostrar la pantalla “selección del ajuste”.

Nota
*Al realizar diversos ajustes de forma continua,
⇨pulse cada número para registrarlo.
*La máquina retendrá la información introducida hasta la próxima vez que se modifique.

Seleccione la unidad de introducción de estatura (viene de la página 136).

3 Pulse 1 0 y luego pulse Enter / Next.

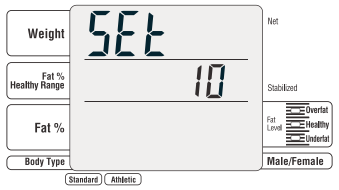

- Aparece la pantalla de selección “introduzca unidad de peso”.
- * Inválido:lb

4 Seleccione la unidad de introducción de estatura.

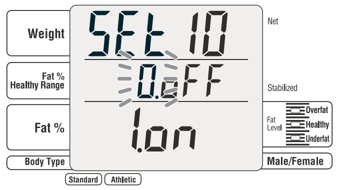

Nota
- El ajuste predeterminado es “0. off.”
  0. off: establece una unidad de introducción de estatura de 0,1 cm
  1. on: establece una unidad de introducción de estatura de 1 cm
- Para corregir el valor introducido o cancelar la entrada:
  ⇨pulse CE (se borra la información introducida).

5 Después de introducir el valor numérico, pulse Enter / Next.

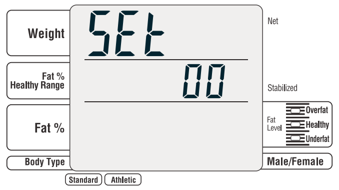

- La máquina vuelve a mostrar la pantalla “selección del ajuste”.

Establezca el tiempo de determinación automática durante la introducción de información (viene de la página 136).

3 Pulse 1 1 y luego pulse Enter / Next.

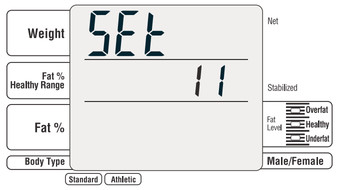

- Aparece la pantalla de selección “tiempo de determinación automática durante la introducción de información”.

Nota
- La función de determinación automática durante la introducción de información es:
  ⇨una función que determina automáticamente el valor introducido, incluso si no se pulsa de manera inmediata Enter / Next, después de introducir un valor numérico.

4 Establezca el tiempo de determinación automática durante la introducción de información.

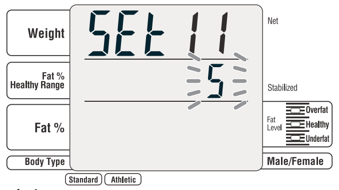

Nota
- El ajuste predeterminado es 5 segundos (“5”). (margen de introducción: 0 – 9).
  *Si se selecciona “0”, los valores no se determinan automáticamente.
- Para corregir el valor introducido o cancelar la entrada:
  ⇨pulse CE (se borra la información introducida).

5 Después de introducir el valor numérico, pulse Enter / Next.

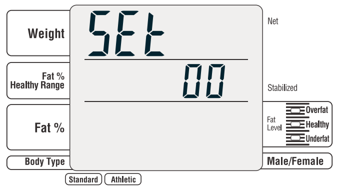

- La máquina vuelve a mostrar la pantalla “selección del ajuste”.

*Una vez realizados todos los ajustes:
⇨Pulse Mode Setting en la pantalla “selección del ajuste” (**vuelve a aparecer la pantalla de introducción de tara**).

Varios parámetros (Métodos de ajuste (continuación))

Establecimiento del modo de luchador (viene de la página 136).

3 Pulse [1] [7] y luego pulse [Enter / Next].

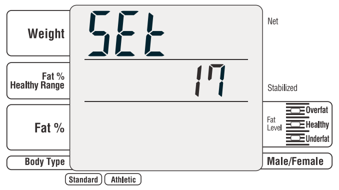

4 Establecimiento del modo de luchador

Nota
- El valor predeterminado es 0.off
- 0: el modo esta desactivado
- 5: Establecimiento del 5% en el modo de luchador
- 7: Establecimiento del 7% en el modo de luchador
- Lorsque vous activez le “mode Lutteur”, la TBF-300A calcule automatiquement le poids minimum du lutteur pour un pourcentage de masse grasse minimum prédéterminé. La prise de position de 1996, de l’ACSM, intitulée “Perte de poids chez les lutteurs”, adoptée par la NCAA, recommande les pourcentages de masse grasse MINIMUMS suivants:
  5% for Collegiate Athletes (male)
  7% for High School Athletes (male)

⚠ L’objectif de pourcentage de masse grasse est complètement indépendant des calculs du poids de lutte minimum (POIDS min).
Exemple :
Vous pouvez régler l’objectif de pourcentage de masse grasse sur 15%, même si le POIDS min est calculé pour une masse grasse minimum prédéterminée de 5 ou de 7%.

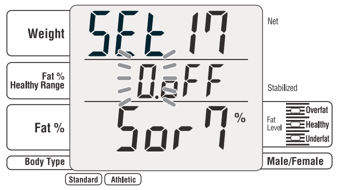

5 Después de introducir el valor numérico, [Enter / Next].
- La máquina vuelve a mostrar la pantalla “selección del ajuste”.

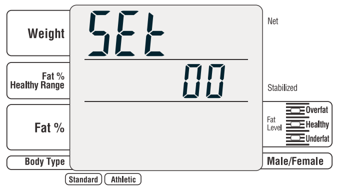

Nota
*Al realizar diversos ajustes de forma continua,
⇨pulse cada número para registrarlo.
*La máquina retendrá la información introducida hasta la próxima vez que se modifique.

Seleccione ON /OFF de la ratio de grasa corporal objetivo (viene de la página 136).

3 Pulse [1] [8] y luego pulse [Enter / Next].

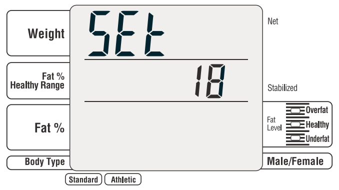

4 Seleccione ON u OFF de la ratio de grasa corporal objetivo

Nota
- El valor por defecto es “0.off” (“1.on” para válido, “0.off” para inválido)
- Para corregir el valor introducido o cancelar la entrada:
  ⇨pulse [CE] (se borra la información introducida).

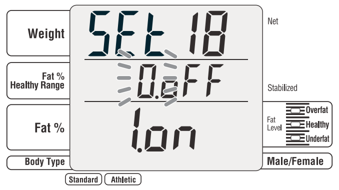

5 Después de introducir el valor numérico, pulse [Enter / Next].
- La máquina vuelve a mostrar la pantalla “selección del ajuste”.

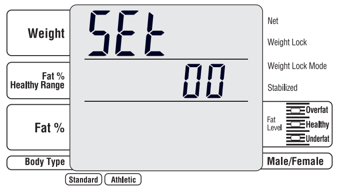

Determine el lenguaje de impresión (viene de la página 136).

3 Pulse [1] [9] y luego pulse [Enter / Next].

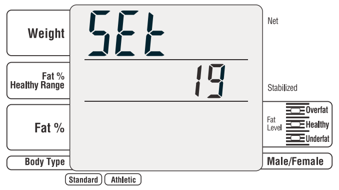

4 Determine el lenguaje de impresión

Nota
- 1. Inglés 2. Francés 3. Alemán
  4. Italiano 5. Español 6. Holandés
- Para corregir el valor introducido o cancelar la entrada:
  ⇨pulse [CE] (se borra la información introducida).

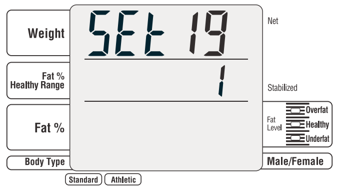

5 Después de introducir el valor numérico, pulse [Enter / Next].
- La máquina vuelve a mostrar la pantalla “selección del ajuste”.

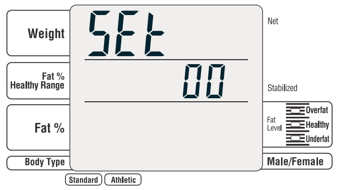

*Una vez realizados todos los ajustes:
⇨Pulse [Mode Setting] en la pantalla “selección del ajuste” (**vuelve a aparecer la pantalla de introducción de tara**).

Establezca el preajuste de elementos a imprimir (viene de la página 234).

3 Pulse (2)(0) y luego pulse (Enter / Next).

• Aparece la pantalla de selección “print out preset”.

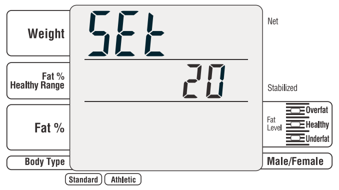

4 Establezca el preajuste de elementos a imprimir.

• Realice el ajuste con (1) – (3).

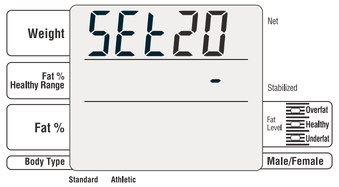

Nota
- La función de preajuste de elementos a imprimir es ⇨una función que permite ajustar los elementos de impresión del patrón 1 – 3 a ON automáticamente (☞página 147). El ajuste predeterminado es “1” (patrón 1).
- Cuando se cambia el ajuste ON u OFF de los elementos de impresión con “selección de los elementos de impresión” después de preestablecer los elementos a imprimir (☞página 150), el último estado establecido es válido.

5 Después de introducir el valor numérico, pulse (Enter / Next).

• La máquina vuelve a mostrar la pantalla “selección del ajuste”.

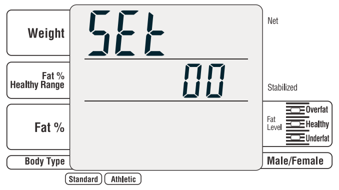

Nota
*Al realizar diversos ajustes de forma continua,
⇨pulse cada número para registrarlo.
*La máquina retendrá la información introducida hasta la próxima vez que se modifique.

[Lista de elementos de impresión]

Elemento de impresión	Monitor de composición de estructura corporal									Báscula
	(1) Modelo 1			(2) Modelo 2			(3) Modelo 3			
Tipo de estructura corporal	Normal	Atlètico	Niño	Normal	Atlètico	Niño	Normal	Atlètico	Niño	
Logotipo de TANITA										✓
Nombre de categoría										
Fecha										
Número de serie										
Memoria										✓
Núm. de ID										
Masa de grasa										
Masa libre de grasa										
Masa muscular										
Agua corporal total										
% de agua corporal total										
Masa ósea										
Tasa de metabolismo basal (TMB)										
Edad metabólica										
Índice de grasa visceral										
IMC (índice de masa corporal)										
Peso corporal ideal										
Grado de obesidad										
Rango deseable de % de grasa corporal										
Mode Lutteur										
Gráfico de % de grasa %										
Gráfico de IMC										
Gráfico de índice de grasa visceral										
Gráfico de masa muscular										
Gráfico de TMB										
Índice de físico										

- Los elementos marcados con “✓” se imprimen.
- Se pueden seleccionar los elementos marcados con para imprimirlos (☞página 148)
  * Para ver un ejemplo de impresión de elementos preestablecidos, consulte la página 148.

*Una vez realizados todos los ajustes:
⇨Pulse (Mode Setting) en la pantalla “selección del ajuste” (**vuelve a aparecer la pantalla de introducción de tara**).

Varios parámetros (Métodos de ajuste (continuación))

[Al seleccionar el patrón de elementos de impresión “1”]

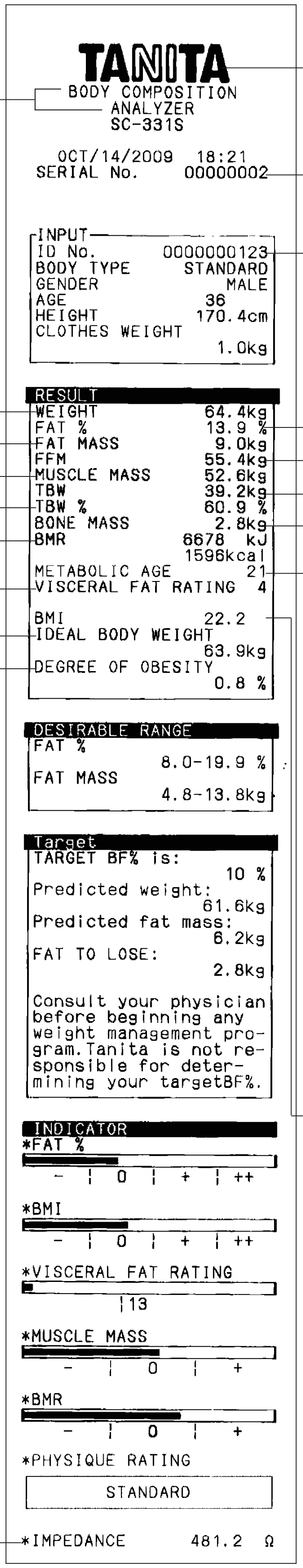
TANITA
BODY COMPOSITION ANALYZER SC-331S
OCT/14/2009 18:21
SERIAL No. 00000002
INPUT
ID No. 0000000123
BODY TYPE STANDARD
GENDER MALE
AGE 36
HEIGHT 170.4cm
CLOTHES WEIGHT 1.0kg
RESULT
WEIGHT 64.4kg
FAT % 13.9 %
FAT MASS 9.0kg
FFM 55.4kg
MUSCLE MASS 52.6kg
TBW 39.2kg
TBW % 60.9 %
BONE MASS 2.8kg
BMR 6678 kJ
1596kcal
METABOLIC AGE 21
VISCERAL FAT RATING 4
BMI 22.2
IDEAL BODY WEIGHT 63.9kg
DEGREE OF OBESITY 0.8 %
DESIRABLE RANGE
FAT % 8.0-19.9 %
FAT MASS 4.8-13.8kg
Target
TARGET BF% is: 10 %
Predicted weight: 61.6kg
Predicted fat mass: 6.2kg
FAT TO LOSE: 2.8kg
Consult your physician before beginning any weight management program. Tanita is not responsible for determining your targetBF%.
INDICATOR
*FAT %
- 0 + ++
*BMI
- 0 + ++
*VISCERAL FAT RATING
13
*MUSCLE MASS
- 0 +
*BMR
- 0 +
*PHYSIQUE RATING
STANDARD
*IMPEDANCE 481.2 Ω

Nombre de categoría

Peso
- Peso corporal medido.

Masa Grasa
- Peso total de la masa de grasa contenida en el cuerpo.

Masa Muscular
- Masa muscular magra libre de hueso (LTM)

% Agua Total

MB*
- El Índice de metabolismo basal representa la cantidad total de energía gastada por el cuerpo para mantener las funciones normales en reposo como la respiración y la circulación.

Nivel de Grasa Visceral*
- El nivel de grasa visceral indica la cantidad de grasa visceral.

Peso Ideale*
- El peso corporal ideal es un valor para el cual el IMC es 22.

Grado de Obesidad*
- Se calcula como (peso) – (peso estándar) / (peso estándar) × 100.

Impedancia
- Impedancia (No tiene efecto sobre la evaluación de los resultados de medición).

Logotipo

Número de Serie
- La selección por defecto es 00000001. Añade 1 por cada medición que realiza.

ID
- Cuando se introduce con un Identificador de usuario, éste se imprime. (Por defecto se imprime sin ID).

Masa Grasa %
- El porcentaje de grasa es la grasa corporal en proporción con el peso corporal.

Masa Magra
- La masa libre de grasa se compone de músculo, huesos, tejido, agua y otras masas libres de grasa en el cuerpo.

Agua Total
- El agua corporal total es la cantidad de agua retenida en el cuerpo. Se dice que el ACT corresponde a aproximadamente el 50% - 70% del peso corporal total. Generalmente, los hombres tienden a tener un mayor peso de agua que las mujeres debido a que poseen una mayor masa muscular.

Masa Ósea*
- La cantidad de mineral óseo contenida en el hueso completo.

Edad Metabólica*
- Se considera que la edad metabólica es baja (joven) cuando la masa muscular es grande y la TMB es elevada.

BMI
- Se calcula con la fórmula “peso (kg) / altura (m)²”
- Ámbito deseable 18,5 – 24,9

* El valor estándar es para el modo Estándar. En el caso del modo Atlético, el valor estándar es sólo una referencia. Y para los menores de 17 años, sólo se muestra como valor estándar el porcentaje de grasa corporal. La masa muscular, la cantidad total de agua en el organismo y la masa ósea estimada para los menores de 17 años son sólo valores de referencia.

***18 - 99 anos**

⚠ Consulte a su médico antes de comenzar cualquier programa de control de peso. Tanita no se hace responsable de la determinación del objetivo de proporción de grasa corporal.

[Al seleccionar el patrón de elementos de impresión “2”]

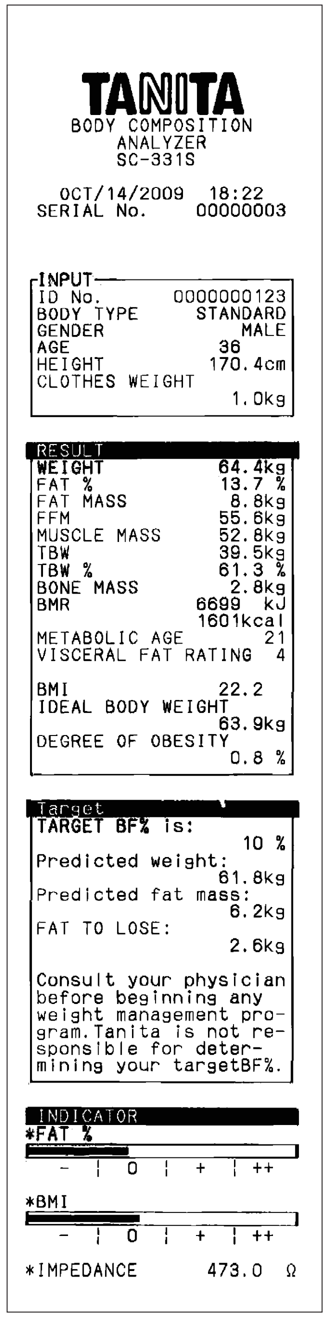
TANITA
BODY COMPOSITION ANALYZER SC-331S
OCT/14/2009 18:22
SERIAL No. 00000003
INPUT
ID No. 0000000123
BODY TYPE STANDARD
GENDER MALE
AGE 36
HEIGHT 170.4cm
CLOTHES WEIGHT 1.0kg
RESULT
WEIGHT 64.4kg
FAT % 13.7 %
FAT MASS 8.8kg
FFM 55.6kg
MUSCLE MASS 52.8kg
TBW 39.5kg
TBW % 61.3 %
BONE MASS 2.8kg
BMR 6699 kJ
1601kcal
METABOLIC AGE 21
VISCERAL FAT RATING 4
BMI 22.2
IDEAL BODY WEIGHT 63.9kg
DEGREE OF OBESITY 0.8 %
Target
TARGET BF% is: 10 %
Predicted weight: 61.8kg
Predicted fat mass: 6.2kg
FAT TO LOSE: 2.6kg
Consult your physician before beginning any weight management program. Tanita is not responsible for determining your targetBF%.
INDICATOR
*FAT %
- 0 + ++
*BMI
- 0 + ++
*IMPEDANCE 473.0 Ω

[Al seleccionar el patrón de elementos de impresión “3”]

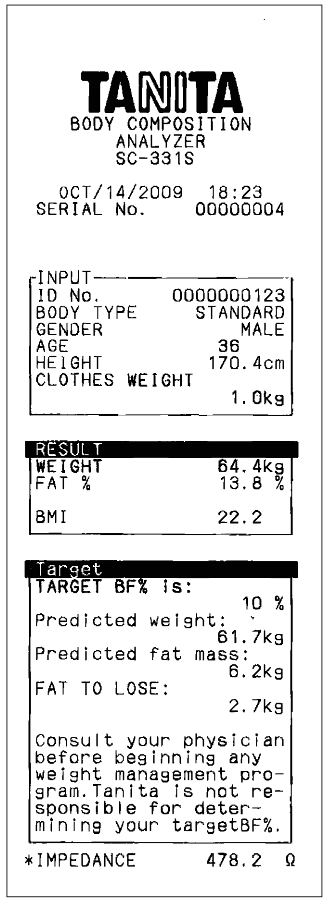
TANITA
BODY COMPOSITION ANALYZER SC-331S
OCT/14/2009 18:23
SERIAL No. 00000004
INPUT
ID No. 0000000123
BODY TYPE STANDARD
GENDER MALE
AGE 36
HEIGHT 170.4cm
CLOTHES WEIGHT 1.0kg
RESULT
WEIGHT 64.4kg
FAT % 13.8 %
BMI 22.2
Target
TARGET BF% is: 10 %
Predicted weight: 61.7kg
Predicted fat mass: 6.2kg
FAT TO LOSE: 2.7kg
Consult your physician before beginning any weight management program. Tanita is not responsible for determining your targetBF%.
*IMPEDANCE 478.2 Ω

⚠ Consulte a su médico antes de comenzar cualquier programa de control de peso. Tanita no se hace responsable de la determinación del objetivo de proporción de grasa corporal.

Varios parámetros (Métodos de ajuste (continuación))

Cuando se selecciona la activación del ajuste del logotipo (viene de la página 136).

3 Seleccione el número que desea introducir con las teclas numéricas (3 9 – 6 4) y, a continuación, pulse Enter / Next .

<In the case of selecting 39>

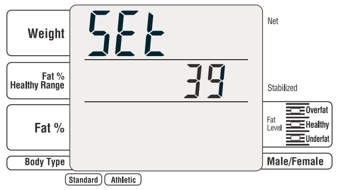

• Aparece la pantalla de ajuste.

Nota

• Consulte la página siguiente para obtener información sobre el número de ajuste de cada elemento.
• Cuando se selecciona impresión de elementos preestablecidos, después de establecer los elementos a imprimir (☞ página 148), los elementos a imprimir pueden cambiarse automáticamente. Compruebe la “Lista de elementos de impresión” (☞ página 147).

4 Seleccione ON u OFF para el elemento a imprimir.

‹ In the case of selecting the logo setting on. ›

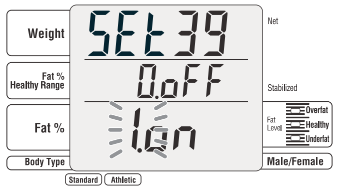

Nota

• “0. off” = no se imprime, “1. on” = se imprime.
• Para corregir el valor introducido o cancelar la entrada:
⇨pulse CE (se borra la información introducida).

5 Después de introducir el valor numérico, Enter / Next .

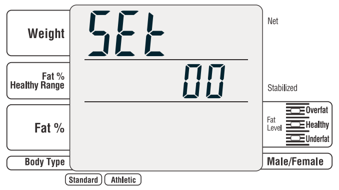

• La máquina vuelve a mostrar la pantalla “selección del ajuste”.

Impresión de la configuración de elementos ON / OFF

39	Logotipo de TANITA
40	Nombre de categoría
41	Fecha
42	Número de serie
43	Memoria
44	Núm. de ID
45	Masa de grasa
46	Masa libre de grasa
47	Masa muscular
48	Agua corporal total
49	% de agua corporal total
50	Masa ósea
51	Tasa de metabolismo basal (TMB)

52	Edad metabólica
53	Índice de grasa visceral
54	IMC (índice de masa corporal)
56	Peso corporal ideal
57	Grado de obesidad
58	Rango deseable de % de grasa corporal
59	Gráfico de % de grasa %
60	Gráfico de IMC
61	Gráfico de índice de grasa visceral
62	Gráfico de masa muscular
63	Gráfico de TMB
64	Índice de físico

Nota

*Al realizar diversos ajustes de forma continua,
⇨pulse cada número para registrarlo.
*La máquina retendrá la información introducida hasta la próxima vez que se modifique.

*Una vez realizados todos los ajustes:
⇨Pulse Mode Setting en la pantalla “selección del ajuste” (**vuelve a aparecer la pantalla de introducción de tara**).

Instrucciones de uso (al usar la máquina como analizador de composición corporal)

Explica el procedimiento cuando la impresora está encendida. Observe que el display puede ser diferente si el número de impresiones se configura en (0).

- No limpie el equipo con productos químicos corrosivos (gasolina, limpiadores, etc). Utilice un detergente neutro para limpiarlo.
- Cuando se haya trasladado el equipo a un lugar con una diferencia de temperatura de 20ºC o más, espere al menos dos horas antes de utilizarlo.
- Al tomar medidas, mantenga alejada de la unidad a cualquier persona que utilice transmisores como teléfonos móviles a fin de evitar causar errores de margen.

Acerca de la selección de tipo de cuerpo atlético

- **Se recomienda que las personas mayores de 18 años y que cumplan los siguientes requisitos seleccionen el "modo atlético" y lo utilicen como valores de referencia.**
  - Personas que realizan al menos 12 horas de ejercicio físico por semana.
  - Personas que pertenecen a un equipo u organización deportiva con el fin de participar en competiciones, etc.
  - Personas que practican físicoculturismo.
  - Atletas profesionales.

Atención

- **Postura durante la medición**
  - Párese con ambos pies paralelos sobre los electrodos.
  - Párese derecho sin doblar las rodillas.
- **El margen de introducción de edad es de 5 a 99 años.**
  En el caso de personas mayores de 100 años, introduzca 99.

Nota

- La impedancia, que es la base para el cálculo de la composición corporal, varía considerablemente inmediatamente después de realizar ejercicios intensos, por lo que puede no ser medida de forma correcta.
- Si se selecciona "atlético" para tipo corporal, la selección cambia automáticamente a "estándar" en el caso de personas de 17 años o menores.
- Cuando se introduce el peso de la ropa, éste se resta del peso obtenido durante la medición.

Cuando se selecciona flujo estándar (no el modo de un solo paso)

En el modo de flujo estándar, primero se mide el peso, luego se introducen los datos personales y, finalmente, se mide la composición corporal.

Medición del peso ▶ **Introducción de datos personales** ▶ **Medición de la composición corporal**

1 Pulse (ON/OFF) para encender la unidad.

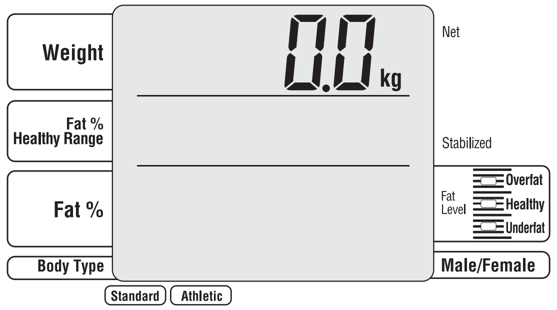

(kg modo)

2 Compruebe que el monitor de composición corporal está seleccionado e introduzca el peso de la ropa.

Introduzca la información pulsando (0) – (9) y (.).

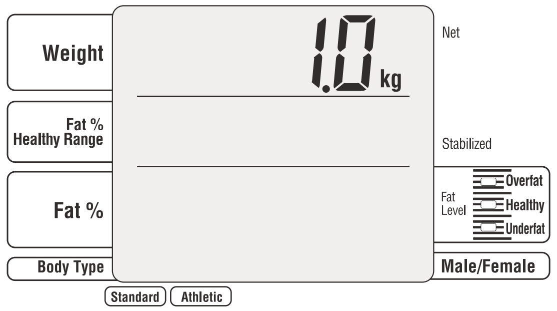

Nota

- El peso de la ropa (tara preestablecida) puede introducirse dentro del margen de 0,0-20,0lb o 0,0-10,0kg.
  *incrementos de 0,2lb / 0,1kg
  *Unidad se cambia por (kg / lb) clave.
- Para corregir el valor introducido:
  ⇨pulse (CE) (se borra la información introducida).

3 Pulse (Enter / Next).

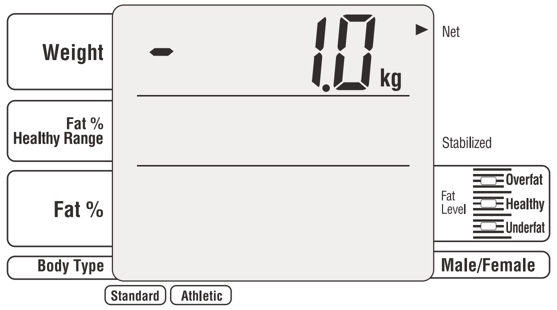

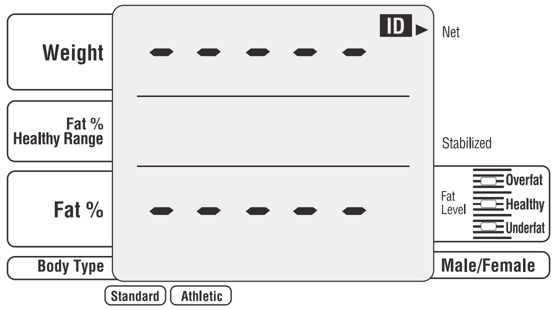

Nota

- **Esta pantalla no aparece si se ha seleccionado OFF en "con o sin ID" (☞ página 141). (Aparece la pantalla "selección del tipo corporal")**

4 Introduzca el número de ID.

Introdúzcalo pulsando (0) – (9).

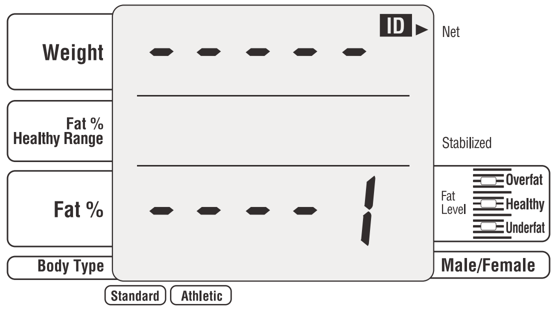

Nota

- **Esta pantalla no aparece si se ha seleccionado OFF en "con o sin ID" (☞ página 141).**
- El número de ID puede introducirse dentro del margen de 0 a 9999999999. Si se pulsa (Enter / Next), los dígitos no introducidos se rellenan con ceros.
- Si ha introducido información incorrecta:
  ⇨pulse (CE) (se borra la información introducida).
- Si se pulsa (CE) sin haber introducido un número de ID, la máquina vuelve a mostrar la pantalla "inicio de medición".

5 Seleccione el tipo corporal.

Pulse las teclas de selección de tipo corporal para introducir la información.
Cuando se selecciona el tipo corporal, la lámpara parpadea en "Gender" (sexo).

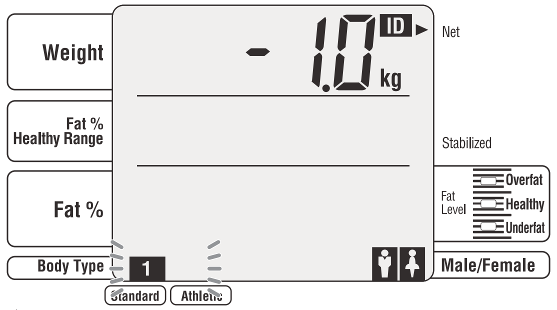

Nota

- **Esta pantalla no aparece si se ha seleccionado OFF en "ON u OFF para el modo atlético" (☞ página 142).**
- El tipo corporal también puede seleccionarse con las teclas numéricas (1 2).
- Si ha introducido información incorrecta:
  ⇨pulse CE (se borra la información introducida y la máquina vuelve a mostrar la pantalla "selección de tipo corporal").
- Si se pulsa CE (borrar) sin haber introducido el tipo corporal, la máquina vuelve a mostrar la pantalla "introducción de número de ID" (o la pantalla "inicio de medición").

6 Seleccione el sexo.

Pulse las teclas de selección de sexo masculino / femenino para introducir la información.
Cuando se selecciona masculino o femenino, la lámpara parpadea en "age input" (introducción de edad).

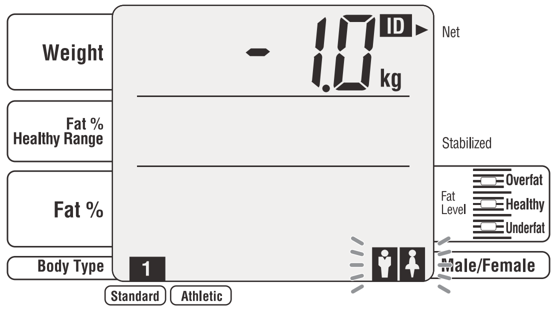

Nota

- Si ha introducido información incorrecta:
  ⇨pulse CE (se borra la información introducida y la máquina vuelve a mostrar la pantalla "selección de sexo").
- Si se pulsa CE sin haber seleccionado sexo masculino o femenino, la máquina vuelve a mostrar la pantalla "selección de tipo corporal".

7 Introduzca la edad.

Introdúzcala pulsando 0 – 9.

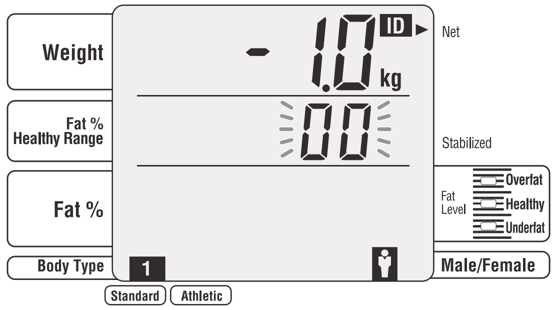

Nota

- La edad puede introducirse dentro del margen de 5 a 99.
- Si ha introducido información incorrecta:
  ⇨pulse CE (se borra la información introducida).
- Si se pulsa CE sin haber introducido la edad, la máquina vuelve a mostrar la pantalla "selección de sexo masculino/femenino".

8 Pulse Enter / Next.

Cuando se introduce la edad, la lámpara parpadea en "height input" (introducción de estatura).

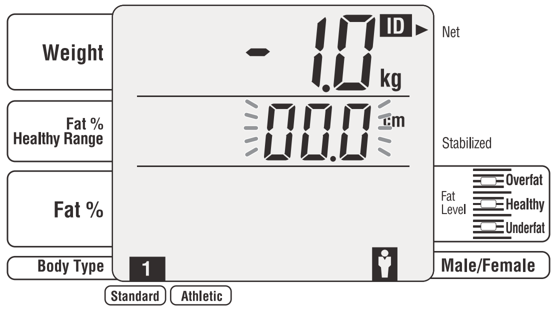

9 Introduzca la estatura.

Introdúzcala pulsando 0 – 9.

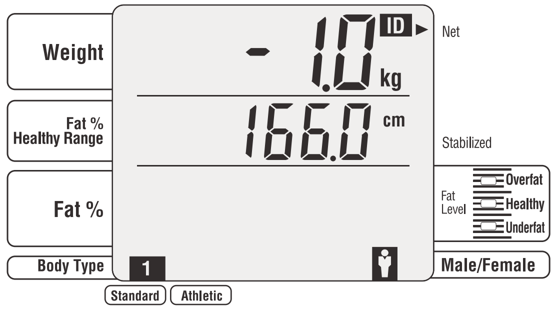

Nota

- La estatura puede introducirse dentro del margen de 3ft -7ft11,5in /90,0 a 249,9 (90 a 249).
  *Unidad se cambia por kg / lb clave.
- Si ha introducido información incorrecta:
  ⇨pulse CE (se borra la información introducida).
- Si se pulsa CE sin haber introducido la estatura, la máquina vuelve a mostrar la pantalla "selección de edad".

10 Pulse Enter / Next.

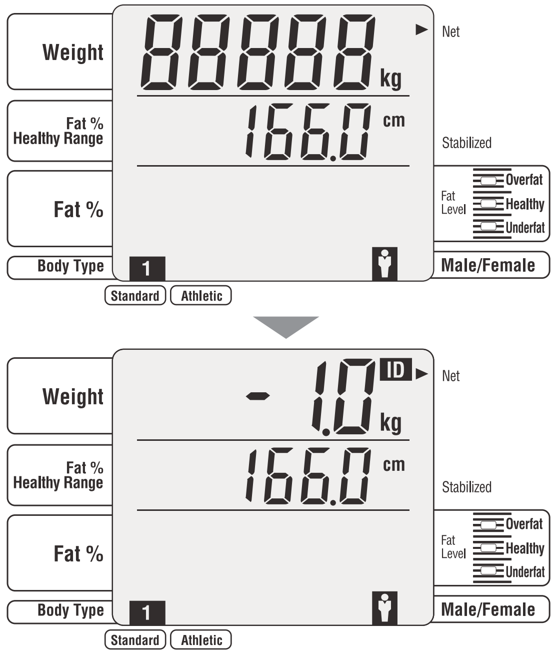

La lámpara "step on" (suba a la plataforma) parpadea.

Nota

- Cuando se pulsa CE, la máquina vuelve a mostrar la pantalla anterior..

11 Step on the electrodes with bare feet.

Take off your socks and stockings before stepping on.

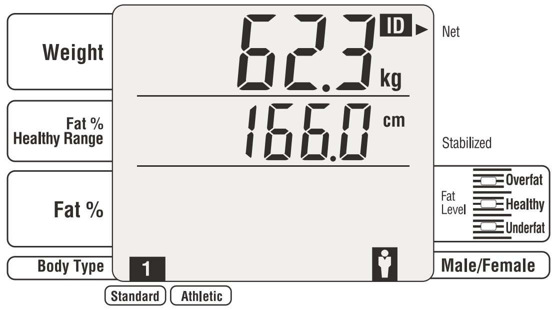

12 Se realiza la medición de la composición corporal.

La indicación 88888 se apaga de forma secuencial.

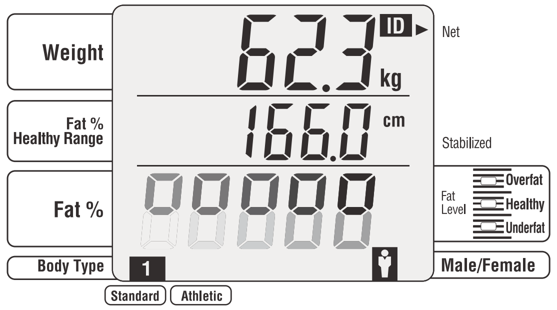

13 La medición finaliza.

Se visualiza el resultado de la medición y la estimación del porcentaje de grasa corporal.
La información se imprime automáticamente. (Si se ha introducido un número distinto de 0 en "Selección del número de hojas a imprimir" (☞ página 139).

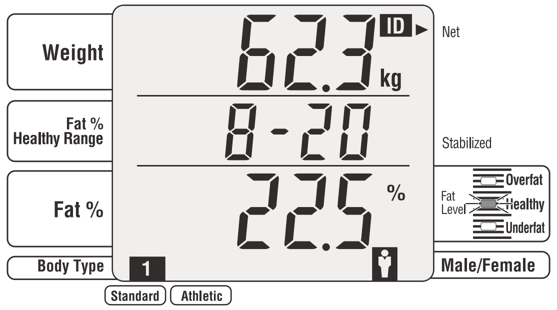

Nota

- **Margen de grasa saludable (el valor numérico en la etapa intermedia) no se visualiza si se ha seleccionado OFF en "ON / OFF para Visualización del margen de % de grasa saludable" (☞ página 141).**

Baje de la plataforma.
La máquina vuelve a mostrar la pantalla "inicio de medición".

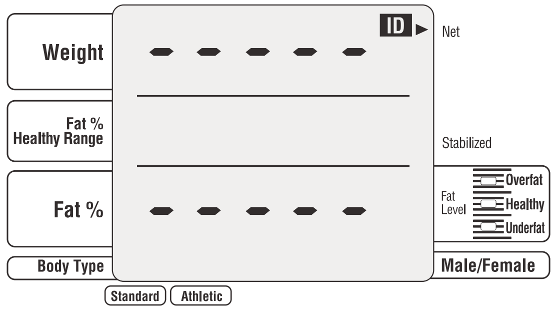

Cómo leer la información de la pantalla <kg modo>

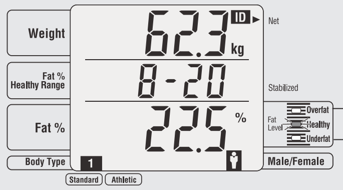

Evaluación basada en el porcentaje de grasa corporal. (☞ página 166.)

Cómo leer la información de la pantalla <lb modo>

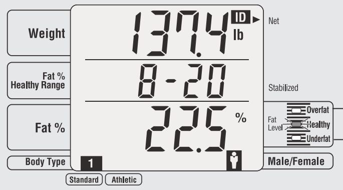

Evaluación basada en el porcentaje de grasa corporal. (☞ página 166.)

Alternativa (no el modo de un solo paso)

En el modo de un solo paso, después de introducir los datos personales, se mide el peso y la composición corporal.

Introducción de datos personales ▶ **Medición del peso** ▶ **Medición de la composición corporal**

1 Pulse ON/OFF para encender la unidad.

Nota

- Unidad se cambia por kg / lb clave.

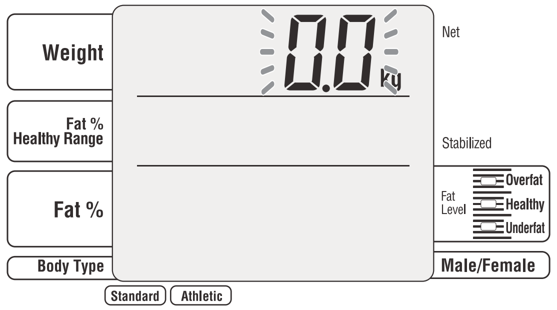

(kg modo)

2 Compruebe que el monitor de composición corporal está seleccionado e introduzca el peso de la ropa.

Introduzca la información pulsando 0 – 9 y . .

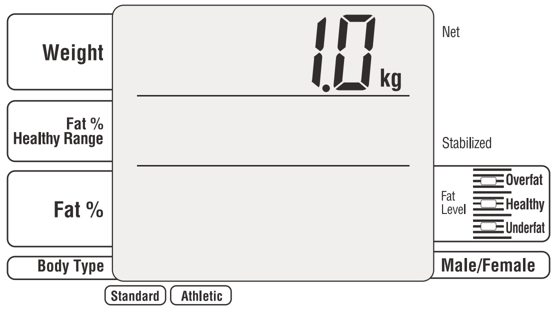

Nota

- El peso de la ropa (tara preestablecida) puede introducirse dentro del margen de 0,0-20,0lb o 0,0-10,0kg.
  *incrementos de 0,2lb / 0,1kg
  *Unidad se cambia por kg / lb clave.
- Para corregir el valor introducido:
  ⇨pulse CE (se borra la información introducida).

3 Pulse Enter / Next.

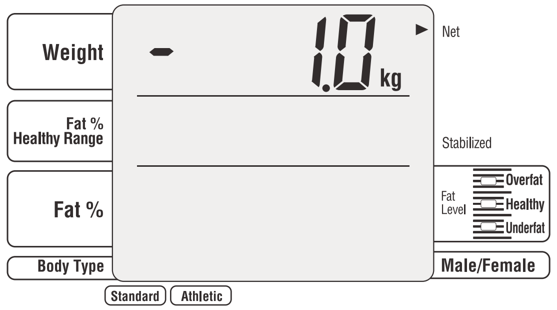

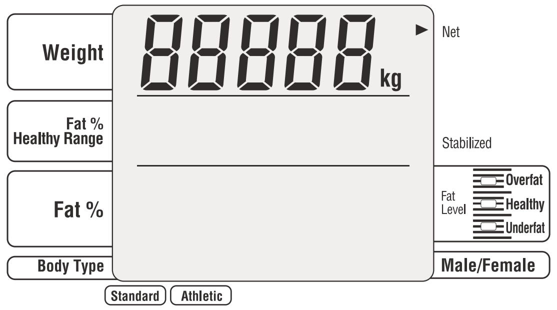

La lámpara "step on" (suba a la plataforma) parpadea.

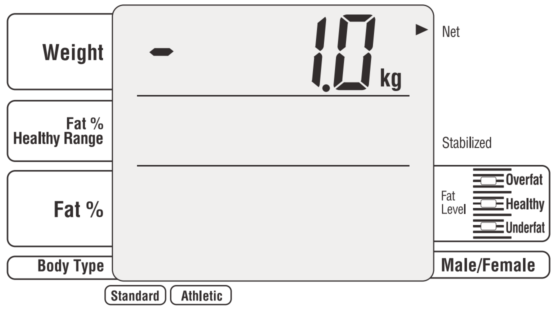

Nota

- Cuando se pulsa CE, la máquina vuelve a mostrar la pantalla anterior.

4 Pise los electrodos con los pies descalzos.

Quítese los calcetines o medias antes de pisar la báscula.

<Niño> <Adulto>

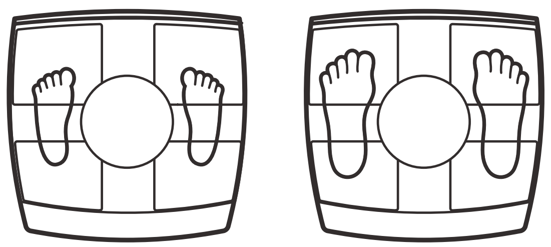

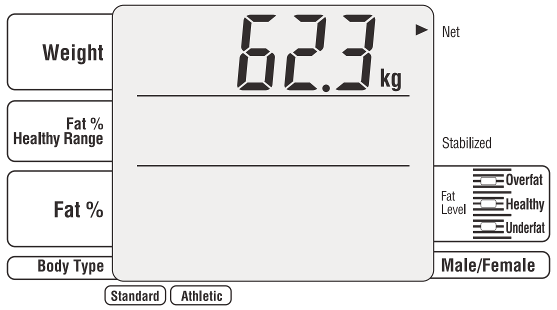

Cuando el peso se estabiliza, la máquina cambia a la pantalla que se muestra a la derecha.

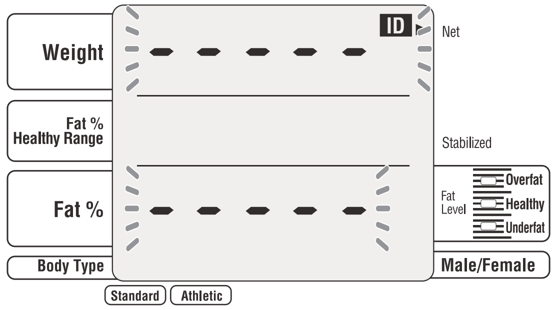

Nota

- No se baje de la plataforma.
- **Esta pantalla no aparece si se ha seleccionado OFF en "con o sin ID" (☞ página 141). (Aparece la pantalla "selección del tipo corporal")**

5 Introduzca el número de ID.

Introdúzcalo pulsando (0) – (9).

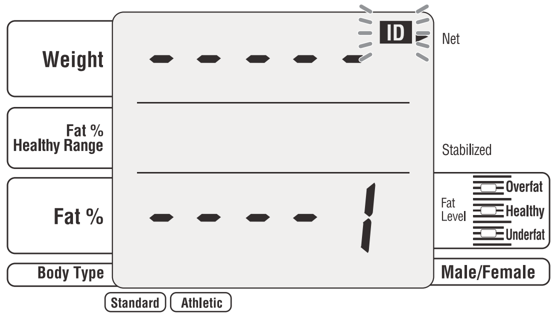

Nota

- **Esta pantalla no aparece si se ha seleccionado OFF en "con o sin ID" (☞ página 141).**
- El número de ID puede introducirse dentro del margen de 0 a 9999999999. Si se pulsa (Enter / Next), los dígitos no introducidos se rellenan con ceros.
- Si ha introducido información incorrecta:
  ⇨pulse (CE) (se borra la información introducida).
- Si se pulsa (CE) sin haber introducido un número de ID, la máquina vuelve a mostrar la pantalla "inicio de medición".

6 Pulse (Enter / Next).

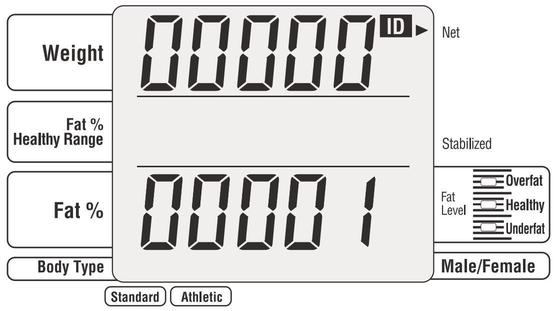

Nota

- **Esta pantalla no aparece si se ha seleccionado OFF en "con o sin ID" (☞ página 141).**

7 Seleccione el tipo corporal.

Pulse las teclas de selección de tipo corporal para introducir la información.
Cuando se selecciona el tipo corporal, la lámpara parpadea en "Gender" (sexo).

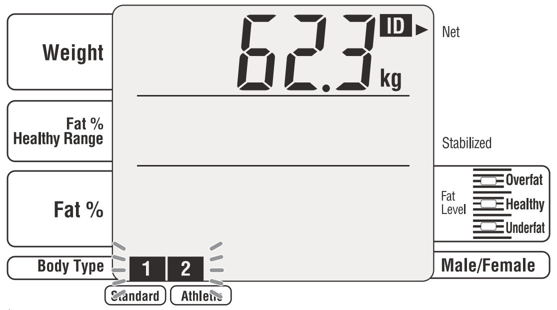

Nota

- **Esta pantalla no aparece si se ha seleccionado OFF en "ON u OFF para el modo atlético"(☞ página 142).**
- El tipo corporal también puede seleccionarse con las teclas numéricas ((1)(2)).
- Si ha introducido información incorrecta:
  ⇨pulse (CE) (se borra la información introducida y la máquina vuelve a mostrar la pantalla "selección de tipo corporal").
- Si se pulsa (CE) (borrar) sin haber introducido el tipo corporal, la máquina vuelve a mostrar la pantalla "introducción de número de ID" (o la pantalla "inicio de medición").

8 Seleccione el sexo.

Pulse las teclas de selección de sexo masculino / femenino para introducir la información.
Cuando se selecciona masculino o femenino, la lámpara parpadea en "age input" (introducción de edad).

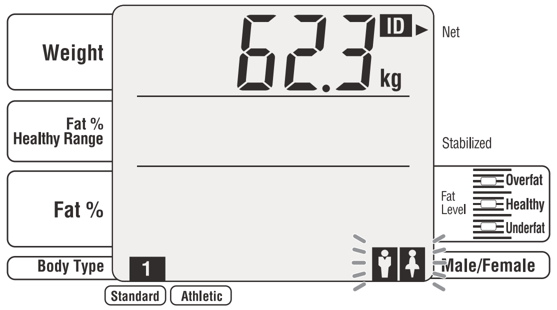

Nota

- Si ha introducido información incorrecta:
  ⇨pulse (CE) (se borra la información introducida y la máquina vuelve a mostrar la pantalla "selección de sexo").
- Si se pulsa (CE) sin haber seleccionado sexo masculino o femenino, la máquina vuelve a mostrar la pantalla "selección de tipo corporal".

9 Introduzca la edad.

Introdúzcala pulsando (0) – (9).

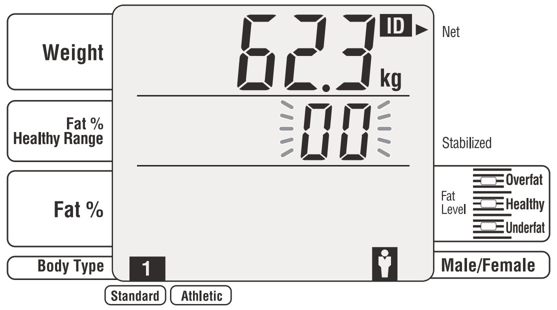

Nota

- La edad puede introducirse dentro del margen de 5 a 99.
- Si ha introducido información incorrecta:
  ⇨pulse (CE) (se borra la información introducida).
- Si se pulsa (CE) sin haber introducido la edad, la máquina vuelve a mostrar la pantalla "selección de sexo masculino/femenino".

10 Pulse Enter / Next.

Cuando se introduce la edad, la lámpara parpadea en “height input” (introducción de estatura).

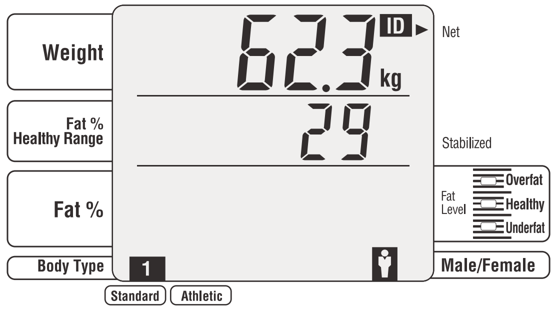

11 Introduzca la estatura.

Introdúzcala pulsando 0 – 9.

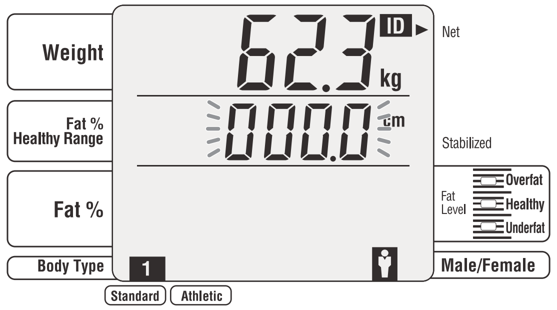

Nota

- La estatura puede introducirse dentro del margen de 3ft -7ft11,5in / 90,0-249,9cm.
  *Unidad se cambia por kg / lb clave.
- Si ha introducido información incorrecta:
  ⇨pulse CE (se borra la información introducida).
- Si se pulsa CE sin haber introducido la estatura, la máquina vuelve a mostrar la pantalla “selección de edad”.

12 Pulse Enter / Next.

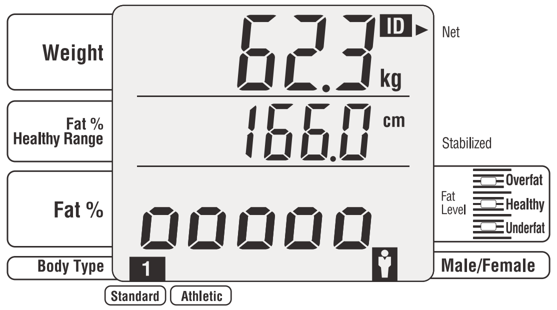

13 Se realiza la medición de la composición corporal.

La indicación 88888 se apaga de forma secuencial.

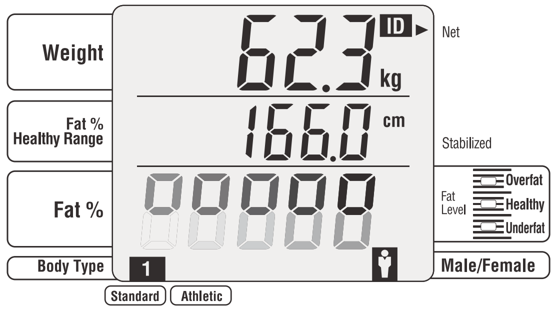

14 La medición finaliza.

Se visualiza el resultado de la medición y la estimación del porcentaje de grasa corporal.
La información se imprime automáticamente. (Si se ha introducido un número distinto de 0 en “Selección del número de hojas a imprimir” (☞ página 139).

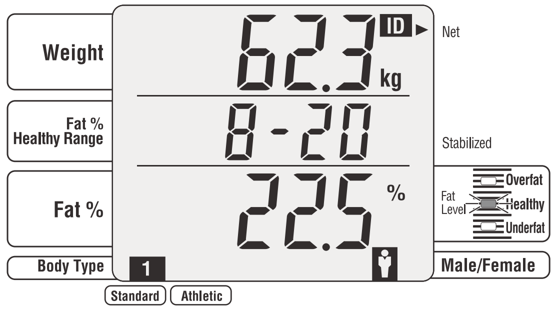

Nota

- **Margen de grasa saludable (el valor numérico en la etapa intermedia) no se visualiza si se ha seleccionado OFF en “ON / OFF para Visualización del margen de % de grasa saludable” (☞ página 141).**

Baje de la plataforma.
La máquina vuelve a mostrar la pantalla “inicio de medición”.

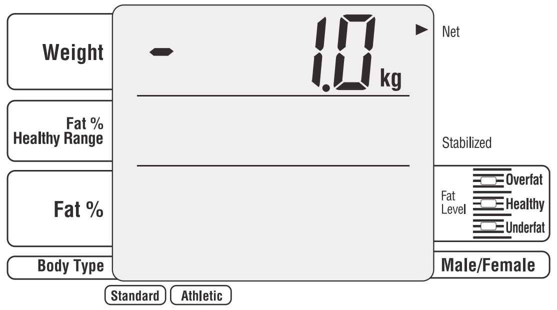

Cómo leer la información de la pantalla <kg modo>

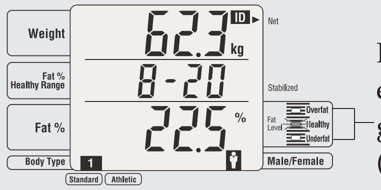

Evaluación basada en el porcentaje de grasa corporal. (☞ página 166.)

Cómo leer la información de la pantalla <lb modo>

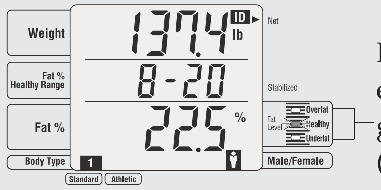

Evaluación basada en el porcentaje de grasa corporal. (☞ página 166.)

Instrucciones de uso (Índice de grasa corporal deseado)

1 Introduzca el índice de grasa corporal deseado.

Introdúzcalo pulsando un número del 0 al 9.

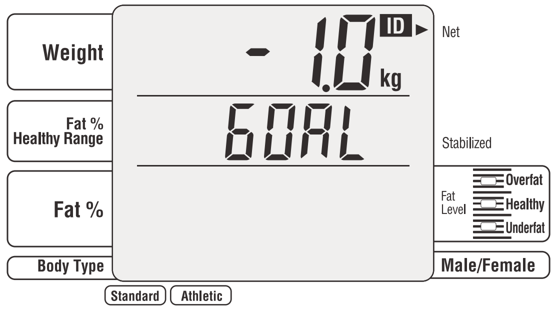

Nota

- Se visualizará esta pantalla si la configuración ON/OFF del índice de grasa corporal deseado se encuentra en posición ON (☞ página 145).
- Puede introducir un índice de grasa corporal deseado de 4 a 55.
- Si introduce una cifra incorrecta,
  ⇨Pulse CE (la cifra se borra).

Si el número de la página a imprimir se fija en "0", la función de configuración del índice de grasa corporal deseado se apagará (OFF) automáticamente.
Si el índice de grasa corporal deseado se fija en 0 ó nada, este no se imprimirá.

⚠PRECAUCIONES Antes de iniciar un programa de gestión del peso corporal y configurar el índice de grasa corporal más adecuado, consulte a su médico. Tanita no se responsabiliza de la configuración del índice de grasa corporal más adecuado para personas específicas.

Cómo realizar una medición (al usar la máquina como báscula)

1 Pulse ON/OFF para encender la unidad.

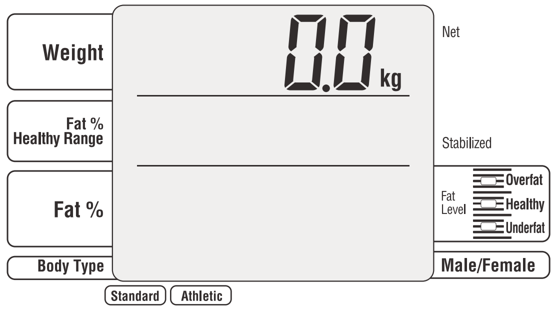

2 Compruebe que el monitor de composición corporal está seleccionado e introduzca el peso de la ropa.

Introdúzcalo pulsando 0 – 9 al ·.

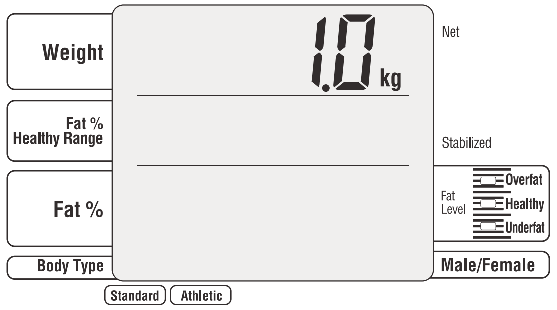

Nota

- El peso de la ropa (tara preestablecida) puede introducirse dentro del margen de 0,0-20,0lb o 0,0-10,0kg.
  *incrementos de 0,2lb / 0,1kg
  *Unidad se cambia por kg / lb clave.
- Si ha introducido información incorrecta:
  ⇨pulse CE (Se borra la información introducida).

3 Pulse Enter / Next.

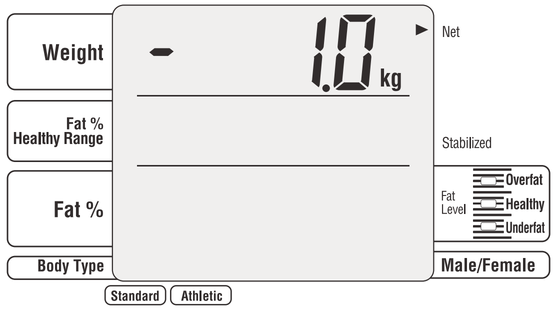

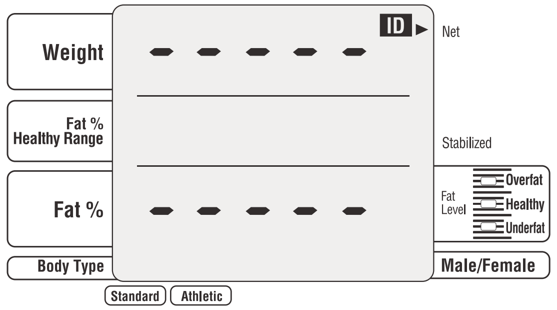

Nota

- **Esta pantalla no se muestra si la "configuración con o sin Identificador de usuario" de la página 139 está seleccionada en OFF (☞ página 141). (Se muestra la pantalla de "selección de tipo de estructura corporal.)**

4 Introduzca un número de ID.

Introdúzcalo pulsando 0 – 9.

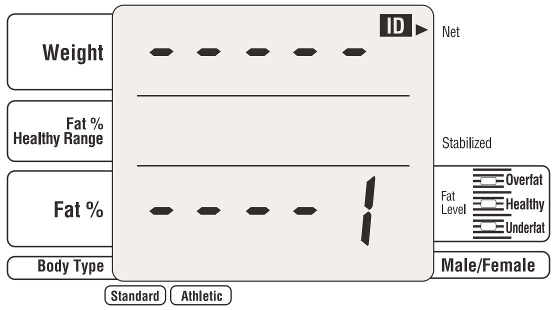

Nota

- **Esta pantalla no aparece si se ha seleccionado OFF en "con o sin ID" (☞ página 141).**
- El número de ID puede introducirse dentro del margen de 0 a 9999999999.
- Si ha introducido información incorrecta:
  ⇨pulse CE (se borra la información introducida).
- Si se pulsa CE sin haber introducido un número de ID, la máquina vuelve a mostrar la pantalla "inicio de medición".

5 Pulse Enter / Next.

Nota

- **Esta pantalla no aparece si se ha seleccionado OFF en "con o sin ID" (☞ página 141).**

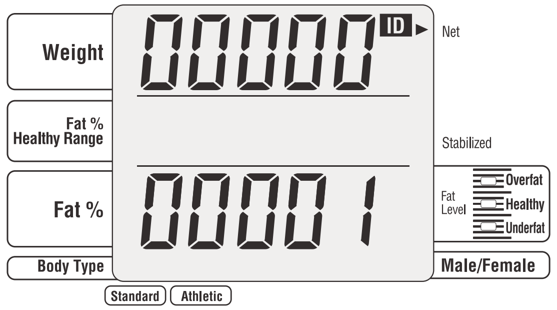

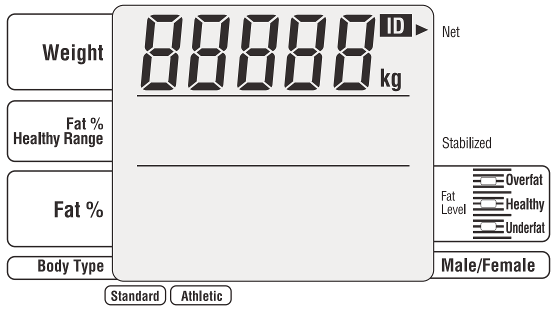

La lámpara parpadea para "step on" (suba a la plataforma).

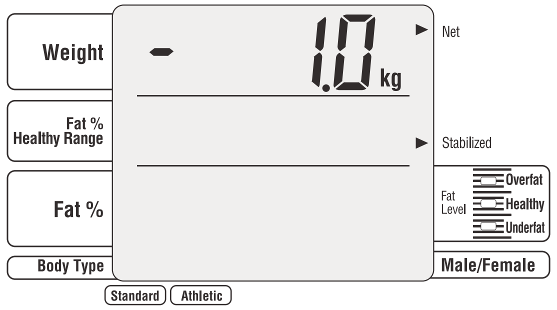

Nota

- Si se pulsa CE, la máquina vuelve a mostrar la pantalla anterior.

6 La medición finaliza.

La información se imprime automáticamente (si se ha introducido un número distinto de 0 en "Selección del número de hojas a imprimir" (☞ página 139).

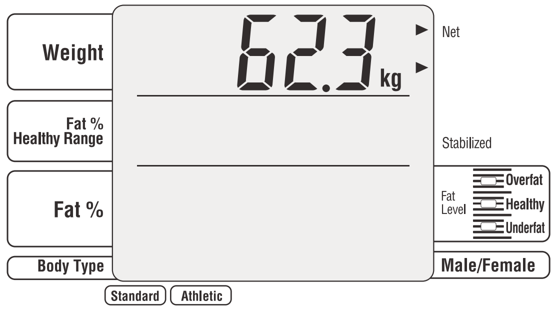

Cuando se baje de la plataforma, la máquina cambiará a la pantalla de inicio de medición.

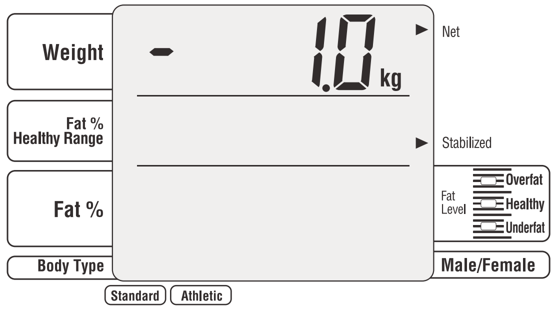

- ¿Que es el porcentaje de grasa corporal?

Porcentaje de grasa corporal es la cantidad de grasa corporal expresada como una proporción del peso corporal.
Se ha demostrado que la reducción de los niveles excesivos de grasa corporal disminuye el riesgo de ciertas enfermedades tales como la hipertensión arterial, las enfermedades cardiacas, la diabetes y el cáncer. En el gráfico se muestran los niveles saludables de grasa corporal.

Ìndices de grasa corporal para niños medianos [1,2]
Margen de grasa corporal para adultos normales [3,4]

[1] *Susan Jebb et al. Obesity Research 2004;12;A156-157 "Nuevas curvas de referencia de grasa corporal para niños"*

[2] *Gallagher D et al. Am J Clin Nutr 2000,72:694-701. "Healthy percentage body fat ranges:an approach for developing guidelines based on body mass index."*

Bajo en grasa — Saludable — Alto en grasa — Obeso

Mujer Edad	1	2	3	4	5	6	7	8	9	10	11	12	13	14	15	16	17	18	19	20	21	22	23	24	25	26	27	28	29	30	31	32	33	34	35	36	37	38	39	40	41	42	43	44	45
5	1	2	3	4	5	6	7	8	9	10	11	12	13	14	15	16	17	18	19	20	21	22	23	24	25	26	27	28	29	30	31	32	33	34	35	36	37	38	39	40	41	42	43	44	45
6	1	2	3	4	5	6	7	8	9	10	11	12	13	14	15	16	17	18	19	20	21	22	23	24	25	26	27	28	29	30	31	32	33	34	35	36	37	38	39	40	41	42	43	44	45
7	1	2	3	4	5	6	7	8	9	10	11	12	13	14	15	16	17	18	19	20	21	22	23	24	25	26	27	28	29	30	31	32	33	34	35	36	37	38	39	40	41	42	43	44	45
8	1	2	3	4	5	6	7	8	9	10	11	12	13	14	15	16	17	18	19	20	21	22	23	24	25	26	27	28	29	30	31	32	33	34	35	36	37	38	39	40	41	42	43	44	45
9	1	2	3	4	5	6	7	8	9	10	11	12	13	14	15	16	17	18	19	20	21	22	23	24	25	26	27	28	29	30	31	32	33	34	35	36	37	38	39	40	41	42	43	44	45
10	1	2	3	4	5	6	7	8	9	10	11	12	13	14	15	16	17	18	19	20	21	22	23	24	25	26	27	28	29	30	31	32	33	34	35	36	37	38	39	40	41	42	43	44	45
11	1	2	3	4	5	6	7	8	9	10	11	12	13	14	15	16	17	18	19	20	21	22	23	24	25	26	27	28	29	30	31	32	33	34	35	36	37	38	39	40	41	42	43	44	45
12	1	2	3	4	5	6	7	8	9	10	11	12	13	14	15	16	17	18	19	20	21	22	23	24	25	26	27	28	29	30	31	32	33	34	35	36	37	38	39	40	41	42	43	44	45
13	1	2	3	4	5	6	7	8	9	10	11	12	13	14	15	16	17	18	19	20	21	22	23	24	25	26	27	28	29	30	31	32	33	34	35	36	37	38	39	40	41	42	43	44	45
14	1	2	3	4	5	6	7	8	9	10	11	12	13	14	15	16	17	18	19	20	21	22	23	24	25	26	27	28	29	30	31	32	33	34	35	36	37	38	39	40	41	42	43	44	45
15	1	2	3	4	5	6	7	8	9	10	11	12	13	14	15	16	17	18	19	20	21	22	23	24	25	26	27	28	29	30	31	32	33	34	35	36	37	38	39	40	41	42	43	44	45
16	1	2	3	4	5	6	7	8	9	10	11	12	13	14	15	16	17	18	19	20	21	22	23	24	25	26	27	28	29	30	31	32	33	34	35	36	37	38	39	40	41	42	43	44	45
17	1	2	3	4	5	6	7	8	9	10	11	12	13	14	15	16	17	18	19	20	21	22	23	24	25	26	27	28	29	30	31	32	33	34	35	36	37	38	39	40	41	42	43	44	45
18	1	2	3	4	5	6	7	8	9	10	11	12	13	14	15	16	17	18	19	20	21	22	23	24	25	26	27	28	29	30	31	32	33	34	35	36	37	38	39	40	41	42	43	44	45
19	1	2	3	4	5	6	7	8	9	10	11	12	13	14	15	16	17	18	19	20	21	22	23	24	25	26	27	28	29	30	31	32	33	34	35	36	37	38	39	40	41	42	43	44	45
20-39	1	2	3	4	5	6	7	8	9	10	11	12	13	14	15	16	17	18	19	20	21	22	23	24	25	26	27	28	29	30	31	32	33	34	35	36	37	38	39	40	41	42	43	44	45
40-59	1	2	3	4	5	6	7	8	9	10	11	12	13	14	15	16	17	18	19	20	21	22	23	24	25	26	27	28	29	30	31	32	33	34	35	36	37	38	39	40	41	42	43	44	45
60-	1	2	3	4	5	6	7	8	9	10	11	12	13	14	15	16	17	18	19	20	21	22	23	24	25	26	27	28	29	30	31	32	33	34	35	36	37	38	39	40	41	42	43	44	45

0% — 10% — 20% — 30% — 40%

Hombre Edad	1	2	3	4	5	6	7	8	9	10	11	12	13	14	15	16	17	18	19	20	21	22	23	24	25	26	27	28	29	30	31	32	33	34	35	36	37	38	39	40	41	42	43	44	45
5	1	2	3	4	5	6	7	8	9	10	11	12	13	14	15	16	17	18	19	20	21	22	23	24	25	26	27	28	29	30	31	32	33	34	35	36	37	38	39	40	41	42	43	44	45
6	1	2	3	4	5	6	7	8	9	10	11	12	13	14	15	16	17	18	19	20	21	22	23	24	25	26	27	28	29	30	31	32	33	34	35	36	37	38	39	40	41	42	43	44	45
7	1	2	3	4	5	6	7	8	9	10	11	12	13	14	15	16	17	18	19	20	21	22	23	24	25	26	27	28	29	30	31	32	33	34	35	36	37	38	39	40	41	42	43	44	45
8	1	2	3	4	5	6	7	8	9	10	11	12	13	14	15	16	17	18	19	20	21	22	23	24	25	26	27	28	29	30	31	32	33	34	35	36	37	38	39	40	41	42	43	44	45
9	1	2	3	4	5	6	7	8	9	10	11	12	13	14	15	16	17	18	19	20	21	22	23	24	25	26	27	28	29	30	31	32	33	34	35	36	37	38	39	40	41	42	43	44	45
10	1	2	3	4	5	6	7	8	9	10	11	12	13	14	15	16	17	18	19	20	21	22	23	24	25	26	27	28	29	30	31	32	33	34	35	36	37	38	39	40	41	42	43	44	45
11	1	2	3	4	5	6	7	8	9	10	11	12	13	14	15	16	17	18	19	20	21	22	23	24	25	26	27	28	29	30	31	32	33	34	35	36	37	38	39	40	41	42	43	44	45
12	1	2	3	4	5	6	7	8	9	10	11	12	13	14	15	16	17	18	19	20	21	22	23	24	25	26	27	28	29	30	31	32	33	34	35	36	37	38	39	40	41	42	43	44	45
13	1	2	3	4	5	6	7	8	9	10	11	12	13	14	15	16	17	18	19	20	21	22	23	24	25	26	27	28	29	30	31	32	33	34	35	36	37	38	39	40	41	42	43	44	45
14	1	2	3	4	5	6	7	8	9	10	11	12	13	14	15	16	17	18	19	20	21	22	23	24	25	26	27	28	29	30	31	32	33	34	35	36	37	38	39	40	41	42	43	44	45
15	1	2	3	4	5	6	7	8	9	10	11	12	13	14	15	16	17	18	19	20	21	22	23	24	25	26	27	28	29	30	31	32	33	34	35	36	37	38	39	40	41	42	43	44	45
16	1	2	3	4	5	6	7	8	9	10	11	12	13	14	15	16	17	18	19	20	21	22	23	24	25	26	27	28	29	30	31	32	33	34	35	36	37	38	39	40	41	42	43	44	45
17	1	2	3	4	5	6	7	8	9	10	11	12	13	14	15	16	17	18	19	20	21	22	23	24	25	26	27	28	29	30	31	32	33	34	35	36	37	38	39	40	41	42	43	44	45
18	1	2	3	4	5	6	7	8	9	10	11	12	13	14	15	16	17	18	19	20	21	22	23	24	25	26	27	28	29	30	31	32	33	34	35	36	37	38	39	40	41	42	43	44	45
19	1	2	3	4	5	6	7	8	9	10	11	12	13	14	15	16	17	18	19	20	21	22	23	24	25	26	27	28	29	30	31	32	33	34	35	36	37	38	39	40	41	42	43	44	45
20-39	1	2	3	4	5	6	7	8	9	10	11	12	13	14	15	16	17	18	19	20	21	22	23	24	25	26	27	28	29	30	31	32	33	34	35	36	37	38	39	40	41	42	43	44	45
40-59	1	2	3	4	5	6	7	8	9	10	11	12	13	14	15	16	17	18	19	20	21	22	23	24	25	26	27	28	29	30	31	32	33	34	35	36	37	38	39	40	41	42	43	44	45
60-	1	2	3	4	5	6	7	8	9	10	11	12	13	14	15	16	17	18	19	20	21	22	23	24	25	26	27	28	29	30	31	32	33	34	35	36	37	38	39	40	41	42	43	44	45

Bajo en grasa — Saludable — Alto en grasa — Obeso

Copyright (C) 2004 TANITA Corporation. All Rights Reserved.

Indicador de los niveles saludables de grasa corporal

El monitor de composición corporal compara automáticamente su porcentaje de grasa corporal con el gráfico de niveles saludables de grasa corporal. Después de realizado el cálculo de porcentaje de grasa corporal, en la parte inferior del display, parpadeará una barra negra que identifica su posición en los niveles de grasa corporal para su edad y sexo.

– | 0 | + | ++

(-) : Bajo en grasa; por debajo del margen saludable de grasa corporal. Aumento del riesgo de problemas de salud.
(0) : Saludable; dentro del margen saludable de grasa corporal para su edad/sexo.
(+) : Alto en grasa; por encima del margen saludable. Aumento del riesgo de problemas de salud.
(++) : Obeso; muy por encima del margen saludable de grasa corporal. Aumento considerable del riesgo de problemas de salud relacionados con la obesidad.

***Note :** Si selecciona el modo de Atleta, la unidad no mostrará el indicador de margen saludable. Los Atletas podrían tener un margen de grasa corporal más bajo según su deporte o actividad particulares.

- ¿Que es el porcentaje de agua corporal total?

Porcentaje de agua corporal total es la cantidad total de líquido que hay en el cuerpo de una persona, expresada como porcentaje de su peso total.
El agua desempeña un papel vital en muchos de los procesos corporales, y se encuentra en todas las células, tejidos y órganos del cuerpo. El mantenimiento de un porcentaje adecuado de agua corporal total garantizará que el cuerpo funcione de manera eficiente, y reducirá el riesgo de aparición de los trastornos de salud asociados.

Los niveles de agua del cuerpo fluctúan naturalmente a lo largo del día. De hecho, el cuerpo tiende a deshidratarse después de una noche larga y se observan diferencias en la distribución de fluidos entre el día y la noche. Ingerir grandes cantidades de comida, beber alcohol, menstruar, enfermarse, hacer ejercicio y bañarse pueden provocar variaciones en los niveles de hidratación.

Su porcentaje de agua corporal debe servirle de guía, y no debe ser utilizado específicamente para determinar el porcentaje de agua corporal total absoluto recomendado para usted. Es importante tratar de lograr cambios a largo plazo en el porcentaje de agua corporal total y mantenerlo a un nivel saludable y constante.

Si bebe de golpe una gran cantidad de agua, su nivel de agua no cambiará inmediatamente. De hecho, aumentará la lectura de su grasa corporal debido al incremento de peso. Supervise periódicamente todas las lecturas para realizar un seguimiento del cambio relativo.

El porcentaje varía de una persona a otra, pero como guía, los porcentajes de agua corporal total medios para un adulto saludable son:
Mujer **:** 45 a 60%
Varón **:** 50 a 65%
Fuente **:** Información basada en investigación interna de Tanita

Nota: El porcentaje de agua corporal total tenderá a disminuir a medida que el porcentaje de grasa corporal aumente. Una persona que tenga un alto porcentaje de grasa corporal puede estar por debajo del porcentaje de agua corporal medio. A medida que pierda grasa corporal, el porcentaje de agua corporal total deberá acercarse gradualmente a los niveles típicos indicados anteriormente.

- ¿Que es el nivel de grasa visceral?

Esta función indica el nivel de grasa visceral de su cuerpo.
Grasa visceral es la grasa que se encuentra en el interior de la cavidad abdominal, rodeando los órganos vitales de la zona del tronco (zona abdominal). Las investigaciones han demostrado que aunque el peso y la grasa corporal se mantengan constantes, a medida que se envejece varía la distribución de la grasa en el cuerpo, y que es mucho más probable que ésta se desplace a la zona del tronco, especialmente después de la menopausia. Asegurándose de tener los niveles sanos de grasa visceral reducirá el riesgo de contraer ciertas enfermedades tales como enfermedades cardiacas e hipertensión arterial, y retardar la aparición de la diabetes tipo 2.

El monitor de composición corporal Tanita le proporcionará una medición del nivel de grasa corporal en una escala que va del 1 al 59.

1 a 12
Indica que usted tiene un nivel saludable de grasa visceral. Continúe controlando su nivel de grasa visceral para garantizar que siempre mantenga el nivel adecuado.

13 a -59
Indica que usted tiene un nivel excesivo de grasa visceral. Piense en la introducción de algunos cambios en su forma de vida, posiblemente a través de un cambio en su dieta o haciendo más ejercicios.

Fuente : Información de la Universidad de Columbia (Nueva York) y del Instituto Tanita (Tokio)

Nota:
- Aunque usted tenga un índice de grasa corporal bajo, es posible que tenga un nivel de grasa visceral alto.
- Si desea un diagnóstico medico, consulte a un medico.

- ¿Que es el metabolismo basal (MB)?

¿QUÉ ES BMR?
El MB es el nivel mínimo de energía que su cuerpo necesita para funcionar eficientemente en reposo, incluyendo los órganos de los sistemas respiratorio y circulatorio, el sistema nervioso, el hígado, los riñones y otros órganos. Usted quema calorías independientemente de la actividad que esté realizando, incluso cuando duerme.

Aproximadamente un 70 % de las calorías que se consumen diariamente son utilizadas para el metabolismo basal. Además, cuando se realiza cualquier tipo de actividad se utiliza energía, sin embargo, cuanto más vigorosa sea esa actividad, mayor será la cantidad de calorías que se queme. Ello se debe a que el músculo estriado (que representa aproximadamente un 40% del peso corporal) funciona como un motor y utiliza una gran cantidad de energía. El metabolismo basal se ve afectado en gran medida por la cantidad de músculos que usted tenga; el aumento de la masa muscular ayuda al metabolismo basal.

Realizando estudios con individuos sanos, los científicos han observado que a medida que las personas envejecen, varía su metabolismo basal. El metabolismo basal aumenta a medida que el niño crece. Después de alcanzar un máximo, a la edad de 16 ó 17 años, el metabolismo basal comienza a disminuir como se muestra en el gráfico que aparece a continuación.

Tener un metabolismo basal más elevado aumentará la cantidad de calorías utilizadas y ayudará a reducir la cantidad de grasa corporal. Un metabolismo basal bajo hará que resulte más difícil perder grasa corporal y peso en general.

¿CÓMO CALCULA EL MONITOR DE COMPOSICIÓN CORPORAL TANITA EL MB?
El método básico para calcular el MB es una ecuación estándar que utiliza el peso y la edad. Tanita ha investigado exhaustivamente la relación entre el MB y la composición corporal, por lo que ofrece al usuario una medición mucho más exacta y personalizada, basada en la medición de la impedancia. Este método ha sido validado médicamente mediante la utilización de calorimetría indirecta (análisis de la composición del aliento)*.

* Reliability on equation for Basal Metabolic Rate: At: 2002 Nutrition Week: A Scientific and Clinical Forum and Exposition Title: International Comparison: Resting Energy Expenditure Prediction Models: The American Journal of CLINICAL NUTRITION (1Fiabilidad de la ecuación para el cálculo del metabolismo basal: Publicado en: Semana de nutrición del año 2002: Foro científico y clínico, y título de exposición: Comparación internacional: Modelos para la predicción del gasto de energía en estado de reposo: Revista Americana de NUTRICIÓN CLÍNICA).

- ¿Qué es la edad metabólica?

Esta función calcula el MB e indica la edad media asociada a ese tipo de metabolismo.
Si su edad metabólica es mayor que su edad real, ello es una señal de que necesita mejorar su metabolismo basal. Hacer más ejercicios ayudará a la creación de tejido muscular sano, lo que a su vez mejorará su edad metabólica.

Los valores de las mediciones que se visualizan en el display van de 12 a 50. Si el valor de la medición fuera menor de 12 aparecerá en el display como “12”, y si fuera mayor de 50 aparecerá como “50”.

- ¿Que es la masa muscular?

El valor de masa muscular que aparece en el display incluye los músculos esqueléticos, los músculos lisos (tales como los del corazón y del aparato digestivo) así como el agua contenida en los mismos.
Los músculos desempeñan un papel importante ya que funcionan como un motor en cuanto a lo que a consumo de energía se refiere.
A medida que aumenta su masa muscular, aumenta el consumo su de energía, lo que le ayuda a reducir los niveles excesivos de grasa corporal y a perder peso de una manera saludable.

¿Qué es la complexión física?
Esta función evalúa su complexión física de acuerdo con el nivel de grasa corporal y masa muscular de su cuerpo.
A medida que aumenta su actividad y reduce la cantidad de grasa corporal, también variará su complexión física, en correspondencia.
Aunque es posible que no varíe su peso, su masa muscular y sus niveles de grasa corporal pueden estar variando, mejorando su salud y reduciendo los riesgos de contraer ciertas enfermedades.
Cada persona debe trazarse su propio objetivo de complexión física que desee tener, y seguir una dieta y un programa de fitness para alcanzar ese objetivo.

Resultado	Complexión física	Explicación
1	Preobesa	**Obeso con contextura pequeña**<br>A juzgar por la apariencia para este tipo 1, no parecen gordos. Sin embargo, tienen un índice alto de grasa corporal con masa muscular baja.
2	Obesa	**Obeso con contextura mediana**<br>En tanto este tipo 2 tiene un porcentaje alto de grasa corporal, el nivel de masa muscular es moderado.
3	Complexión robusta	**Obeso con contextura grande**<br>Este tipo tiene altos tanto el porcentaje de grasa corporal como el de masa muscular, comparados con los de una persona promedio.
4	Falta de ejercicios	**Masa muscular baja y porcentaje de grasa corporal promedio**<br>Este grupo tiene un índice de grasa corporal promedio en tanto la masa muscular es baja comparada con la promedio.
5	Estándar	**Masa muscular alta y porcentaje de grasa corporal promedio**<br>Aquéllos que tienen tanto el índice de grasa corporal como la masa muscular apropiados.
6	Musculosa estándar	**Masa muscular promedio y porcentaje de grasa corporal promedio (Atleta)**<br>Aquéllos que tienen una masa muscular más alta en tanto su porcentaje de grasa corporal es promedio.
7	Delgada	**Masa muscular baja y grasa corporal baja**<br>Este tipo tiene un índice de grasa corporal y de masa muscular más bajos.
8	Delgada y musculosa	**Delgado y musculoso (Atleta)**<br>Este tipo tiene un índice de grasa corporal bajo en tanto tiene una adecuada masa muscular.
9	Muy musculosa	**Muy musculoso (Atleta)**<br>Este grupo tiene un índice de grasa corporal bajo en tanto tiene mucha masa muscular comparada con la de la persona promedio.

Source : Données de l Université Columbia (New York) & du Tanita Institut (Tokyo)

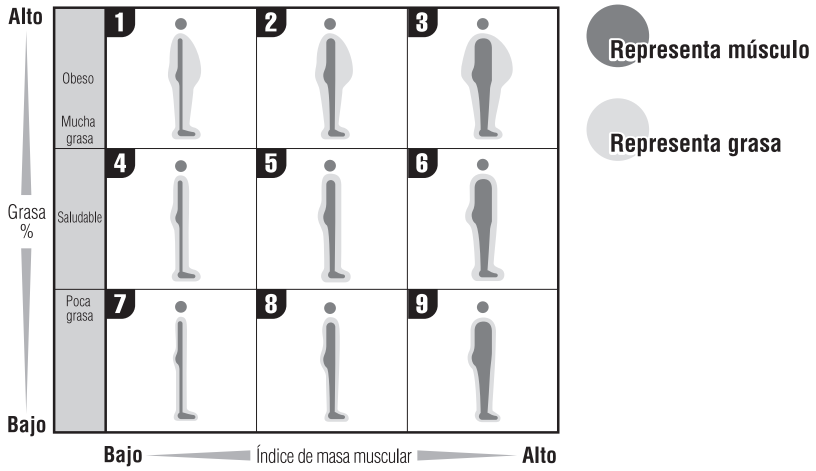

Índice de cantidad de músculo en relación con la altura = Masa muscular (kg) / altura $(cm)^2$

E
Instrucciones de uso
(Diversos criterios)

- ¿Qué se entiende por masa ósea?

Esta función indica la cantidad de hueso (nivel mineral del hueso, calcio y otros minerales) en elcuerpo.

Los estudios realizados han demostrado que el ejercicio y el desarrollo de tejido muscular están relacionados con unos huesos más sanos y fuertes. Mientras que es muy probable que la estructura ósea no experimente cambios notables a corto plazo, es importante que tenga huesos sanos y los cuide a través de una dieta equilibrada y mucho ejercicio. Las personas que estén preocupadas por las enfermedades óseas deberían consultar a su médico de cabecera. Puede que la lectura de la masa ósea de personas que tengan osteoporosis o densidad baja del hueso, debido a una edad avanzada, o juvenil, embarazo, tratamientos hormonales u otras causas no sea del todo precisa.

A continuación, se incluye el resultado de la masa ósea estimada de las personas de 20 a 40 años _franja de edad en la que se supone que tenemos la mayor cantidad de masa ósea_ por peso. (Fuente: Tanita Body Weight Science Institute).

Utilice los gráficos siguientes como referencia para cotejar la lectura de su masa ósea.

Mujeres : media de la masa ósea estimada

Peso (lb)		
Less than 110 lb	110lb - 165 lb	165 lb and up
4,3 lb	5,3 lb	6,5 lb

Peso (kg)		
Less than 50 kg	50 kg - 75 kg	75 kg and up
1,95 kg	2,40 kg	2,95 kg

Hombres : media de la masa ósea estimada

Peso (lb)		
Less than 143 lb	143 lb - 209 lb	209 lb and up
5,9 lb	7,3 lb	8,1 lb

Peso (kg)		
Less than 65 kg	65 kg - 95 kg	95 kg and up
2,66 kg	3,29 kg	3,69 kg

Nota: Las personas descritas seguidamente pueden obtener lecturas distintas y deben considerar los valores facilitados únicamente como referencia.

- Personas ancianas - - Mujeres durante o después de la menopausia
- Personas que reciban terapia a base de hormonas

La "masa ósea estimada" es el valor calculado estadísticamente en función de su correlación con la cantidad libre de grasa (tejidos distintos al graso). Dicha masa no indica directamente la dureza o resistencia de los huesos, ni el riesgo a sufrir fracturas óseas. Si le preocupa el estado de sus huesos, le recomendamos encarecidamente que consulte a un especialista.

E
Instrucciones de uso (Diversos criterios)

Daño a la unidad

Compruebe los siguientes puntos antes de solicitar reparaciones.

Síntoma		Comprobar
**Cómo medir**	**Error de medición de impedancia**<br>Err40<br>Err67	• Efectúe la medición con los pies descalzos.<br>• Si tiene las plantas de los pies secas, aplique aproximadamente 0,5 ml de agua con el cuentagotas suministrado en los electrodos antes de realizar la medición.<br>• Compruebe la información introducida.
	**Error de punto cero**<br>uuuuu	• Apague la unidad, retire los elementos de la plataforma y vuelva a encender la unidad; luego, vuelva a realizar la medición.
	**El peso medido no es estable.**	• ¿Está instalada la máquina en un lugar afectado por vibraciones?<br>• ¿Está inclinada la plataforma?<br>⇨Mantenga la plataforma en posición horizontal. (☞ páginas 132 y 133)<br>• ¿Hay algún objeto atascado en los espacios de la plataforma?<br>⇨Retire cualquier objeto que esté atascado en los espacios.
**Sección de visualización**	**No aparece ninguna indicación en la pantalla después de conectar la alimentación eléctrica.**	• Compruebe que la fuente de alimentación está conectada correctamente.
	**Se visualiza** ––––– **.**	• El peso que se desea medir excede el margen de medición.

Síntoma		Comprobar
**Impresora**	**El papel se ha acabado**<br>P-End	• No se ha introducido papel en la impresora.<br>⇨Introduzca papel.<br>⇨Si la impresora no está en uso, pulse CE y vuelva a realizar los ajustes iniciales.
	**La tapa de la impresora está abierta**<br>C.OPEn	• La tapa de la impresora está abierta.<br>⇨Ciérrela correctamente.<br>⇨Compruebe que el papel de la impresora no se esté alimentando de forma oblicua.
	**El papel no sale.**	**Compruebe los ajustes.**<br>• ¿Se ha seleccionado 0 para el número de hojas a imprimir en “selección del número de hojas a imprimir?”<br>⇨Pulse 1 – 3. (☞ página 139)<br>• La impresora puede tener un desperfecto.<br>⇨Póngase en contacto con el distribuidor que le vendió el producto.
	**El papel sale, pero la información no se imprime.**	• ¿Se ha introducido el papel al revés en la impresora?<br>⇨Introduzca el papel correctamente (☞ página 135)<br>• La impresora puede tener un desperfecto.<br>⇨Póngase en contacto con el distribuidor que le vendió el producto.

Nota

- Si aparece un error distinto de los descritos anteriormente, apague la unidad y vuelva a realizar la medición.
  Si aparece un mismo error repetidamente, póngase en contacto con nuestro centro de atención al cliente.

- Especificaciones

Normas de comunicación	USB
Método de comunicación	Método de comunicación asincrónica
Velocidad de la señal	9600 bps
Longitud de bits de datos	8 bits
Paridad	NINGUNA
Bit de parada	1 bit
Control de flujo	NINGUNA
Terminador	CR+LF

Nota

- En la parte trasera de la unidad, se encuentran ubicados un conector USB (hembra de 4 clavijas y tipo B).
- Los cables de conexión no viene incluidos, utilice los suyos según proceda.
  USB: (macho) de 4 clavijas y tipo A - (macho) de 4 clavijas y tipo B.
- Vous devez installer sur votre PC le pilote nécessaire, à télécharger depuis http://www.tanita.com.

- Datos de transmisión

⚠PRECAUCIONES La transmisión de datos se efectúa inmediatamente después de realizar las mediciones sea cual sea el tipo de equipo receptor (ordenador personal, etc.). Por tanto el equipo receptor ha de estar preparado antes de las mediciones para recibir los datos.

(1) Formato de datos de salida

Nota

- Les données respectives sont séparées par des virgules (,).
- El terminador (fin de los datos) es CR (código ASCII 0DH), LF (código ASCII 0AH). Un 0 aquí corresponde a cero.
- Les données de contrôle pour les éléments 1 – 4 sont pour des extensions. Elles ne sont pas utilisées actuellement et le côté réception peut donc les ignorer.
- En raison de ses spécifications, le pèse-personne sortira un signal non pertinent approximativement 0,1 secondes après la sortie de données. Assurez-vous que ce signal est ignoré et ne l'importez pas.

Datos completos del cuerpo entero

Modelo	MO	"XXXXXX"
Número de serie	SN	"XXXXXXXX"
Número de ID	ID	"XXXXXXXXXX"
Fecha	Dt	"mm/dd/yyyy"
Hora	TI	"hh:mm"
Tipo corporal	Bt	0or2
Sexo	GE	1or2
Edad	AG	XX
Altura	Hm	XXX.X
Peso de la ropa	Pt	XX.X
Peso	Wk	XXX.X
% grasa	FW	XX.X
Masa grasa	fW	XXX.X
Masa libre de grasa	MW	XXX.X
Masa muscular	mW	XXX.X
Puntuación de masa muscular del cuerpo entero	sW	XX
Masa ósea	bW	XXX.X
Agua total en el cuerpo	wW	XXX.X
Índice de agua corporal	ww	XX.X
Índice masa corporal	MI	XXX.X
Peso estándar	Sw	XXX.X
Obesidad	OV	XXX.X
Índice de grasa visceral	IF	XX
Índice de metabolismo basal (kJ)	rb	XXXXX
Índice de metabolismo basal (kcal)	rB	XXXXX
Análisis metabolismo basal	rJ	XX
Edad metabólica	rA	XX
Target% de grasa corporal	gF	XX
Predicción de peso	gW	XXX.X
Predicción de masa grasa	gf	XXX.X
Grasa de ganar / perder	gt	XXXXX.X
Impedancia (50kHz)	ZF	XXXX.X
Suma de comprobación	CS	XX

(2) Datos de salida

<modo kg / cm>

Elemento	Encabezado	Formato	Índice	Orden de salida			
				Monitor de composición corporal			Báscula
				Adulto	Atleta	Niño	
Datos de control	{0		Fijado a 2 bytes	1	1	1	1
Datos de control	˜0		Fijado a1 byte	2	2	2	2
Datos de control	˜1		Fijado a1 byte	3	3	3	
Datos de control	˜2		Fijado a1 byte	4	4	4	
Modelo	MO	"XXXXXX"	Fijado a 8 bytes ("SC-331")	5	5	5	3
Número de serie	SN	"XXXXXXXX"	Fijado a 10 bytes	6	6	6	4
Número de ID	ID	"XXXXXXXXXX"	Fijado a 12 bytes	7	7	7	5
Fecha	Dt	"mm / dd / yyyy"	Fijado a 12 bytes ("mm / dd / yyyy")	8	8	8	6
Hora	Tl	"hh : mm"	Fijado a 7 bytes	9	9	9	7
Tipo corporal	Bt	0 or 2	Fijado a1 byte (0:Estándar 2:Atleta)	10	10	10	
Sexo	GE	1 or 2	Fijado a1 byte (1:Masculino 2:Femenino)	11	11	11	
Edad	AG	XX	Puede variar de 1 a 2 bytes	12	12	12	
Altura	Hm	XXX.X	Puede variar de 4 a 5 bytes (cm)	13	13	13	
Peso de la ropa	Pt	XX.X	Puede variar de 3 av 4 bytes (unidades de 0,1kg)	14	14	14	8
Peso	Wk	XXX.X	Puede variar de 3 a 5 bytes (unidades de 0,1kg)	15	15	15	9
% grasa	FW	XX.X	Puede variar de 3 a 4 bytes (unidades de 0,1%)	16	16	16	
Masa grasa	fW	XXX.X	Puede variar de 3 a 5 bytes (unidades de 0,1kg)	17	17	17	
Masa libre de grasa	MW	XXX.X	Puede variar de 3 a 5 bytes (unidades de 0,1kg)	18	18	18	
Masa muscular	mW	XXX.X	Puede variar de 3 a 5 bytes (unidades de 0,1kg)	19	19		
Puntuación de masa muscular del cuerpo entero	sW	XX	Puede variar de 1 a 2 bytes (1 a 24)	20	20		
Masa ósea	bW	XXX.X	Puede variar de 3 a 5 bytes (unidades de 0,1kg)	21	21		
Agua total en el cuerpo	wW	XXX.X	Puede variar de 3 a 5 bytes (unidades de 0,1kg)	22	22		
Índice de agua corporal	ww	XX.X	Fijado a 4 bytes	23	23		
Índice masa corporal	MI	XXX.X	Puede variar de 3 a 5 bytes (unidades de 0,1)	24	24	19	
Peso estándar	Sw	XXX.X	Puede variar de 3 a 5 bytes (unidades de 0,1kg)	25			
Obesidad	OV	XXX.X	Puede variar de 3 a 5 bytes (unidades de 0,1%)	26			
Índice de grasa visceral	IF	XX	Puede variar de 1 a 2 bytes	27	25		
Índice de metabolismo basal (kJ)	rb	XXXXX	Puede variar de 1 a 5 bytes (unidades de 1kJ)	28	26		
Índice de metabolismo basal (kcal)	rB	XXXXX	Puede variar de 1 a 5 bytes (unidades de 1kcal)	29	27		
Análisis metabolismo basal	rJ	XX	Puede variar de 1 a 2 bytes (1 a 24)	30	28		
Edad metabólica	rA	XX	Fijado a 2 bytes	31	29		
Target% de grasa corporal	gF	XX	Puede variar de 1 a 2 bytes (4 a 55)	32	30	20	
Predicción de peso	gW	XXX.X	Puede variar de 3 a 5 bytes (unidades de 0,1kg)	33	31	21	
Predicción de masa grasa	gf	XXX.X	Puede variar de 3 a 5 (unidades de 0,1kg)	34	32	22	
Aumento de grasa / pérdida	gt	XXXXX.X	Puede variar de 3 a 7 (unidades de 0,1kg)	35	33	23	
Impedancia (50kHz)	ZF	XXXX.X	Puede variar de 5 a 6 (unidades de 0,1)	36	34	24	
Suma de comprobación	CS	XX	Fijado a 2 bytes	37	35	25	10

<modo lb / ft-in>

Elemento	Encabezado	Formato	Índice	Orden de salida			
				Monitor de composición corporal			Báscula
				Adulto	Atleta	Niño	
Datos de control	{0		Fijado a 2 bytes	1	1	1	1
Datos de control	˜0		Fijado a1 byte	2	2	2	2
Datos de control	˜1		Fijado a1 byte	3	3	3	
Datos de control	˜2		Fijado a1 byte	4	4	4	
Modelo	MO	"XXXXXX"	Fijado a 8 bytes ("SC-331")	5	5	5	3
Número de serie	SN	"XXXXXXXX"	Fijado a 10 bytes	6	6	6	4
Número de ID	ID	"XXXXXXXXXX"	Fijado a 12 bytes	7	7	7	5
Fecha	Dt	"mm / dd / yyyy"	Fijado a 12 bytes ("mm / dd / yyyy")	8	8	8	6
Hora	Tl	"hh : mm"	Fijado a 7 bytes	9	9	9	7
Tipo corporal	Bt	0 or 2	Fijado a1 byte (0:Estándar 2:Atleta)	10	10	10	
Sexo	GE	1 or 2	Fijado a1 byte (1:Masculino 2:Femenino)	11	11	11	
Edad	AG	XX	Puede variar de 1 a 2 bytes	12	12	12	
Altura	Hi	XX.X	Puede variar de 4 a 5 bytes (in)	13	13	13	
Peso de la ropa	Pt	XX.X	Puede variar de 3 av 4 bytes (unidades de 0,2lb)	14	14	14	8
Peso	Wp	XXX.X	Puede variar de 3 a 5 bytes (unidades de 0,2lb)	15	15	15	9
% grasa	FW	XX.X	Puede variar de 3 a 4 bytes (unidades de 0,1%)	16	16	16	
Masa grasa	fW	XXX.X	Puede variar de 3 a 5 bytes (unidades de 0,2lb)	17	17	17	
Masa libre de grasa	MW	XXX.X	Puede variar de 3 a 5 bytes (unidades de 0,2lb)	18	18	18	
Masa muscular	mW	XXX.X	Puede variar de 3 a 5 bytes (unidades de 0,2lb)	19	19		
Puntuación de masa muscular del cuerpo entero	sW	XX	Puede variar de 1 a 2 bytes (1 a 24)	20	20		
Masa ósea	bW	XXX.X	Puede variar de 3 a 5 bytes (unidades de 0,2lb)	21	21		
Agua total en el cuerpo	wW	XXX.X	Puede variar de 3 a 5 bytes (unidades de 0,2lb)	22	22		
Índice de agua corporal	ww	XX.X	Fijado a 4 bytes	23	23		
Índice masa corporal	MI	XXX.X	Puede variar de 3 a 5 bytes (unidades de 0,1)	24	24	19	
Peso estándar	Sw	XXX.X	Puede variar de 3 a 5 bytes (unidades de 0,2lb)	25			
Obesidad	OV	XXX.X	Puede variar de 3 a 5 bytes (unidades de 0,1%)	26			
Índice de grasa visceral	IF	XX	Puede variar de 1 a 2 bytes	27	25		
Índice de metabolismo basal (kJ)	rb	XXXXX	Puede variar de 1 a 5 bytes (unidades de 1kJ)	28	26		
Índice de metabolismo basal (kcal)	rB	XXXXX	Puede variar de 1 a 5 bytes (unidades de 1kcal)	29	27		
Análisis metabolismo basal	rJ	XX	Puede variar de 1 a 2 bytes (1 a 24)	30	28		
Edad metabólica	rA	XX	Fijado a 2 bytes	31	29		
Target% de grasa corporal	gF	XX	Puede variar de 1 a 2 bytes (4 a 55)	32	30	20	
Predicción de peso	gW	XXX.X	Puede variar de 3 a 5 bytes (unidades de 0,2lb)	33	31	21	
Predicción de masa grasa	gf	XXX.X	Puede variar de 3 a 5 (unidades de 0,2lb)	34	32	22	
Aumento de grasa / pérdida	gt	XXXXX.X	Puede variar de 3 a 7 (unidades de 0,2lb)	35	33	23	
Impedancia (50kHz)	ZF	XXXX.X	Puede variar de 5 a 6 (unidades de 0,1)	36	34	24	
Suma de comprobación	CS	XX	Fijado a 2 bytes	37	35	25	10

E
Cuando sea necesario
(Conexión a un ordenador personal)

Medición de la composición corporal mediante el método BIA.

Introducción

Este equipo proporciona valores estimados para cada valor medido de porcentaje de grasa corporal, masa de grasa, masa libre de grasa, masa muscular y masa ósea mediante el método DXA para Japón, así como un valor estimado para el valor medido de agua corporal total mediante el método de dilución que utiliza un análisis de impedancia bioeléctrica (método BIA).
Para realizar una medición, se debe seleccionar un modo según el tipo corporal.
1) Estándar (para 5 a 99 años de edad)
2) Atlético (para atletas que se ejercitan más que el común de las personas)
La fiabilidad de la medición de la composición corporal de atletas, etc., que tienen una composición distinta al común de las personas, puede mejorarse dividiendo el modo de medición según cada tipo corporal.

- Principios de medición de la composición de estructura corporal

AIB es un método para medir la composición corporal – masa grasa, masa muscular estimada, etc. – mediante la medición de la impedancia bioeléctrica en el cuerpo. La grasa que se encuentra en el cuerpo no es un buen conductor de electricidad, mientras que el agua, cuya mayor parte se encuentra en los músculos, es un conductor eficaz de la electricidad. El grado de dificultad con el que la electricidad se transmite a través de una sustancia se conoce como resistencia eléctrica y el porcentaje de grasa y otros componentes corporales puede inferirse a partir de la medición de esta resistencia. El Analizador de la Composición Corporal de Tanita mide la composición corporal utilizando una fuente de corriente continua que genera una corriente de alta frecuencia (50kHz, 90µA).

- ¿Qué es el método DXA?

La absorciometría radiológica dual (DXA) se diseñó en un principio para medir el contenido mineral de los huesos, pero en el modo de exploración mediante escáner del cuerpo entero el porcentaje de grasa corporal, la masa grasa y la masa libre de grasa de determinadas partes del cuerpo (brazos, piernas, tronco) también puede medirse. La imagen de abajo muestra un ejemplo de los resultados de la medición de la composición corporal obtenidos mediante la DXA.

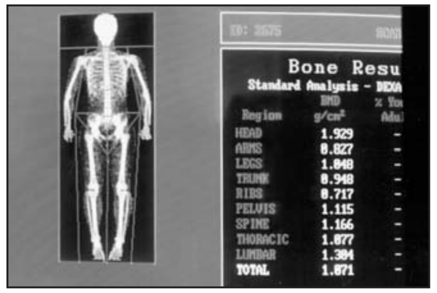

Resultados de la medición de la composición corporal obtenidos mediante la DXA (Lunar Co., Ltd; DPX-L)

- ¿Qué es el método de dilución?

En el método de dilución se utiliza una cantidad conocida de una sustancia etiquetada y se mide la concentración en equilibrio que se diluye uniformemente para obtener la cantidad total de disolvente que diluye a la sustancia etiquetada.
Para medir el agua corporal total (ACT), generalmente se utiliza óxido de deuterio (D_2O) como sustancia etiquetada. El óxido de deuterio utiliza el agua corporal total general como medio de dilución, lo que permite obtener el agua corporal total. Para obtener la cantidad de fluido extracelular se utiliza bromuro de sodio (NaBr) como sustancia etiquetada. Se dice que el bromuro (Br) no penetra al interior de las células y que utiliza el fluido extracelular como medio de dilución.

- ¿Qué es la grasa visceral?

El tejido adiposo visceral (VAT) es la grasa que se acumula en la cavidad abdominal y alrededor de los órganos internos. Se dice que el VAT es más susceptible de causar enfermedades relativas al estilo de vida que el tejido adiposo subcutáneo (SCAT). Por ello, conocer y revisar periódicamente el riesgo de acumulación de VAT es un valioso consejo para la prevención de enfermedades relativas al estilo de vida.

Tanita ha desarrollado una tecnología para la medición del riesgo de acumulación de VAT por análisis de impedancia bioeléctrica (BIA) mediante comparación con el análisis de imágenes aplicadas a las imágenes de resonancia magnética, además de la tecnología tradicional para la medición de la grasa corporal. El riesgo de acumulación de VAT se calcula mediante la estimación del área VAT por el método BIA en base al procesado de imágenes de resonancia magnética. Este método tiene una correlación superior a la estimación de riesgo de acumulación de VAT sobre el índice de masa corporal o la circunferencia abdominal (circunferencia en la cintura), permitiendo una estimación que se corresponde de una forma más precisa con cada individuo.
* El área VAT mediante resonancia magnética se calcula con la realización del procesado de imágenes de la sección transversal de las regiones de las vértebras lumbares L4-L5.

(Imagen 1 – Imagen 3: Resultados de la investigación realizada por N.Y Columbia University y Jikei University publicados por la Asociación norteamericana para el estudio de la obesidad (NAASO) en 2004)

- Factores que producen errores de medición

En el método BIA se mide la impedancia y se calcula la composición corporal a partir del valor obtenido. Se sabe que la impedancia cambia en una cantidad correspondiente al agua corporal total que ocupa aproximadamente el 60% del peso y que su distribución y temperatura cambian. Por esta razón, ya sea para fines de investigación para realizar mediciones que se repiten diariamente, las condiciones de medición deben mantenerse constantes. La adopción de la tecnología de reactancia permite obtener mediciones de una estabilidad nunca antes vista; sin embargo, los cambios de temperatura y distribución del agua corporal total o de volumen de flujo sanguíneo en las extremidades debido a ejercicios, baños, etc., afectan los resultados de las mediciones debido a que la resistencia eléctrica del cuerpo también cambia.
Por lo tanto, se recomienda efectuar las mediciones en las condiciones siguientes para obtener resultados estables.

1) Después de 3 horas de levantarse, habiendo realizado las actividades diarias normales de este período. (La impedancia se mantiene en un nivel alto si usted permanece sentado después de levantarse o si sólo se mueve en un coche, etc.)
2) Cuando hayan transcurrido 3 horas o más tiempo después de comer. (2 a 3 después de comer, la impedancia tiende a disminuir).
3) Cuando hayan transcurrido 12 horas o más tiempo después de hacer ejercicio. (Dependiendo del tipo e intensidad del ejercicio, la impedancia no tiende a cambiar de forma estable).
4) Vacíe la vejiga antes de realizar la medición.
5) En caso de mediciones repetidas, realice las mediciones a la misma hora si es posible. (Al mismo tiempo que se realiza la medición de peso, las mediciones pueden ser más estables si se realizan a la misma hora del día).

Se pueden obtener valores muy estables realizando las mediciones en las condiciones descritas anteriormente.
En el desarrollo de este equipo se establecieron 6 puntos como condiciones para la ecuación de regresión.

1) Prohibición de ingerir alcohol hasta 12 horas antes de la medición.
2) Prohibición de hacer ejercicio excesivo hasta 12 horas antes de la medición.
3) Prohibición de comer y beber en exceso en el día anterior a la medición
4) Prohibición de comer y beber hasta 3 horas antes de la medición.
5) Evitar realizar las mediciones durante el período menstrual (mujeres)

2) Variaciones interdiarias

Los diagramas que se muestran debajo incluyen ejemplos de mediciones reales sobre variaciones interdiarias. Se realizó un estudio para determinar el grado de variación de la impedancia entre los pies durante el estado de deshidratación. Los primeros dos días representan la rutina cotidiana normal del sujeto, mientras que en los dos días posteriores se indujo el estado de deshidratación mediante una sauna.

Durante la rutina cotidiana no se registraron variaciones interdiarias de importancia en cuanto al peso corporal, la impedancia entre los pies o el porcentaje de grasa corporal. Sin embargo, durante el estado de deshidratación se registró una disminución del peso corporal de 1kg y un aumento de la impedancia entre los pies de aproximadamente 15Ω, durante el

Fluctuaciones intradiarias (Peso)

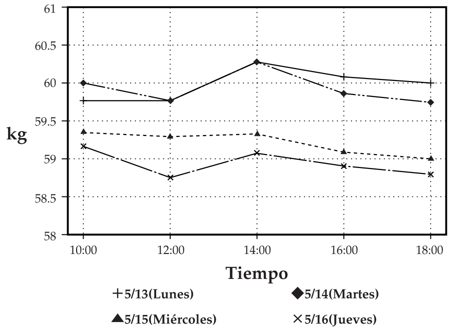

Fluctuaciones intradiarias (Valor de la impedancia)

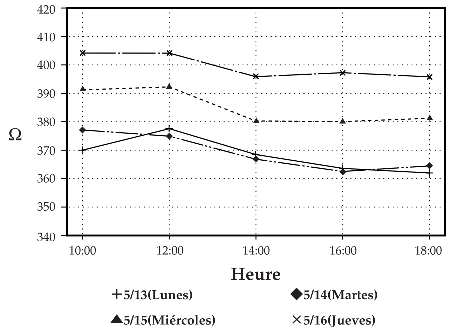

primer día en el que se indujo la deshidratación, y de 30-35Ω el segundo día. Como consecuencia de ello, el porcentaje de grasa corporal
aumentó el 1% el primer día en el que se indujo la deshidratación y el 1,5-2% el segundo día.
Como se ha mencionado anteriormente, la impedancia aumenta cuando el peso corporal disminuye (por ejemplo, debido a la deshidratación) y disminuye cuando el peso corporal aumenta debido a excesos en el consumo de comida y bebida. La variación interdiaria de la impedancia es por lo tanto inversamente proporcional al cambio del peso corporal.
Estas variaciones interdiarias son causadas por factores como:

1) Aumentos temporales del peso corporal (agua corporal total) originados por comer o beber en exceso.
2) Deshidratación debida a sudoración abundante durante la realización de ejercicio intenso.
3) Deshidratación debida al consumo de alcohol o al uso de diuréticos.
4) Deshidratación debida a la sudoración abundante durante la toma de saunas, etc.

Por consiguiente, se recomienda proporcionar al sujeto instrucciones para ayudar a eliminar estas causas cuando se requieran mediciones precisas.

La nueva fórmula de regresión para calcular la Tasa Metabólica Basal (MB)

Hace tiempo que los médicos y los especialistas en nutrición afirman que "La Tasa Metabólica Basal (MB) depende más de la Masa Libre de Grasa (MLG) que del peso corporal" (es decir, las personas que tengan un peso corporal determinado con mayor MLG tendrán una mayor MB) y que la evaluación de la composición corporal debería estimarse a partir de la MLG. Además, en ocasiones las fórmulas de estimación sencillas que permiten la realización de cálculos a partir de la altura, el peso y la edad, sin evaluar la composición corporal, suponían un problema al proporcionar resultados de evaluaciones de la MB excesivamente altos a personas obesas con un peso corporal elevado y, a la inversa, resultados excesivamente bajos a atletas musculosos, aunque estos casos no fuesen tan numerosos. En la actualidad, la fórmula de recurrencia para la estimación de la MB desarrollada por Tanita, el fabricante de analizadores de la composición corporal, basándose en sus investigaciones, funciona mediante el análisis de regresión múltiple utilizando la MLG y proporciona un grado de precisión más elevado respecto a las diferencias individuales de la composición corporal. Para obtener la MB, se midió el metabolismo respiratorio durante el reposo (Gasto Energético en Reposo: GER) mediante un analizador de los gases respiratorios y basándose en estos datos se creó la fórmula de recurrencia para la realización de estimaciones.

<Gráfica 1> La relación entre el Gasto Energético en Reposo (GER) según el análisis de los gases respiratorios y del peso, MGL
(Presentado en la Semana de la Nutrición "Nutrition Week", celebrada en San Diego en el 2002)

Como se muestra en la Gráfica 1: el GER (MB) mantiene una relación más afín con la MLG que con el peso corporal, además se observa una diferencia en las tendencias de distribución entre los hombres y las mujeres. Por ello, entendemos que, en un principio, los cálculos deberían realizarse a partir de la MLG en vez de utilizar la fórmula anterior centrada en la relación con el peso.

MLG vs GER

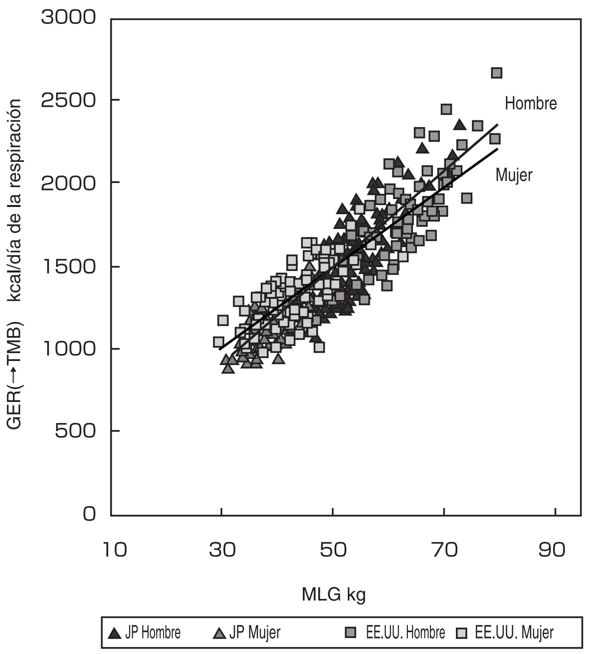

Peso vs GER

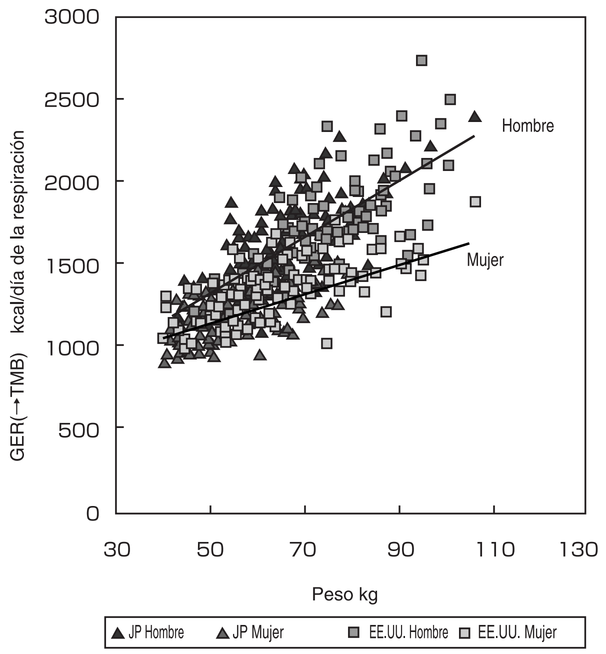

<Gráfica 2> Comparación de los valores de la MB según el modelo de regresión múltiple y el análisis de la respiración de TANITA.
(Presentado en la Semana de la Nutrición "Nutrition Week", celebrada en San Diego en el 2002)

La fórmula de regresión actual de la MB es una fórmula que se basa en el principio de utilizar el valor de la MLG resultante de la medición de la composición corporal según el AIB. Se muestra una estrecha relación entre el valor de la MB basado en el análisis de la respiración GER o R=0,9 (p<0,0001). Estos resultados fueron presentados en la Primera Semana Annual de la Nutrición "First Annual Nutrition Week" (American College of Nutrition, American Society for Clinical Nutrition, American Society for Parenteral and Enteral Nutrition, North American Association for the Study of Obesity) celebrada en San Diego en el 2002.

NOTA: Este modelo ha sido calibrado para usuarios con edades comprendidas entre los 18 y los 84 años. Los usuarios cuyas edades se encuentren fuera de este margen de edad podrían obtener lecturas imprecisas.

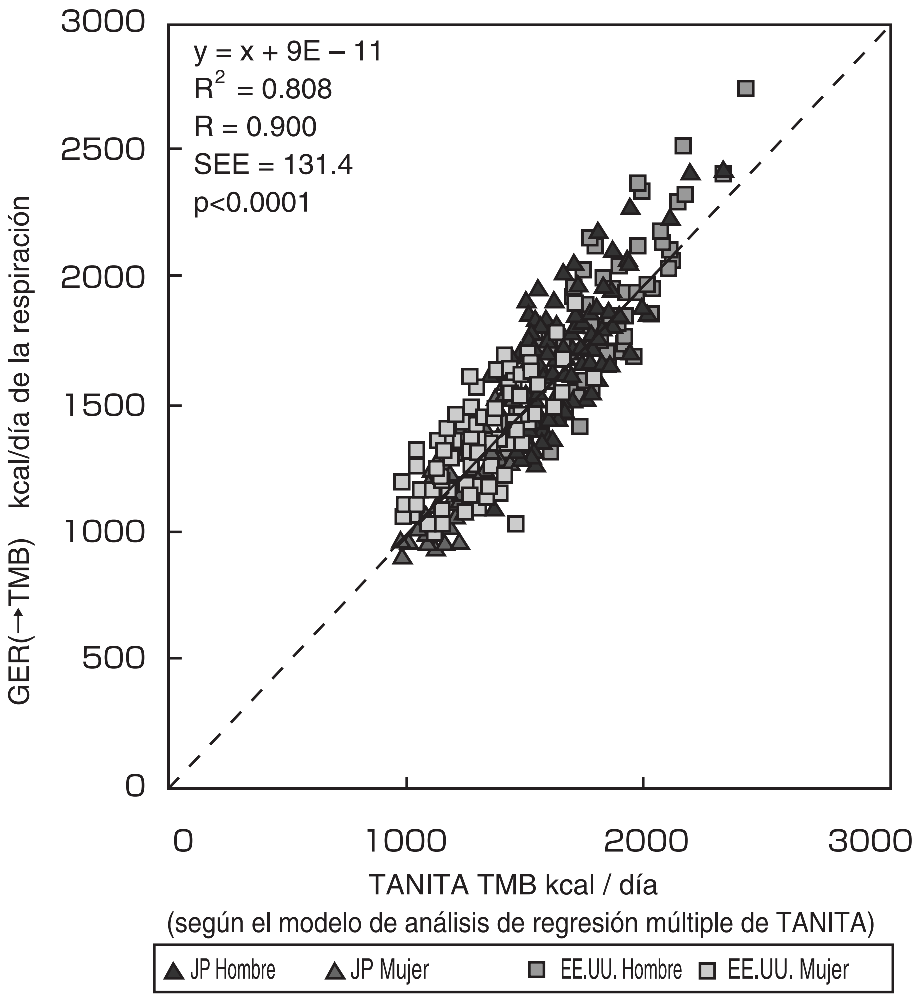

Especificaciones

Modelo		SC-331S	
Fuente de suministro eléctrico		Adaptador AC (incluido) Center Minus	
		Voltaje de entrada: 100-240 VAC 50/60 Hz 1,5A Voltaje de salida: 7 VDC	
		Corriente de régimen: 4 A Voltaje de entrada en vacío: 7VDC	
Consumo eléctrico		28 W	
Medición de impedancia	Sistema de medición	Análisis de impedancia bioeléctrica tetra polar	
	Frecuencia de medición	50 kHz	
	Corriente de medición	90 µA	
	Material de electrodos	Almohadillas para los pies de acero inoxidable activadas mediante contacto por presión	
	Estilo de medición	Entre ambos pies	
	Rango de medición	150 - 1200Ω	
	Precisón al primer calibrado	± 2%	
Medición de peso	Sistema de medición	Célula de carga indicadora de tensión	
	Capacidad máxima / graduación mínima	270 kg / 0,1 kg<br>600 lb / 0,2 lb	
	Precisón al primer calibrado	± 0,2 kg / ± 0,4 lb	
Elementos de información de entrada	Peso de la ropa	0 - 10 kg / incrementos de 0,1 kg	incrementos de 0 - 20 lb / 0,2 lb
	Sexo	Hombre / mujer	
	Tipo de estructura corporal	Estándar (5 - 99 años) / Atlético (18 - 99 años)	
	Edad	5 - 99 años de edad / incrementos de 1 año	
	Altura	90 – 249,9 cm / incrementos de 0,1 cm	3 ft - 7 ft 11,5 in / incrementos de 0.5 in
	Porcentaje de grasa corporal objetivo	4 - 55 %	

Elementos de información de salida	Display	Porcentaje de grasa corporal objetivo	4 - 55 %	
		Peso	0 - 270 kg / incrementos de 0,1 kg	600lb / incrementos de 0,2 lb
		Sexo	Hombre / mujer	
		Tipo de estructura corporal	Estándar / Atlético	
		Edad	5 - 99 años de edad / incrementos de 1 año	
		Altura	90 - 249,9 cm / incrementos de 0,1 cm	3 ft - 7 ft 11,5 in / incrementos de 0,5 in
		FAT%	3 - 75% / 0.1% increments	
	Impresión	Logo	Logo de TANITA (240 × 64 puntos)	
		Nombre del modelo	BC-420MA	
		Fecha y hora	2005 / 1 / 1 - 2099 / 12 / 31	
		Nº de serie	00000000 - 99999999	
		ID	0000000000 - 9999999999	
		Tipo de estructura corporal	Estándar (5 - 99 años) / Atlético (18 - 99 años)	
		Sexo	Hombre / mujer	
		Edad	5 - 99 años de edad / incrementos de 1 año	
		Altura	90 - 249,9 cm / incrementos de 0,1 cm	3 ft - 7 ft 11,5 in / incrementos de 0,5 in
		Peso de la ropa	0 - 10 kg / incrementos de 0,1 kg	0 - 20 lb / incrementos de 0,2 lb
		Peso	0 - 270 kg / incrementos de 0,1 kg	600lb / incrementos de 0,2 lb
		Porcentaje de grasa	3 - 75% / incrementos de 0,1%	
		Masa corporal	incrementos de 0,1 kg	incrementos de 0,2 lb
		Masa Magra	incrementos de 0,1 kg	incrementos de 0,2 lb
		Masa muscular	incrementos de 0,1 kg	incrementos de 0,2 lb
		Agua Total	incrementos de 0,1 kg	incrementos de 0,2 lb
		% Agua Total	15 - 85% / incrementos de 0,1%	
		Masa ósea prevista	incrementos de 0,1 kg	incrementos de 0,2 lb
		MB	incrementos de 1 kJ / incrementos de 1kcal	
		Edad metabólica	incrementos de 1 año (12 - 90 años)	
		Nivel de grasa visceral	incrementos de 1 nivel (nivel 1 - 59)	
		BMI	incrementos de 0,1	
		Peso previsto	incrementos de 0,1 kg	incrementos de 0,2 lb
		Rango deseable	3 - 75% / incrementos de 0,1 kg	
		Gráfico de porcentaje de grasa		
		Gráfico de BMI		
		Gráfico de nivel de grasa visceral		
		Gráfico de masa muscular		
		Gráfico de MB		
		Evaluación física		
		Resistencia	150 - 1200Ω	
Display			Pantalla LCD de 3 filas y 5 dígitos	
Interfaz de datos de salida			RS-232C (conector hembra de 9 pins D-sub)	
Rango de temperature de uso			0 - 35˚C	
Humedad relativa			30 - 80% (sin condensación)	
Peso del equipo (versión de display remoto)			6,8 kg	15,0 lb
Peso del equipo (versión de montaje en columna)			12,1kg	26,7 lb
Tamaño	Plataforma de pesaje		372 × 375 × 101 mm (14,6 × 14,8 × 4,0 in)	
	Altura (versión de montaje en columna)		1024 mm (40,3in)	

USA y Canada

Comisión Federal de Comunicaciones y Canadá CIEM comentario

Este equipo ha sido probado y cumple con los límites para un dispositivo digital de Clase B, según la Parte 15 de las normas FCC y canadienses ICES-003. Estos límites están diseñados para proporcionar una protección razonable contra las interferencias perjudiciales en una instalación residencial. Este equipo genera, utiliza y puede irradiar energía de radiofrecuencia y, si no se instala y utiliza de acuerdo con las instrucciones, puede causar interferencias perjudiciales en las comunicaciones de radio. Sin embargo, no hay garantía de que no se produzcan interferencias en una instalación en particular. Si este equipo causa interferencias perjudiciales en la interferencia de radio o televisión, lo que puede determinarse encendiéndolo y apagándolo, se recomienda al usuario que intente corregir la interferencia mediante una o más de las siguientes medidas:

- Cambiar la orientación o ubicación de la antena receptora.
- Aumentar la separación entre el equipo y el receptor.
- Conectar el equipo a un tomacorriente en un circuito diferente de aquel al que está conectado el receptor.
- Consultar con el distribuidor o con un radio o televisión con experiencia con experiencia.

Modificaciones

La FCC exige que el usuario sea notificado de que cualquier cambio realizado en este dispositivo que no sea aprobado expresamente por Tanita Corporation podría invalidar el usuario para utilizar el equipo.

Eliminación

Este equipo es un dispositivo electrónico. No lo deseche junto con los residuos generales; cumpla con las normas regionales de eliminación de equipos eléctricos.

<U.S.A. representative>

TANITA Corporation of America, Inc.

2625 South Clearbrook Drive, Arlington Heights, Illinois 60005, U.S.A. TEL: 1-847-640-9241 FAX: 1-847-640-9261 http://www.tanita.com

<Manufacturer>

TANITA Corporation

1-14-2, Maeno-cho, Itabashi-ku, Tokyo 174-8630 , Japan TEL: +81 (0) 3-3968-7048 FAX: +81 (0) 3-3967-3766 http://www.tanita.co.jp

TANITA Europe B.V.
Hoogoorddreef 56-E,
1101 BE Amsterdam, the Netherlands
TEL: +31-20-560-2970
FAX: +31-20-560-2988
http://www.tanita.eu

TANITA Health Equipment H.K.LTD.
Unit 301-303, Wing On Plaza, 3/F., 62 Mody Rord,
Tsimshatsui East, Kowloon, Hong Kong
TEL: +852-2834-3917
FAX: +852-2838-8667
www.tanita.asia

TANITA India Private Limited
A-502, Mittal Commercia, Off. M.V. Road(Andheri Kurla Road),
Marol, Andheri-East, Mumbai 400 059 INDIA
TEL: +91-22-3192-6107
FAX: +91-22-2859-9143
www.tanita.co.in

TANITA (Shanghai) Trading Co., Ltd.
Room 8005, 877 Huai Hai Zhong Lu, Shanghai,
The People's Republic of China
TEL: +86-21-6474-6803
FAX: +86-21-6474-7901
www.tanita.com.cn

Copyright © 2013 TANITA Corporation. All Rights Reserved.

Feb 2013.TY.C.SC3317601(3)
