# Supplementary material for: The effect of protein supplementation on body muscle mass and fat mass in post-bariatric surgery: a randomized controlled trial (RCT) study protocol
Source: Arch Public Health. 2018 Jan 22;76:7. doi: 10.1186/s13690-017-0252-2 (PMC5789587; doi:10.1186/s13690-017-0252-2)
Supplement: Supplementary file 2 — (PDF 1097 kb) [file 13690_2017_252_MOESM2_ESM.pdf]

نموذج الموافقة على الاشتراك ببحث علمي

RESEARCH CONSENT FORM

**1. Title of research**	**1. عنوان البحث**
Protein supplementation impact on body muscle mass and fat mass in Qataris post bariatric surgery, Randomized Controlled Trials (RCTs)	تأثير المكمّلات البروتينية على الكتلة العضلية و الدهنية لدى القطريين بعد جراحة علاج السمنة: دراسة بحثية منضبطة عشوائية
**2. Principal Investigator**	**2. الباحث الرئيسي**
Sahar Al Shammari, Hamad Medical Corporation	سحر الشمري، مؤسسة حمد الطبية
**3. Why are we inviting you to join this research?**	**3. لماذا ندعوك للإنضمام إلى هذا البحث؟**
The investigator and colleagues at Hamad Medical Corporation (HMC) – Bariatric department are conducting this research.<br><br>We are inviting you to join because you are Qatari patient with age between 18-45 years who has undergone a bariatric surgery recently at HMC.	الباحث وزملائه في مؤسسة حمد الطبية – قسم جراحات السمنة ينوون اجراء هذا البحث.<br><br>أنت مدعوا/ة للمشاركة لأنك مريض قطري في الفئة العمرية بين 18-45 سنة وقد أجريت عملية لعلاج السمنة مؤخرا بمؤسسة حمد الطبية.
**4. What should you know about this research?**	**4. ما الذي يجب أن تعرفه عن هذا البحث؟**
• We will explain the research to you<br>• Whether or not you join is your decision (you can accept or refuse no matter who is inviting you to participate)<br>• Please feel free to ask questions or mention concerns before deciding, or during or after the research<br>• You can say yes but change your mind later<br>• We will not hold your decision against you	• سنقوم بشرح البحث لك بشكل وافٍ<br>• قرار انضمامك للمشاركة بهذا البحث أو عدمه يعود لك (يمكنك قبول أو رفض المشاركة بغض النظر عن من يدعوك للمشاركة)<br>• لك مطلق الحرية بأن تسأل أي سؤال قبل اتخاذ قرارك, أو خلال أو بعد المشاركة بالبحث.<br>• إذا وافقت على المشاركة, بإمكانك أن تغير رأيك لاحقاً<br>• لن يستخدم قرارك ضدك بأي حال من الأحوال
**5. Who can you talk to?**	**5. مع من يمكنك التحدث؟**
If you have questions or concerns, or if you think the research has hurt you, talk to the research team at:<br>Ms. Sahar AL Shammari 70995505 - 44396792<br>If you have questions about your rights as a volunteer, or you want to talk to someone outside the research team, please contact:<br>• HMC Medical Research Centre at irb@hamad.qa	لطرح أية أسئلة أو مناقشة أي مخاوف, أو إذا كنت تعتقد أن البحث قد أضر/يضر بك, قم بالتحدث مع فريق البحث على:<br>44396792 – 70995505<br><br>إذا كان لديك أسئلة حول حقوقك كمشارك بالبحث، أو كنت ترغب في التحدث مع شخص من خارج فريق البحث، يرجى الإتصال ب:<br>• مركز الأبحاث, مؤسسة حمد الطبية, إيميل: irb@hamad.qa
**6. Why are we doing the research?**	**6. لماذا نقوم بهذا البحث؟**

نموذج الموافقة على الاشتراك ببحث علمي
RESEARCH CONSENT FORM

This study is to see the impact of the intervention in post bariatric patient in many sides like weight reduction and improvement of nutritional status in general. We would like to assess the effect of protein supplementation on changes in health parameters in Qatari patients post bariatric surgery. We will be assessing these changes by measuring few parameters like muscle and fat mass, body weight, proteins, Vit B12 and minerals like Magnesium and Zinc.	هذه الدراسة لمعرفة تأثير التدخل النوعي في مرضى بعد عملية علاج السمنة من عدة نواحي تشمل إنقاص الوزن و تحسين التغذية بشكل عام. نريد تقييم تأثير المكمّلات البروتينية على صحة المرضى القطريين بعد عملية علاج السمنة. سنقوم بتقييم هذه التغييرات عبر قياس بعض المؤشرات الحيوية مثل الكتلة العضلية و الدهنية، الوزن، فيتامين ب12 و المعادن مثل المغنيسيوم و الزنك.
**7. How long will the research take?**	**7. كم من الوقت سيستغرق هذا البحث؟**
We think that you will be in the research for 3 months starting from the first day post-operative.<br><br>We expect the research to last for 6 months.	نعتقد أنك ستكون في البحث لمدة 3 شهور من أول يوم بعد العملية.<br><br>نتوقع أن يستمر البحث لمدة 6 شهور.
**8. How many people will take part?**	**8. كم عدد الأشخاص الذين سيشاركون بهذا البحث؟**
We plan to study 80 people (post bariatric patients) from HMC. They will be divided in 2 groups equally.	نحن نخطط لدراسة 80 شخص (مرضى عمليات علاج السمنة) من مؤسسة حمد الطبية. سيتم تقسيمهم الى مجموعتين بالتساوي.
**9. What happens if you take part?**	**9. ما الذي سيحدث اذا قررت الاشتراك بهذا البحث؟**
If you agree to take part, you will be “randomized” into one of 2 study groups - One will be intervention group and one would be the control group. This trial will be a double-blinded study.<br><br>Randomization means that you are put into a group by chance. It is like flipping a coin. You will have a 50% chance of being place in a specific group. Neither you nor the researchers choose which group you will be in.<br><br>As a part of the standard care for post bariatric patients, all patients will receive nutritional counseling by a bariatric dietitian in a routine round, aiming that they will know the post-surgery diet stages and to not develop any nutritional deficiencies.<br><br>If you are from Intervention group:<br>1. You will receive nutritional counseling by a bariatric dietitian in a routine round, aiming that they will know the post-surgery diet stages and to not develop any nutritional deficiencies.<br>2. Before discharge from the hospital, you will have a store request for supplement and will be advised by the dietitian regarding its use (one can per day, over 3-5 intervals).	اذا وافقت على المشاركة، سوف يتم "اختيارك عشوائياً "في واحدة من مجموعات الدراسة – مجموعة التدخل و المجموعة الثاية للمقارنة.<br><br>التوزيع العشوائي يعني أن إلحاقك بأي مجموعة من مجموعات البحث سيتم عن طريق الصدفة. مثلاً برمي العملة النقدية أو القرعة. لا أنت ولا الباحثين ستتمكنون من اختيار المجموعة التي سوف تكون فيه.<br><br>كجزء من الرعاية القياسية للمرضى بعد عمليات علاج السمنة، يحصل جميع المرضى على استشارات للتغذية عبر أخصائي التغذية أثناء الجولة الروتينية و ذلك لإرشادهم للتغذية السليمة بعد العمليات و تجنب مضاعفات بسبب نقص التغذية.<br><br>اذا كنت في مجموعة التدخل:<br>1) ستحصل على إستشارات غذائية عن طريق أخصائي التغذية أثناء الجولة الروتينية و ذلك لإرشادهم للتغذية السليمة بعد العمليات و تجنب مضاعفات بسبب نقص التغذية.<br>2) ستستلم طلب موجه للمخزن لإستلام مكمّل غذائي بتركيز عالي للبروتين و بعض الإرشادات من أخصائي التغذية عن كيفية استعماله (عبوة واحدة يوميا على 3-5 فترات)<br>3) و تحتوي العبوة الواحدة للمكمّل (200 ملل) على 20 جرام بروتين، 250 كيلو كالوري و بعض العناصر الغذائية المهمة.<br>4) عند إستلام المكمّلات من مخزن المستشفى، سيقومون بتزويدك ببعض المعلومات الاضافية لضمان الاستخدام حسب متطلبات الدراسة.

3. Supplement contains (per 200 ml can) 20 g of protein, 250Kacl plus different micronutrient and macronutrient (Cubitan Protein, Nutricia, Netherlands).
4. When you collect your supplement package from the hospital store, they will provide you with instructions about the use the supplement to ensure protocol is followed.

If you are in the control group:
1. You will receive nutritional counseling by a bariatric dietitian in a routine round, aiming that they will know the post-surgery diet stages and to not develop any nutritional deficiencies.
2. Before discharge from the hospital, you will have a store request for supplement and will be advised by the dietitian regarding its use (one can per day over 3-5 intervals).
3. Following hospital discharge, you will receive supplement contains per can (200 ml), 0g protein, fat free, 100 kcal and enriched with electrolytes (preOp, Nutricia, Netherlands).
4. When you collect your supplement package from the hospital store, they will provide you with instructions about the use the supplement to ensure protocol is followed.

Measurements for both groups:
- Baseline measurement of body composition (fat mass and muscle mass), height and weight will be conducted on day one of the trial.
- Baseline blood test for total protein, albumin, Vit B12, Zink and Magnesium levels.
- All of the above measurements will be repeated at 1 and 3 month after surgery. Collection of blood samples at 1 & 3 months post the surgery is part of routine practice for post-bariatric surgery patients.

Study visits for both groups:
<u>One and 3 months after surgery (1 day each visit)</u>
a) Post-surgery dietary advice, to sustain a hypo caloric and protein-rich diet.
b) Anthropometric parameters for body composition (fat and muscle mass will be measured to assess healthy weight).
c) Give blood sample after an overnight fasting. All blood markers will be measured and calculated in the central laboratory in HGH.
d) Both groups will be asked about their supplement compliance to determine their eligibility to continue in study.
e) All randomized patients will be analyzed by intention to treat (ITT) analysis

إذا كنت في مجموعة المقارنة:
1) ستحصل على إستشارات غذائية عن طريق أخصائي التغذية أثناء الجولة الروتينية و ذلك لإرشادهم للتغذية السليمة بعد العمليات و تجنب مضاعفات بسبب نقص التغذية.
2) ستستلم طلب موجه للمخزن لإستلام مكمّل غذائي وبعض الإرشادات من أخصائي التغذية عن كيفية استعماله (عبوة واحدة يوميا على 3-5 فترات)
3) و تحتوي العبوة الواحدة للمكمّل (200 ملل) على20 جرام بروتين، خالي الدهون، 100 كيلو كالوري و بعض العناصر الغذائية المهمة.
4) عند إستلام المكمّلات من مخزن المستشفى، سيقومون بتزويدك ببعض المعلومات الاضافية لضمان الاستخدام حسب متطلبات الدراسة.

القياسات للمجموعتين:
- قياسات أولية لكتلة الجسم (العضلات و الدهون)، الوزن، الطول في اليوم الأول للدراسة
- تحليل دم أول لقياس مستوى البروتين، الزلال، فيتامين ب12' الزنك و المغنيسيوم.
- سيتم اعادة القياسات السابق ذكرها بعد شهر و 3 شهور عند نهاية المشاركة في الدراسة.

زيارات بحثية للمجموعتين:
<u>بعد شهر من الجراحة</u>
a) نصائح عن التغذية ما بعد العملية للمحافظة على نظام قليل السعرات و غني بالبروتينات.
b) قياسات لمؤشرات كتلة الجسم
c) عينة من الدم بعد صيام ليلة. سيتم عمل التحاليل في مخبرات مستشفى حمد
d) سيتم سؤال المشاركين في المجموعتين عن التزامهم بالمكمّلات لتحديد مواصلتهم في البحث
e) جميع المرضى الذين تم توزيعهم عشوائيا سيتم تحليلهم بطريقة "الية للعلاج" الخاصة. (ITT Analysis)

**10. Could the research be bad for you?**	**10. هل يمكن لهذا البحث أن يضرك؟**
The research does not pose any risk to the participants in the intervention and control group. The intervention doesn't have any side effect for any of the 2 groups.<br><br>You will not be included in the study if you have:<br>1) Any Renal or liver disease because that will affect protein or albumin level in body.<br>2) Past history of bariatric surgery<br>3) Baseline tests showing you are in need for protein supplements. In this case, you will be given the needed protein supplements per the standard care.<br>4) You are not willing to participate.	الاجراءات المتعلقة بهذا البحث لا تشكل خطراً على المشاركين في المجموعتين. لا توجد أي آثار جانبية للمكمّلات البروتينية.<br><br>لن تستطيع المشاركة في هذه الدراسة اذا كانت لديك:<br>1) أمراض في الكلى او الكبد و التي من شأنها التأثير على مستوى البروتين أو الزلال في الجسم<br>2) سجل تاريخي بعمليات السمنة<br>3) حاجة للمكمّلات البروتينية حسب الفحوصات الأولية. في هذه الحالة سيتم اعطائك هذه المكمّلات كجزء من الرعاية العادية لهذه الحالات.
**11. Could the research be good for you?**	**11. هل يمكن لهذا البحث أن يفيدك؟**
There are no benefits to you from joining this research. However, possible benefits to others include the importance of intervention to their health status and weight reduction. If the protein intervention proves to be beneficial, we will recommend this to the respective authorities for implementation.	لا توجد فوائد من انضمامكم لهذا البحث. ومع ذلك، فالفوائد المحتملة للآخرين تشمل معرفة جدوى التدخل النوعي لتحسين حالتهم الصحية و إنقاص الوزن. إذا ثبت وجود فوائد مباشرة للمكمّلات البروتينية، سنقوم برفع توصيات للجهات المختصة لتطبيقها.
**12. What happens to information about you?**	**12. ما الذي سيحدث للمعلومات عنك؟**
We will make efforts to secure information about you. This includes using a code to identify you in our records instead of using your name. We will not identify you personally in any reports or publications about this research.<br><br>We cannot guarantee complete secrecy, but we will limit access to information about you. Only people who have a need to review information will have access. These people might include:<br>• Members of the research team whose work is related to the research or to protecting your rights and safety<br>• Representatives of the Ministry of Public Health who make sure the study is done properly and that your rights and safety are protected<br>• Your doctors and nurses	سنحرص على حماية المعلومات المتعلقة بك. وهذا سيتضمن استخدام رمز للتعرف عليك في سجلاتنا بدلاً من استخدام اسمك. ونحن لن نحدد هويتك الشخصية في أي تقارير أو مطبوعات ناتجة من هذا البحث.<br><br>لا يمكننا ضمان السرية التامة, ولكننا سنحدّ من إمكانية الوصول إلى المعلومات المتعلقة بك وعنك. فقط الأشخاص الذين سيكونون بحاجة لمراجعة معلوماتك سيتمكنون من الوصول إليها. هؤلاء الأشخاص ممكن أن يكونوا:<br>• فريق البحث المرتبطة أعمالهم بهذا البحث او بحماية حقوقك وسلامتك<br>• ممثلي وزارة الصحة العامة في قطرالتي تتأكد من قيام الدراسة بالشكل الصحيح وتتأكد من حماية حقوقك وسلامتك.<br>• أطباؤك وطاقم التمريض
**13. What if you don't want to join?**	**13. ماذا لو كنت لا تريد المشاركة؟**

You can say no and we will not hold it against you.	يمكن ان ترفض المشاركه في البحث ولن يستخدم قرارك ضدك بأي حال من الأحوال.
**14. What if you join but change your mind?**	**14. ماذا لو انضممت الآن ولكن غيرت رأيك لاحقاً؟**
You can stop participating at any time and we will not hold it against you.<br><br>You may inform the research team through the available contact information and we will exclude the data we collected about you from this study.	يمكنك التوقف عن المشاركة بهذا البحث بأي وقت، ولن يستخدم قرارك ضدك بأي حال من الأحوال<br><br>إذا توقفت عن المشاركة بالبحث، يرجى الاتصال بنا لحذف المعلومات التي جمعناها عنك مسبقاً.
**15. What else should you know?**	**15. ما الذي يجب أن تعلمه أيضاً؟**
The research is approved by the Medical Research Center – HMC.<br><br>The researcher might stop this study at any time or decide to stop your participation in this study even if you want to continue. This could happen for the following reasons:<br>• If you did not take at least 80% of their intervention product amount per day, or<br>• If you did not take the intervention product (supplement) for more than 3 days per week.	تمت الموافقة على هذا البحث من مركز البحوث الطبية بمؤسسة حمد.<br><br>قد يقوم الباحث أو راعي البحث/مموله بوقف الدراسة أو انهاء مشاركتك في الدراسة في أي وقت، حتى لو كنت تريد الاستمرار .وهذا يمكن أن يحدث للاسباب التالية:<br>• عدم الالتزام بالمكمّلات بنسبة 80% على الاقل للجرعة اليومية<br>• عدم الالتزام بالمكمّلات لأكثر من 3 أيام في الاسبوع

نموذج الموافقة على الاشتراك ببحث علمي

RESEARCH CONSENT FORM

**Signature Page for Capable Adult**	**صفحة التوقيع للمشارك البالغ العاقل**
**Volunteer**	**المشارك**
I voluntarily agree to join the research described in this form.	أوافق طوعاً على الانضمام الى البحث المشروح في هذا النموذج
Printed Name of Volunteer	الاسم الكامل للمشارك بالبحث
Signature of Volunteer Date	التوقيع التاريخ
**Person Obtaining Consent**	**الشخص الحاصل على الموافقة**
I document that:<br>• *I (or another member of the research team) have fully explained this research to the volunteer.*<br>• *I have personally evaluated the volunteer's understanding of the research and obtained their voluntary agreement.*	أشهد أني:<br>• أنا (أو أحد أعضاء فريق البحث) قمنا بشرح البحث بشكل وافي للمشارك بالبحث<br>• قمت شخصياً بتقييم فهم المشارك بالبحث والحصول على موافقته/ها الطوعية.
Printed Name of Person Obtaining Consent	الاسم الكامل الشخص الحاصل على الموافقة
Signature of Person Obtaining Consent Date	التوقيع التاريخ
**Witness (if applicable)**	**الشاهد (عند الضرورة)**
I document that the information in this form (and any other written information) was accurately explained to the volunteer, who appears to have understood and freely given Consent to join the research.	أشهد أنه تم شرح المعلومات الواردة في هذا النموذج بدقة (وأية معلومات أخرى مكتوبة) للمشارك بالبحث. ,انه يبدو أنه قد فهم البحث وأن موافقته على الإنضمام إلى هذا البحث طوعية.
Printed Name of Witness	الاسم الكامل للشاهد
Signature of Witness Date	التوقيع التاريخ
