# Supplementary material for: The effect of protein supplementation on body muscle mass and fat mass in post-bariatric surgery: a randomized controlled trial (RCT) study protocol
Source: Arch Public Health. 2018 Jan 22;76:7. doi: 10.1186/s13690-017-0252-2 (PMC5789587; doi:10.1186/s13690-017-0252-2)
Supplement: Supplementary file 3 — (PDF 129 kb) [file 13690_2017_252_MOESM3_ESM.pdf]

ClinicalTrials.gov
Try our beta test site

Example: "Heart attack" AND "Los Angeles"
Search for studies: Search
Advanced Search | Help | Studies by Topic | Glossary

IMPORTANT: Listing of a study on this site does not reflect endorsement by the National Institutes of Health. Talk with a trusted healthcare professional before volunteering for a study. Read more...

Find Studies | About Clinical Studies | Submit Studies | Resources | About This Site

Home > Find Studies > Study Record Detail
Text Size

Protein Supplementation Impact On Body Muscle Mass And Fat Mass In Qataris Post Bariatric Surgery, Randomized Controlled Trials (Rct)

This study is currently recruiting participants. (see Contacts and Locations)

Verified March 2017 by Hamad Medical Corporation

Sponsor:
Hamad Medical Corporation

Information provided by (Responsible Party):
Hamad Medical Corporation

ClinicalTrials.gov Identifier:
NCT03147456

First received: April 18, 2017
Last updated: May 9, 2017
Last verified: March 2017
History of Changes

Full Text View | Tabular View | No Study Results Posted | Disclaimer | How to Read a Study Record

Purpose

One of the most common post-operative deficiencies or complications of bariatric surgery is protein malnutrition. It may lead to many predicaments such as malnutrition, vitamin, micro- and macronutrient deficiencies that can lead to deleterious consequences. A protein-rich diet make a person feel satiety and thereby the consumption will be low in overall energy intake. The objectives of our study are. The aim of this study is to assess the effect of protein supplementation on changes in health parameters such as fat mass, muscle mass, weigh change, protein (total and albumin), Vit B12, Zinc and Magnesium, in Qatari patients post bariatric surgery. Our participants are Qatari aged 18-45 years males and females recruited from the bariatric surgery centers of 2 major HMC hospitals (HGH hospital) and will be randomized to receive either the protein supplement (treatment group) or a dietary advice (control group). All participants will be equally followed and monitored for 3 months and data on the above parameters will be collected, together with other population characteristics, at Baseline, 1 month and 3 month. Data will then be analyzed using the most up-to-date SPSS statistical package to assess the effect of protein supplementation on the parameters of interest. Statistical measures will be carefully chosen to properly assess the difference in the treatment (protein supplementation) effect compared with the control (Dietary advice). Investigators hypothesize that Patients receiving protein supplement (intervention group) Compared with patient not on protein supplement (placebo group) will maintain weight loss and other essential health parameters.
