# Supplementary material for: The effect of protein supplementation on body muscle mass and fat mass in post-bariatric surgery: a randomized controlled trial (RCT) study protocol
Source: Arch Public Health. 2018 Jan 22;76:7. doi: 10.1186/s13690-017-0252-2 (PMC5789587; doi:10.1186/s13690-017-0252-2)
Supplement: Supplementary file 4 — (PDF 251 kb) [file 13690_2017_252_MOESM4_ESM.pdf]

Ref. No: MRC/0473/2017
Date: 8th March 2017

Ms. Sahar Dahawi Alshamari
Senior Clinical Dietitian
Dietetics & Nutrition
Surgery
HGH

Dear Ms. Sahar,

Subject: Research Proposal 16433/16 "Protein Supplementation Impact on Body Muscle Mass and Fat Mass in Qataris Post Bariatric Surgery, Randomized Controlled Trails (RCTs)"

The above titled Research Proposal submitted to the Medical Research Center has been approved to be conducted in HMC provided that the continuing approval from the HMC Institutional Review Board (IRB) is renewed as per the committee terms. The Research Center has acknowledged the IRB approval (Full Board) letter dated 16th February 2017.

This research study should be conducted in full accordance with all the applicable sections of the Rules and Regulations for Research at HMC and you should notify the Medical Research Center immediately of any proposed changes in study conduct that may affect the resource utilization at HMC. It is the Principal Investigator's responsibility to obtain review and continued approval if there is any modification to the approved protocol.

A study progress report should be submitted annually and a final report upon study's completion.

We wish you all success and await the results in due course.

Yours sincerely,

Prof. Ibrahim Janahi
Executive Director of Research
Medical Research Center

Cc:
1. Study Investigators
2. Chairman of HGH Research Committee

Tel: (+974)4439 2440
Fax: (+974) 4439 5402
research@hmc.org.qa

P.O.Box 3050
Doha, Qatar
www.hmc.org.qa
