# Supplementary material for: The effect of protein supplementation on body muscle mass and fat mass in post-bariatric surgery: a randomized controlled trial (RCT) study protocol
Source: Arch Public Health. 2018 Jan 22;76:7. doi: 10.1186/s13690-017-0252-2 (PMC5789587; doi:10.1186/s13690-017-0252-2)
Supplement: Supplementary file 5 — (PDF 439 kb) [file 13690_2017_252_MOESM5_ESM.pdf]

Dietary supplement

Supplement is a nutrition drink, designed for dietary management after bariatric surgery.

Dietary supplement instructions:

- Take one can daily, drink it in interval 5-6 times.
- Is ready to drink.
- Shake well before use.
- Don’t heat the supplement.
- Keep in refrigerator or in room temperature and when open the bottle it should be used within 24 hrs.

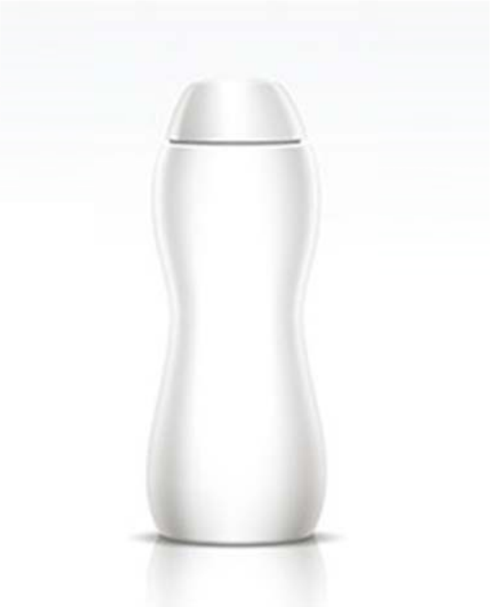

For any question call Bariatric and Metabolic Surgery Department

Phone: 44396792/44396794

HMC-IRB,16433/16,16Feb17-15Feb18

مؤسسة حمد الطبية
Hamad Medical Corporation
HEALTH • EDUCATION • RESEARCH صحة • تعليم • بحوث

المكمل الغذائي

المكمل الغذائي شراب مصمم لضبط التغدية بعد جراحة علاج السمنة.

تعليمات أخد المكملات الغذائية:

* اشرب عبوة واحدة يوميا على 5-6 فترات.

* العبوة جاهزه للشرب.

* قم بخض العبوة جيداً قبل الشرب.

* لا تقم بتسخين المكمل الغذائي.

* احفظه مبرداً أو في درجة حرارة الغرفة. يجب شرب العبوة خلال 24 ساعة بعد الفتح.

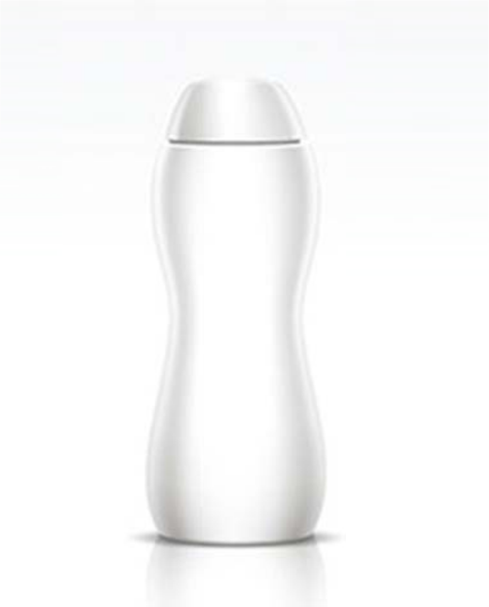

لأي استفسار اتصل بمركز جراحة السمنة والأيض

رقم الهاتف: 44396794/44396792

HMC-IRB,16433/16,16Feb17-15Feb18
